# Probabilistic Genotype-Phenotype Maps Reveal Mutational Robustness of RNA Folding, Spin Glasses, and Quantum Circuits

**Published:** 2023-01-04

**Authors:** Anna Sappington, Vaibhav Mohanty

**Affiliations:** 1 Harvard-MIT Health Sciences and Technology, Harvard Medical School, Boston, MA 02115 and Massachusetts Institute of Technology, Cambridge, MA 02139

## Abstract

Recent studies of genotype-phenotype (GP) maps have reported universally enhanced phenotypic robustness to genotype mutations, a feature essential to evolution. Virtually all of these studies make a simplifying assumption that each genotype maps deterministically to a single phenotype. Here, we introduce probabilistic genotype-phenotype (PrGP) maps, where each genotype maps to a vector of phenotype probabilities, as a more realistic framework for investigating robustness. We study three model systems to show that our generalized framework can handle uncertainty emerging from various physical sources: (1) thermal fluctuation in RNA folding, (2) external field disorder in spin glass ground state finding, and (3) superposition and entanglement in quantum circuits, which are realized experimentally on a 7-qubit IBM quantum computer. In all three cases, we observe a novel biphasic robustness scaling which is enhanced relative to random expectation for more frequent phenotypes and approaches random expectation for less frequent phenotypes.

## Introduction.—

Systems which take a sequence as input and nontrivially produce a structure, function, or behavior as output are ubiquitous throughout the sciences and engineering. In biological systems such as RNA folding [1–11], lattice protein folding [4], protein self-assembly [12, 13], and gene regulatory networks [14, 15], the relationship between genotype (stored biological information) and phenotype (observable or functional properties) can be structured as genotype-phenotype (GP) maps, which have a rich history of computational and analytical investigation [1–32]. Systems from physics and computer science have also been analyzed as GP maps, including the spin glass ground state problem [30], linear genetic programming [26], and digital circuits [31].

Despite being completely disparate systems, all of the GP maps above share a number of common structural features, most notably an enhanced robustness of the phenotypes to genotype mutations. Phenotypic *robustness ρ*_*n*_ of a phenotype *n* is the average probability that a single character mutation of a genotype *g* which maps to *n* does not change the resultant phenotype *n*, averaged over all genotypes *g* mapping to *n*. Random assignment of genotype to phenotype predicts that *ρ*_*n*_ ≈ *f*_*n*_ [4], where *f*_*n*_ is the fraction of genotypes that map to phenotype *n*. However, the systems mentioned above display substantially enhanced robustness, exhibiting the relationship *ρ*_*n*_ ≈ *a* + *b* log *f*_*n*_ ≫ *f*_*n*_ with system-dependent constants *a* and *b*. It has been shown that, in evolution, this enhanced robustness facilitates discovery of new phenotypes [11, 19, 20, 33] and is crucial for navigating fitness landscapes [5]. As a result, it is important to accurately quantify robustness and its relationship with phenotype frequency.

All of the GP map studies referenced above make the assumption that a genotype maps deterministically to a single phenotype. However, we argue that for most of the above systems, this is a major simplification. For instance, within a bulk sample of ~ *N* mammalian cells, we expect to find ~ *N* copies and ~ *N* * 10^4^ copies of a protein [34]. *In vitro*, such molecules often misfold [35], which is why cellular machinery exists to assist this folding and to degrade misfolded structures *in vivo*. By mapping a genotype to only the ground state energy structure, previous studies [1–11] make an implicit zero temperature approximation for the ensemble of molecules, even if the Gibbs free energy of an individual molecule itself is calculated within the folding software at finite temperature. Similarly, in studies of gene regulatory networks, spin glasses, linear genetic programs, and digital circuits, the systems investigated are small and do not interact with external networks or variables. These investigations assume that the environmental effect on the GP mapping of the subsystem of interest is static.

**FIG. 1.**
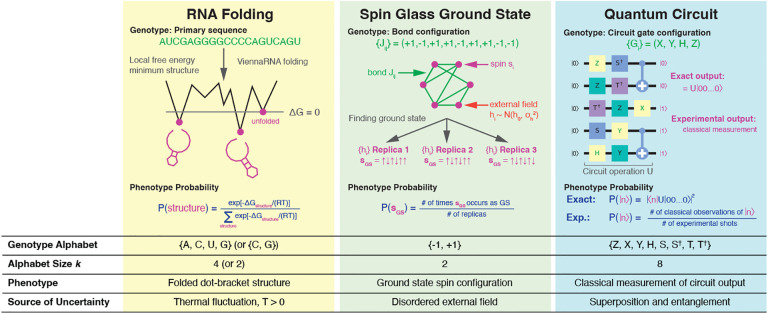
Schematic representations of the PrGP model systems studied in this work. Each system’s genotype, source of disorder, and method for calculating the phenotype probability vector are indicated.

In this Letter, we introduce probabilistic genotype-phenotype (PrGP) maps, in contrast to the above systems which we call deterministic genotype-phenotype (DGP) maps, which emerge as a limiting case of PrGP maps. The definitions of phenotypic robustness and transition probabilities retain the same physical meaning in PrGP maps as in DGP maps, and we emphasize that PrGP maps can handle disorder and uncertainty emerging from a variety of sources. To address the implicit zero temperature approximation in sequence-to-structure mappings (RNA, lattice protein folding, protein self-assembly), we study the folding of RNA primary sequences to a canonical ensemble of secondary structures corresponding to low-lying local free energy minima. To address external variable disorder with a known distribution, we study the zero temperature mapping of a spin glass bond configuration to its ground state with quenched external field disorder, building a phenotype probability vector using many replicas of the disordered field. This has implications for viral fitness landscape inference [36–40], where external fields, in part, model host immune pressure [39]. Lastly, to investigate inherent uncertainty in phenotypes, we introduce quantum circuit GP maps where uncertainty emerges from superposition and entanglement of classically measurable basis states. Our experimental realization of these quantum circuits on a 7-qubit IBM quantum computer also introduces measurement noise, which has a clear and unique effect on robustness. The PrGP map properties of the three model systems are summarized visually in Figure 1.

We observe that PrGP maps exhibit a novel biphasic scaling of robustness versus phenotype frequency which, for higher frequency phenotypes, resembles the *ρ*_*n*_ ∝ log *f*_*n*_ seen in DGP maps but is suppressed, and, for lower frequency phenotypes, settles closer to a linear relationship between *ρ*_*n*_ and *f*_*n*_, suggesting that the lowest frequency phenotypes either appear sporadically throughout the GP map or are uniformly scattered at low probabilities throughout the genotype domain.

## Theory.—

Let Ω(*g*) = *n* represent the mapping of genotype *g* to phenotype *n*, where *g* is an element of *S*_*ℓ*,*k*_, the set of all *k*^*ℓ*^ sequences of length *ℓ* drawn from an alphabet of *k* characaters. A generalization of robustness is the *transition probability ϕ*_*mn*_, the average probability that a single character mutation of a genotype mapping to phenotype *n* will change the phenotype to *m*, with the average taken over all genotypes mapping to *n*. For DGP maps, *ϕ*_*mn*_ is given by

(1) $$\phi_{mn}=\frac{\sum_{g\in S_{\ell ,k}}I[\Omega (g)=n]\sum_{h\in \text{nn}(g)}I[\Omega (h)=m]}{\ell (k-1)\sum_{g\in S_{\ell ,k}}I[\Omega (g)=n]},$$

where I[⋅] is the indicator function, and nn(*g*) is the single character mutational neighborhood of sequence *g*. For PrGP maps, we weaken the indicator I[Ω(g)=n] to a probability $p_{n}(g)\equiv \mathbb{P}[\Omega (g)=n]$, which allows us to write

(2) $$\phi_{mn}=\frac{\sum_{\{g,h\}\in \Delta_{\ell ,k}}{\left[p(g)⊗p(h)+{\left(p(g)⊗p(h)\right)}^{T}\right]}_{mn}}{\ell (k-1)k^{\ell }f_{n}},$$

where **p**(*g*) = (*p*_0_(*g*), *p*_1_(*g*), …) is the phenotype probability vector to which genotype *g* maps, and Δ_*ℓ*,*k*_ is the set of all *k*^*ℓ*^*ℓ*(*k* − 1)/2 unordered pairs of sequences in *S*_*ℓ*,*k*_ which differ by exactly one character. The phenotype probability vector obeys the normalization conditions $k^{\ell }f=\sum_{g\in S_{\ell ,k}}p(g)$ and $1=\sum_{n\in \{\text{phenotypes}\}}p_{n}(g)$ for all *g* ∈ *S*_*ℓ*,*k*_, and phenotype robustnesses are given by the diagonal of the transition probability matrix, *ρ*_*n*_ = *ϕ*_*nn*_. The phenotype entropy $S(g)=-\sum_{n\in \{\text{phenotypes}\}}p_{n}(g)\text{log}\;p_{n}(g)$ of a genotype *g* is also useful for quantifying *how* deterministic or probabilistic a PrGP map is.

In DGP maps, a random null model [4] for robustness can be built by randomly assigning genotype-phenotype pairings while keeping the frequencies **f** constant. As a result, the probability of a single character mutation leading to a change from phenotype *n* to phenotype *m* is approximately *ϕ*_*mn*_ ≈ *f*_*m*_ for all *m*. For PrGP maps, a naive expectation can be built by letting all phenotype probability vectors equal the frequency vector, **p**(*g*) = **f** for all genotypes *g*. From eq. (2), one finds that *ϕ*_*mn*_ = *f*_*m*_; thus, the two random expectations are the same, even though they physically represent different scenarios.

## RNA Secondary Structure Maps.—

In RNA folding DGP map studies [1–11], the global free energy minimum secondary structure (reported as a “dot-bracket” string indicating polymer connectivity) was calculated for every RNA sequence of fixed length drawn from the alphabet of the four canonical nucleotides {A, C, G, U} (alphabet size *k* = 4). Here, we are interested in not only the global free energy minimum structures but also the low-lying local minima, and we additionally investigate the temperature-dependent behavior of the robustness. We use the RNAsubopt program from the ViennaRNA package (version 2.4.17) [41] to calculate the secondary structures and associated Gibbs free energies for the local free energy minima within 6 kcal/mol of the global free energy minimum (or all the nonpositive free energy local minima, if the global minimum is greater than −6 kcal/mol). Because of the increased computational time required to discover all the local minima within an energy range, we use a reduced alphabet of {C, G} for our main simulations with sequence length *ℓ* = 20. A validation study with *ℓ* = 12 and the full *k* = 4 alphabet is reported in the Supplemental Material [42]. Simulations for the *ℓ* = 20, *k* = 2 trials were conducted at 20 °C, 37 °C (human body temperature), and 70 °C. We take the low-lying local free energy minima structures to comprise a canonical ensemble at the simulation temperature, so the probability of RNA sequence *g* mapping to secondary structure *n* is determined from *p*_*n*_(*g*) = *e*^−Δ*Gn/*(*RT*)^*/Z*, where *Z* normalizes the vector. We then calculate the robustness, transition probabilities, and phenotype entropy distributions as detailed in the previous section. The DGP map limits of the PrGP map are also plotted for each temperature.

**FIG. 2.**
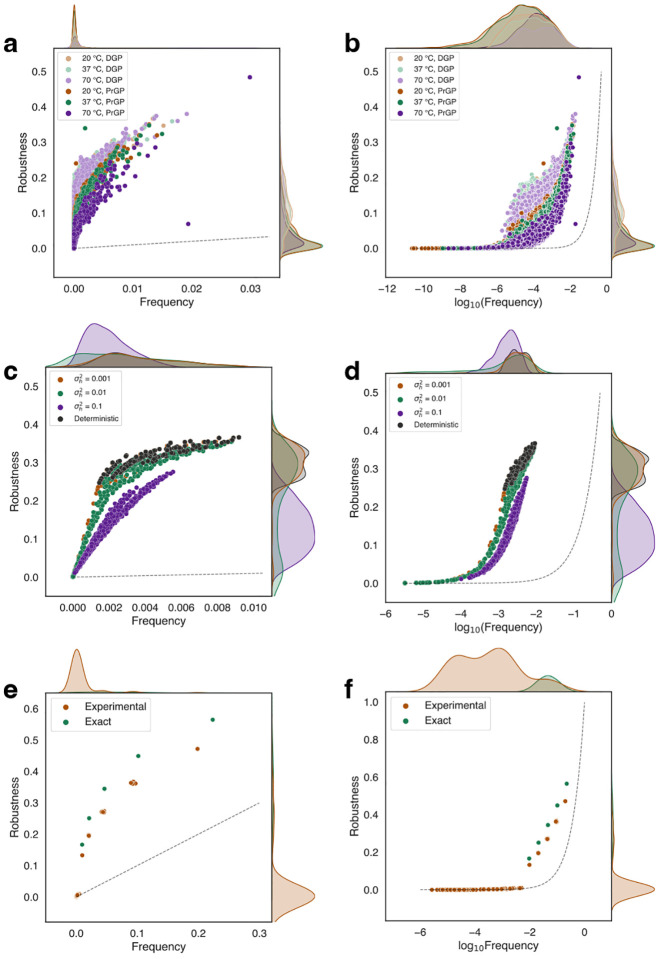
Plots of robustness versus (a,c,e) frequency and versus (b,d,f) log_10_(frequency) for (a,b) RNA folding in, (c,d) spin glass ground state, and (e,f) quantum circuit PrGP maps. The dashed line is the random null expectation *ρ*_*n*_ = *f*_*n*_.

In Figure 2(a–b), we plot the relationship between robustness and frequency for the *ℓ* = 20, *k* = 2 RNA PrGP map and for the DGP map limiting cases for each simulation temperature (see Supplemental Material [42] for Perason and Spearman correlations). The DGP maps confirm the results of refs. [3, 4], which emphasize that *ρ*_*n*_ ∝ log *f*_*n*_ for most phenotypes with significant elevation above the random null model [4] expectation. We find that there is little temperature dependence in DGP robustness calculations (see Supplemental Material [42]), suggesting that the effect of temperature does little to alter the exact ground state phenotype. However, our PrGP map results showcase a different robustness behavior. As the simulation temperature increases, there is a gradual but clear suppression of the robustness versus frequency relationship, as is apparent in both panels (a) and (b). We suggest this occurs due to two factors: firstly, though the ground state structure itself does not change much with temperature, the ground state becomes less stable relative to low-lying local minima, thereby increasing phenotype entropy, as evidenced by the entropy plots in the Supplemental Material [42]. As a result, for the corresponding **p**(*g*) ⊗ **p**(*h*) terms contributing to *ϕ*_*mn*_, probability mass is drawn away from the diagonals toward the off-diagonal transition probabilities. Secondly, as temperature increases, many low frequency (higher Δ*G*) phenotypes are discovered, increasing the number of phenotypes and drawing probability mass away from the more robust phenotypes.

For high frequency phenotypes, the PrGP map robustness is suppressed relative to the DGP map robustness, but is nonetheless substantially elevated above the random null expectation like in the DGP maps. However, for lower frequencies, the robustness behaves more like the random model; in the Supplemental Material, we see from a log-log plot of *ρ*_*n*_ versus *f*_*n*_ that robustness travels nearly parallel to the random null expectation, suggesting linear *ρ*_*n*_ ∝ *f*_*n*_ behavior up to a constant multiplicative factor. This biphasic robustness behavior becomes even clearer in the spin glass and quantum circuit PrGP maps. Off-diagonal transition probabilities maintained an approximate relationship *ϕ*_*mn*_ ∝ *f*_*m*_ for *m* ≠ *n*, in concordance with DGP maps (see Supplemental Material [42]).

## Spin Glass Ground State Maps.—

In a previous spin glass [43, 44] DGP map study [30], a zero temperature ±*J* spin glass on a random graph 𝒢(*V*, *E*) with Hamiltonian $H(s;J)=-\sum_{\{i,j\}\in E}J_{ij}s_{i}s_{j}-\sum_{i\in V}h_{i}s_{i}$ was considered. The genotype is the bond configuration where each *J*_*ij*_ ∈ {−1, +1}, and the phenotype is the ground state configuration where each *s*_*i*_ ∈ {−1, +1}. Degeneracies of the ground state were broken by the uniformly drawn, i.i.d. random external fields *h*_*i*_ ∈ [−10^−4^, 10^−4^] which were fixed for each simulation. In our spin glass PrGP map, we use a similar setup, but we are interested in the effect of external field disorder on robustness. We therefore incorporate the effects of Gaussian-distributed external fields $h_{i}\sim 𝒩\left(h_{0,i},\sigma_{h}^{2}\right)$, where the uniformly distributed means *h*_0,*i*_ ∈ [−0.1, 0.1] are fixed across all realizations of the disorder for each simulation. To obtain accurate robustness measurements, we exactly calculate every ground state for spin glasses with |*V*| = 9, and |*E*| = 15 by exhaustive enumeration. We examine the effect of external field disorder by simulating 450 replicas of {*h*_*i*_} with variances $\sigma_{h}^{2}=0.001$, 0.01, and 0.1 and fixed means {*h*_0,*i*_}. Phenotype probability vectors for each genotype *g* ≡ **J** were constructed by tallying and normalizing the number of appearances of each ground state across each replica. Graph topology 𝒢(*V*, *E*) corresponding to data presented here, as well as validation trial data, are in the Supplemental Material [42].

In Figure 2(c–d), we plot robustness versus frequency of each ground state for each external field variance $\sigma_{h}^{2}$ as well as the DGP map limiting case, which qualitatively reproduce the results of the earlier work [30] (see Supplemental Material [42] for Pearson and Spearman correlations). Trends similar to the RNA PrGP map are observed. Namely, as the disorder parameter (temperature for RNA and field variance for spin glasses) increases the uncertainty in the genotype-phenotype pairing, the phenotype entropy distribution shifts rightward (see Supplemental Material [42]), and the robustness versus frequency relationship becomes suppressed relative to the DGP map limit. Here, the spin glass results are more clearly suggestive of the proposed biphasic robustness relationship, especially apparent in panel (d). For the highest frequencies, the *ρ*_*n*_ is substantially enhanced above the random null expectation and behavior close to the deterministic limit is observed. However, for the smallest frequencies, nearly linear behavior is observed; in the log-log plot of *ρ*_*n*_ versus *f*_*n*_ (see Supplemental Material [42]), we see a strong sign that *ρ*_*n*_ ∝ *f*_*n*_, with the empirical robustness nearly parallel to the random expectation. As with the RNA folding PrGP maps, we suspect two causes which both contribute to this behavior: (1) as $\sigma_{h}^{2}$ increases, there is a higher chance of changing the ground state, which increases phenotype entropy, and (2) a larger number of spin configurations appear as ground states, but with low frequency, drawing away probability mass from the more frequent phenotypes.

## Quantum Circuit Maps.—

Although methods to evolve quantum circuits have been suggested [45], to our knowledge this work is the first to analyze the structural properties of quantum circuit GP maps. We generate random quantum circuits (see Supplemental Material for algorithm) with 7 qubits and 4 layers of gates. Circuits are randomly seeded with *CNOT* gates which cannot participate in the genotype, and the remaining spaces are filled with single-qubit gates drawn from the alphabet {*Z*, *X*, *Y*, *H*, *S*, *S*^†^, *T*, *T*^†^}. We choose *ℓ* = 4 of these gates to be variable gates which comprise the genotype. The input to the circuit is the prepared state |00…0〉 ≡ |0〉⊗⋯⊗|0〉, and the exact probability of classically measuring the basis state $|n\rangle ={⊗}_{|q_{i}\rangle \in \{|0\rangle ,|1\rangle \}}|q_{i}\rangle$ is *p*_*n*_(*g*) = |〈*n*|*U*(*g*)|00…0〉|^2^, where |*q*_*i*_〉 is the *i*-th qubit, and *U*(*g*) is the total circuit operation. We realize these quantum circuits on the *ibm_lagos* v1.2.0 quantum computer [42], one of the 7-qubit IBM Quantum Falcon r5.11H processors. Experimental phenotype probability vectors are constructed from tallying classical measurements from 1000 shots for each genotype. The circuits from our experimental trials are depicted in the Supplemental Material [42].

In Figure 2(e–f), we plot robustness versus frequency for each circuit output state, using both exact and experimental phenotype probability vectors for robustness calculations (see Supplemental Material [46] for Pearson and Spearman correlations and for data from additional validation trials). For the exact probabilities, the results in panel (f) strongly support the enhanced *ρ*_*n*_ ∝ log *f*_*n*_ scaling (Pearson *r* = 0.998). The spread of phenotypes in the frequency domain is due to superposition and/or entanglement; moreover, we see that many of the phentoypes are degenerate with identical frequency and robustness. This degeneracy is broken in our experimental measurements, which also exhibit measurement noise. Since we have finite shots, the degeneracies for the phenotypes observed in the exact case end up broken. The frequency and robustness of these logarithmically scaling phenotypes is suppressed relative to the exact case as probability density is drawn towards additional phenotypes, which are observed experimentally and which were not observed in the exact case. These appear due to measurement noise/decoherence effects in the physical system. The rightward shift of the phenotype entropy *S*(*g*) (see Supplemental Material [42]) further illustrates this effect.

Of the three systems investigated here, the quantum circuit PrGP map results in panel (f) are perhaps most illustrative of our suggested biphasic robustness scaling. The low frequency phenotypes which are introduced due to measurement noise in the experimental trials lie much closer to the random null expectation than the higher frequency phenotypes observed in the exact calculations, which rather scale with enhanced robustness similar to what is seen in standard DGP maps.

## Discussion.—

Compared to existing DGP maps, our introduction of PrGP maps not only allows for the inclusion of realistic, physical sources of disorder like thermal fluctuation and external variables, but it also permits the analysis of new systems like quantum circuits with inherent uncertainty built into the genotype-phenotype mapping and from measurement disorder. We emphasize the broad applicability of this framework to a vast array of systems across biology, physics, and computer science, and other disciplines for the analysis of robustness and stability. The proposed biphasic robustness scaling suggests that robustness of high frequency phenotypes in the DGP limit is suppressed in the PrGP formulation due to phenotype entropy increases and due to the discovery of new low frequency phenotypes. Moreover, low frequency phenotypes, which lie closer to the random null expectation, either appear randomly throughout genotype space (like in the DGP random null model), or they appear somewhat uniformly throughout a large portion of genotype space, but remain at low frequency (like in our new PrGP random null model). This scaling is observed in all three studied systems, despite being disparate, hinting at its universality. How this robustness trend affects navigability of (probabilistic) fitness landscapes is an important direction for further investigation. We also suggest that the mapping of genotypes to probability vectors instead of discrete phenotypes may facilitate the taking of gradients of, for instance, a negative loss-likelihood loss function in the process of learning PrGP or even DGP maps using statistical learning methods.

## Supplementary Material

1
