# Probabilistic Genotype-Phenotype Maps Reveal Mutational Robustness of RNA Folding, Spin Glasses, and Quantum Circuits

**Published:** 2024-08-22

**Authors:** Anna Sappington, Vaibhav Mohanty

**Affiliations:** 1 Department of Electrical Engineering and Computer Science, Massachusetts Institute of Technology, Cambridge, MA 02139; 2 Harvard-MIT Health Sciences and Technology, Harvard Medical School, Boston, MA 02115 and Massachusetts Institute of Technology, Cambridge, MA 02139; 3 Department of Chemistry and Chemical Biology, Harvard University, Cambridge, MA 02138

## Abstract

Recent studies of genotype-phenotype (GP) maps have reported universally enhanced phenotypic robustness to genotype mutations, a feature essential to evolution. Virtually all of these studies make a simplifying assumption that each genotype—represented as a sequence—maps deterministically to a single phenotype, such as a discrete structure. Here, we introduce probabilistic genotype-phenotype (PrGP) maps, where each genotype maps to a vector of phenotype probabilities, as a more realistic and universal language for investigating robustness in a variety of physical, biological, and computational systems. We study three model systems to show that PrGP maps offer a generalized framework which can handle uncertainty emerging from various physical sources: (1) thermal fluctuation in RNA folding, (2) external field disorder in spin glass ground state finding, and (3) superposition and entanglement in quantum circuits, which are realized experimentally on IBM quantum computers. In all three cases, we observe a novel biphasic robustness scaling which is enhanced relative to random expectation for more frequent phenotypes and approaches random expectation for less frequent phenotypes. We derive an analytical theory for the behavior of PrGP robustness, and we demonstrate that the theory is highly predictive of empirical robustness.

## Introduction.—

Systems which take a sequence as input and nontrivially produce a structure, function, or behavior as output are ubiquitous throughout the sciences and engineering. In biological systems such as RNA folding [1–11], lattice protein folding [4], protein self-assembly [12, 13], and gene regulatory networks [14, 15], the relationship between genotype (stored biological information) and phenotype (observable or functional properties) can be structured as genotype-phenotype (GP) maps, which have a rich history of computational and analytical investigation [1–34]. Systems from physics and computer science have also been analyzed as GP maps, including the spin glass ground state problem [30], linear genetic programming [26], and digital circuits [31].

Despite being completely disparate systems, all of the GP maps above share a number of common structural features, most notably an enhanced robustness of the phenotypes to genotype mutations. Phenotypic *robustness* $\rho_{n}$ of a phenotype n is the average probability that a single character mutation of a genotype g which maps to n does not change the resultant phenotype n, averaged over all genotypes g mapping to n. Random assignment of genotype to phenotype predicts that $\rho_{n}\approx f_{n}$ [4], where $f_{n}$ is the fraction of genotypes that map to phenotype n. However, the systems mentioned above display substantially enhanced robustness, exhibiting the relationship $\rho_{n}\approx a+b\log f_{n}\gg f_{n}$ with system-dependent constants a and b. It has been shown that, in evolution, this enhanced robustness facilitates discovery of new phenotypes [11, 19, 20, 35] and is crucial for navigating fitness landscapes [5]. As a result, it is important to accurately quantify robustness and its relationship with phenotype frequency.

All GP map studies referenced make the assumption that a genotype maps deterministically to a single phenotype. However, we argue that for most of the above systems, this is a major simplification. For instance, by mapping an RNA genotype to only the ground state energy structure, previous studies [1–11] make an implicit zero temperature approximation for the ensemble of molecules, even if the Gibbs free energy of an individual molecule itself is calculated within the folding software at finite temperature. Similarly, in studies of gene regulatory networks, spin glasses, linear genetic programs, and digital circuits, the systems investigated do not interact with external networks or variables. These investigations assume that the environmental effect on the GP mapping of the subsystem of interest is static.

In this Letter, we introduce probabilistic genotype-phenotype (PrGP) maps, in contrast to the above systems which we call deterministic genotype-phenotype (DGP) maps, which emerge as a limiting case of PrGP maps. The definitions of phenotypic robustness and transition probabilities retain the same physical meaning in PrGP maps as in DGP maps, and we emphasize that PrGP maps can handle disorder and uncertainty emerging from a variety of sources. To address the implicit zero temperature approximation in sequence-to-structure mappings (RNA, lattice protein folding, protein self-assembly), we study the folding of RNA primary sequences to a canonical ensemble of secondary structures corresponding to low-lying local free energy minima. To address external variable disorder with a known distribution, we study the zero temperature mapping of a spin glass bond configuration to its ground state with quenched external field disorder, building a phenotype probability vector using many replicas of the disordered field. This has implications for viral fitness landscape inference [36–40], where external fields, in part, model host immune pressure [39]. Lastly, to investigate inherent uncertainty in phenotypes, we introduce quantum circuit GP maps where uncertainty emerges from superposition and entanglement of classically measurable basis states. Our experimental realization of these quantum circuits on a 7-qubit IBM quantum computer also introduces measurement noise, which has a clear and unique effect on robustness. The PrGP map properties of the three model systems are summarized in Table I and visually in the Supplemental Material (SM) [41]. We observe that PrGP maps exhibit a novel biphasic scaling of robustness versus phenotype frequency which, for higher frequency phenotypes, resembles the $\rho_{n}\propto \log f_{n}$ seen in DGP maps but is suppressed, and, for lower frequency phenotypes, settles closer to a linear relationship between $\rho_{n}$ and $f_{n}$ or a more complex $f_{n}^{1-\eta }\left(a+b\log f_{n}\right)$ for 0<η<1.

### Theory.—

In this study, we focus on mappings of discrete genotypes, which can be written as sequences from a fixed alphabet, onto a discrete set of phenotypes. Let Ω(g)=n represent the mapping of genotype g to phenotype n, where g is an element of $S_{\ell ,k}$, the set of all $k^{\ell }$ sequences of length ℓ drawn from an alphabet of k characters. A generalization of robustness is the *transition probability* $\phi_{mn}$, the average probability that a single character mutation of a genotype mapping to phenotype n will change the phenotype to m, with the average taken over all genotypes mapping to n. For DGP maps, $\phi_{mn}$ is given by

(1) $$\phi_{mn}=\frac{\sum_{\left\{g|\text{Ω}(g)=n\right\}}|\{h\in \text{nn}(g)|\text{Ω}(h)=m\}|}{\ell (k-1)|\{g|\text{Ω}(g)=n\}|}.$$

where nn(g) is the single character mutational neighborhood of sequence g. For PrGP maps, we show in the SM [41] that the transition probability takes the form

(2) $$\phi_{mn}=\frac{\sum_{\{g,h\}\in \text{Δ}\ell ,k}{\left[p(g)⊗p(h)+{\left(p(g)⊗p(h)\right)}^{T}\right]}_{mn}}{\ell (k-1)k^{\ell }f_{n}},$$

where $p(g)=\left(p_{0}(g),p_{1}(g),\ldots \right)$ with $p_{n}(g)=\mathbb{P}[\text{Ω}(g)=n]$, the probability that genotype g maps to phenotype n. In the above equation, ${\text{Δ}}_{\ell ,k}$ is the set of all $k^{\ell }\ell (k-1)/2$ unordered pairs of sequences in $S_{\ell ,k}$ which differ by exactly one character. The phenotype probability vector obeys the normalization conditions $k^{\ell }f=\sum_{g\in S_{\ell ,k}}p(g)$ and $1=\sum_{n\in \{\text{phenotypes}\}}p_{n}(g)$ for all $g\in S_{\ell ,k}$, and phenotype robustnesses are given by the diagonal of the transition probability matrix, $\rho_{n}=\phi_{nn}$. We also are interested in the phenotype entropy $S(g)=-\sum_{n\in \{\text{phenotypes}\}}p_{n}(g)\log p_{n}(g)$, which quantifies the spread of a genotype’s mappings onto multiple phenotypes, and the genotype entropy $S_{n}^{\gamma }=-\sum_{g\in \{\text{genotypes}\}}\frac{p_{n}(g)}{f_{n}k^{\ell }}\log\frac{p_{n}(g)}{f_{n}k^{\ell }}$, which quantifies the spread of a phenotype across all genotypes.

In DGP maps, a random null model [4] for robustness can be built by randomly assigning genotype-phenotype pairings while keeping the frequencies f constant. As a result, the probability of a single character mutation leading to a change from phenotype n to phenotype m is approximately $\phi_{mn}\approx f_{m}$ for all m. For PrGP maps, a naive expectation can be built by letting all phenotype probability vectors equal the frequency vector, p(g)=f for all genotypes g. From eq. (2), one finds that $\phi_{mn}=f_{m}$; thus, the two random expectations are the same, even though they physically represent different scenarios.

A fundamental difference between PrGP maps and DGP maps is that DGP maps can have no frequencies lower than $k^{-\ell }$, but PrGP phenotypes in principle could have arbitrarily small frequencies. Thus, existing theory on DGP robustness [4, 12, 34] cannot be extended to PrGP phenotypes with sufficiently small frequencies. We thus develop a theory for PrGP robustness based on two key assumptions: (1) a phenotype n with frequency $f_{n}$ has probability mass evenly across $\xi_{n}(f_{n})k^{\ell }$ genotypes, and (2) the $\xi_{n}(f_{n})k^{\ell }$ genotypes would be a robustness-maximizing set in the DGP sense (i.e. maximizing eq. (1)). We discuss the validity of these assumptions later in the paper and extensively in the SM [41].

Two central results of this paper which follow (see SM [41] for a detailed derivation) from the above assumptions are the approximate PrGP robustness:

(3) $$\rho_{n}(f_{n})=\frac{f_{n}}{\xi_{n}\left(f_{n}\right)}\left[1+\frac{\log\xi_{n}\left(f_{n}\right)}{\ell \log k}\right],$$

and approximate upper bounds on the PrGP robustness given by the piecewise continuous function

(4) $$\rho_{n}^{\text{PrGP upper}}(f_{n})=\{\begin{array}{ll} \frac{f_{n}k^{\ell -1}}{\ell } & f_{n}\leq k^{1-\ell }\\ 1+\frac{\log f_{n}}{\ell \log k} & f_{n}\geq k^{1-\ell }. \end{array}$$

The upper bound illustrates two distinct scaling laws—namely, a DGP-like $\rho_{n}∼a+b\log f_{n}$ scaling for sufficiently large frequencies, and a null model-like linear scaling $\rho_{n}∼f_{n}$ for small frequencies. Since empirical DGP robustness often scales like a “suppressed” downscaling of the DGP maximum $\rho_{n}^{\text{DGP}\max}\approx 1+\frac{\log f_{n}}{\ell \log k}$, the biphasic scaling of the PrGP upper bound suggests that empirical PrGP robustness may also appear biphasic and suppressed relative to the upper bound. In the Results section we show that eq. (3), which is highly successful at recapitulating empirical robustness in 3 systems (RNA, spin glasses, quantum circuits), is amenable to further analytical approximation given system-specific information about $\xi_{n}(f_{n})$, yielding such biphasic scaling in different frequency regimes.

## Methods:

### RNA.—

In RNA folding DGP map studies [1–11], the global free energy minimum secondary structure (reported as a “dot-bracket” string indicating polymer connectivity) was calculated for every RNA sequence of fixed length drawn from the alphabet of the four canonical nucleotides {A,C,G,U} (alphabet size k=4). Here, we are interested in not only the global free energy minimum structures but also the low-lying local minima, and we additionally investigate the temperature-dependent behavior of the robustness. We use the RNAsubopt program from the ViennaRNA package (version 2.4.17) [42] to calculate the secondary structures and associated Gibbs free energies for the local free energy minima within 6 kcal/mol of the global free energy minimum (or all the nonpositive free energy local minima, if the global minimum is greater than −6 kcal/mol). Because of the increased computational time required to discover all the local minima within an energy range, we use a reduced alphabet of {C,G} for our main simulations with sequence length ℓ=20. A validation study with ℓ=12 and the full k=4 alphabet is reported in the SM [41]. Simulations for the ℓ=20, k=2 trials were conducted at 20 °C, 37 °C (human body temperature), and 70 °C. We take the low-lying local free energy minima structures to comprise a canonical ensemble at the simulation temperature, so the probability of RNA sequence g mapping to secondary structure n is determined from $p_{n}(g)=e^{-\text{Δ}G_{n}/(RT)}/Z$, where Z normalizes the vector.

### Spin Glasses.—

In a previous spin glass [43, 44] DGP map study [30], a zero temperature ±J spin glass on a random graph 𝓖(V,E) with Hamiltonian $H(s;J)=-\sum_{\{i,j\}\in E}J_{ij}s_{i}s_{j}-\sum_{i\in V}h_{i}s_{i}$ was considered. The genotype is the bond configuration where each $J_{ij}\in \{-1,+1\}$, and the phenotype is the ground state configuration where each $s_{i}\in \{-1,+1\}$. Degeneracies of the ground state were broken by the uniformly drawn, i.i.d. random external fields $h_{i}\in \left[-10^{-4},10^{-4}\right]$ which were fixed for each simulation. In our spin glass PrGP map, we use a similar setup, but we are interested in the effect of external field disorder on robustness. We therefore incorporate the effects of Gaussian-distributed external fields $h_{i}∼\text{𝓝}\left(h_{0,i},\sigma_{h}^{2}\right)$, where the uniformly distributed means $h_{0,i}\in [-0.1,0.1]$ are fixed across all realizations of the disorder for each simulation. To obtain accurate robustness measurements, we exactly calculate every ground state for spin glasses with |V|=9, and |E|=15 by exhaustive enumeration. We examine the effect of external field disorder by simulating 450 replicas of $\left\{h_{i}\right\}$ with variances $\sigma_{h}^{2}=0.001$, 0.01, and 0.1 and fixed means $\left\{h_{0,i}\right\}$. Phenotype probability vectors for each genotype g≡J were constructed by tallying and normalizing the number of appearances of each ground state across each replica. Graph topology 𝓖(V,E) corresponding to data presented here, as well as validation trial data, are in the SM [41].

### Quantum Circuits.—

Although methods to evolve quantum circuits have been suggested [45], to our knowledge this work is the first to analyze the structural properties of quantum circuit GP maps. We generate perform 7 trials in which we generate random quantum circuits (see SM for algorithm) with 7 qubits and 4 layers of gates; we also conduct an additional trial with 11 qubits and 4 layers of gates. Circuits are randomly seeded with *CNOT* gates which cannot participate in the genotype, and the remaining spaces are filled with single-qubit gates drawn from the alphabet {Z, X, Y, H, S, $S^{†}$, T, $T^{†}$}. We choose ℓ=4 (ℓ=5 for the 11 qubit trial) of these gates to be variable gates which comprise the genotype. The input to the circuit is the prepared state |00…0〉≡|0〉⊗⋯⊗|0〉, and the exact probability of classically measuring the basis state $|n\rangle ={⊗}_{|q_{i}\rangle \in \{|0\rangle ,|1\rangle \}}|q_{i}\rangle$ is $p_{n}(g)=|\langle n|U(g)|00\ldots {0\rangle |}^{2}$, where $|q_{i}\rangle$ is the i-th qubit, and U(g) is the total circuit operation. We realize these quantum circuits on the *ibm_lagos* v1.2.0 quantum computer [41], one of the 7-qubit IBM Quantum Falcon r5.11H processors. Experimental phenotype probability vectors are constructed from tallying classical measurements from 1000 shots for each genotype. The 11-qubit trial is conducted on a Qiskit Aer simulator instead of an experimental quantum computer, using the *ibm_brisbane* noise profile to simulate noise. The circuits from our experimental trials are depicted in the SM [41].

## Results and Discussion.—

**FIG. 1.**
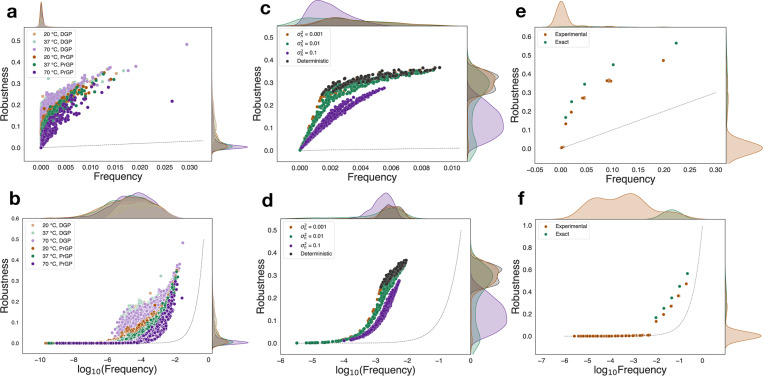
Plots of robustness versus (a,c,e) frequency and versus (b,d,f) log_10_(frequency) for (a,b) RNA folding in, (c,d) spin glass ground state, and (e,f) quantum circuit PrGP maps. The dashed line is the random null expectation $\rho_{n}=f_{n}$.

After computing the PrGP map data from the RNA, spin glass, and quantum circuit numerical experiments, we computed robustness, transition probabilities, phenotype entropy distributions, genotype entropy for each phenotype, as well as $\xi_{n}(f_{n})$ versus $f_{n}$ for each phenotype, all of which are plotted in the SM [41]. In Figure 1 we plot robustness versus frequency and versus log frequency for each of the 3 main systems studied (additional RNA, spin glass, and quantum circuit trials are in the SM [41]). Notable common features across all systems include robustness much higher than predicted by the null model for sufficiently large frequencies and a convergence toward the null model for sufficiently small frequencies. The RNA PrGP maps, all show suppressed robustness relative to their DGP counterparts, and this scaling is further suppressed as temperature increases. Similarly, in spin glasses, the DGP robustness is highest and closest to the log-linear relationship; the PrGP maps show increasingly suppressed scaling as the disorder variance is tuned higher. In quantum circuit PrGP maps, the trials with experimental or simulated noise show the appearance of a long tail of many new small-frequency phenotypes with, leading to the supression of the robustness of the large-frequency phenotypes with a maintenance of the approximate $\log f_{n}$ scaling.

From the phenotype entropy distributions in the SM [41], we see that as disorder parameters are increased (temperature, field variance, measurement noise), phenotype entropy distributions widen, meaning a genotype is more likely to have a broader distribution of phenotypes to which it maps. Similarly, genotype entropy, which is $S_{n}^{\gamma }=\ell \log k+\log f_{n}$, for DGP maps, maintains similar scaling but is suppressed in PrGP maps, meaning that a phenotype with fixed frequency is likely to be spread out over more genotypes in the PrGP case than in the DGP case.

**FIG. 2.**
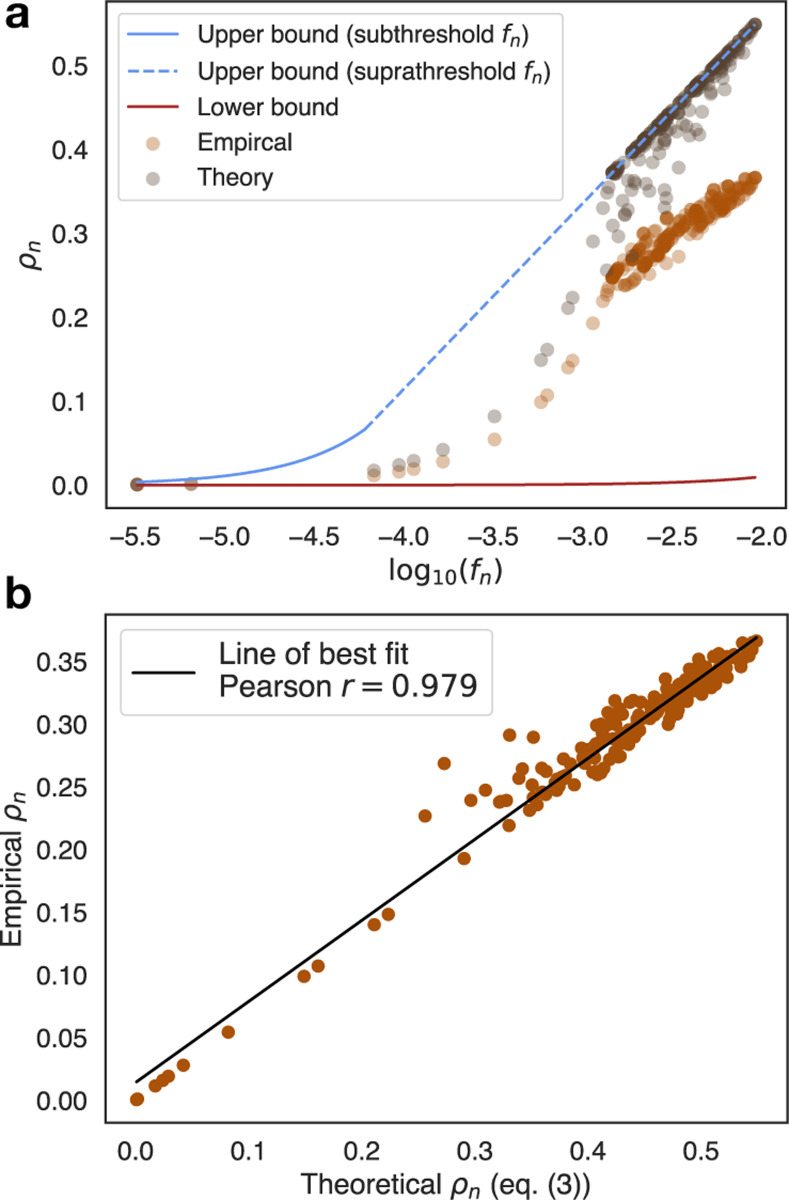
(a) Plot of $\log_{10}\left(f_{n}\right)$ versus $\rho_{n}$, where $\rho_{n}$ has either been computed empirically from the experimental data or theoretically from eq. (3) for the spin glass system $\left(\sigma_{h}^{2}=0.001\right)$. Includes upper bounds from eq. (4) and null model. (b) Scatter plot of theoretical $\rho_{n}$ versus empirical $\rho_{n}$ for the spin glass system $\left(\sigma_{h}^{2}=0.001\right)$ with Pearson r=0.979.

Notably, we also compute $\xi_{n}\left(f_{n}\right)$ for all systems and predict robustness by directly plugging in $\xi_{n}\left(f_{n}\right)$ and $f_{n}$ into eq. (3). We show an example plot of the thoeretical robustness, empirical robustness, null model, and upper bound for spin glasses with $\sigma_{h}^{2}=0.001$ in Figure 2(a). Not only does the the theoretical robustness, given only $\xi_{n}\left(f_{n}\right)$ and $f_{n}$, recapitulate the salient scaling behavior of the empirical robustness, as shown in Figure 2(b), but the Pearson correlation between the predicted and empirical robustness is r=0.979; in the SM [41], we show that the Pearson correlations from robustness obtained from eq. (3) for all systems studied here ranged from 0.859–0.9996 and outperformed the null model and DGP maximum robustness formulas across all systems, illustrating the success of eq. (3). While the Pearson correlations are high, the prediction from eq. (3) varies by additive or multiplicative constant factors likely due to violation of one or both assumptions mentioned in the Theory section. As disorder parameters increase, these violations become more prominent and eq. (3) and the null model converge toward similar performance (see Table S1 in SM [41]), meaning that biphasic scaling starts to fade away in favor of null model-like linear scaling when there is too much disorder. In all cases though, the theory remains highly predictive.

Interestingly, we find that $\xi_{n}\left(f_{n}\right)$ tends to obey a power law with $\xi_{n}\left(f_{n}\right)\approx \alpha f_{n}^{\eta }$ (generally with 0≤η≤1) over many orders of magnitude, for all 3 systems, though with slightly differing behavior. This leads to a robustness expression

(5) $$\rho_{n}=\frac{f_{n}^{1-\eta }}{\alpha }\left(1+\frac{\log\alpha }{\ell \log k}+\eta \frac{\log f}{\ell \log k}\right).$$

Notably, when η=0 (e.g. for small frequencies in the spin glass $\sigma_{h}^{2}=0.001$ case and for all frequencies in the $\sigma_{h}^{2}=0.1$ case), eq. (5) becomes $\rho_{n}∼f_{n}$. In RNA and quantum circuits, a sublinear power law 0<η<1 is observed, resulting in $\rho_{n}∼f_{n}^{1-\eta }\left(a+b\eta \log f_{n}\right)$. Lastly, for sufficiently large frequencies, substituting $x_{n}=\log f_{n}$ into eq. (5), the leading order behavior for small $x_{n}$ becomes $\rho_{n}∼a+\eta b\log f_{n}$. The theory suggests that the biphasic behavior is characteristic of the form of eq. (3), as there are other functional forms for $\xi_{n}\left(f_{n}\right)$ which produce the same or similar behaviors in different regimes (see SM [41]).

Compared to existing DGP maps, PrGP maps not only allow for the inclusion of realistic, physical sources of disorder like thermal fluctuation and external variables, but they also permit the analysis of new systems like quantum circuits with inherent uncertainty. We emphasize the broad applicability of this framework to a vast array of systems across biology, physics, and computer science, and other disciplines for the analysis of robustness and stability. The analytical theory introduced here indicates a link between a phenotype and how it is spread over the genotypes. Given the empirical observation of a power law relationship between the number of unique genotypes over which the phenotype is spread and the frequency of the phenotype, we can show that for high frequencies loglinear (DGP-like) robustness is expected, while for small frequencies linear or a more complex $f_{n}^{1-\eta }\left(a+b\eta \log f_{n}\right)$ is expected, depending on system specific information. Moreover, as disorder in a system is increased, phenotypes spread over a larger number of genotypes, leading to increasingly suppressed robustness and more null model-like behavior. Most notably, our theory in eq. (3) is highly successful, measured by Pearson correlation, in predicting empirical robustness across all systems.

The scaling we observe empircally and justify theoretically in this letter is observed in all three studied systems, despite being disparate, hinting at its universality. How this robustness trend affects navigability of (probabilistic) fitness landscapes is an important direction for further investigation. We also suggest that the mapping of genotypes to probability vectors instead of discrete phenotypes may facilitate the taking of gradients of, for instance, a negative loss-likelihood loss function in the process of learning PrGP or even DGP maps using statistical learning methods. Specifically, one might model a GP map using a graph neural network [46] and predict the phenotype or related properties of neighboring nodes. Such a model may ultimately aid in inferring fitness landscapes from limited initial GP data [47–49].

## Supplementary Material

Supplement 1

## Figures and Tables

**TABLE I.** Overview of the genotypes and phenotypes of each PrGP system, as well as their respective sources of uncertainty.

System	Genotype Alphabet	Alphabet size k	Phenotype	Source of Uncertainty
RNA folding	{A, U, G, C} (or {G, C})	4 (or 2)	Folded dot-bracket structure	Thermal fluctuation, T>0
Spin glass ground state	{−1, +1}	2	Ground state spin configuration	Disordered external field
Quantum circuit	{Z, X, Y, H, S, S^†^, T, T^†^}	8	Classical measurement of circuit output	Superposition and entanglement
