# Probabilistic Genotype-Phenotype Maps Reveal Mutational Robustness of RNA Folding, Spin Glasses, and Quantum Circuits

**Published:** 2025-01-03

**Authors:** Anna Sappington, Vaibhav Mohanty

**Affiliations:** 1 Department of Electrical Engineering and Computer Science, Massachusetts Institute of Technology, Cambridge, MA 02139; 2 Program for Health Sciences and Technology, Harvard Medical School, Boston, MA 02115 and Massachusetts Institute of Technology, Cambridge, MA 02139; 3 Harvard/MIT MD-PhD Program, Harvard Medical School, Boston, MA 02115 and Massachusetts Institute of Technology, Cambridge, MA 02139; 4 Department of Chemistry and Chemical Biology, Harvard University, Cambridge, MA 02138

## Abstract

Recent studies of genotype-phenotype (GP) maps have reported universally enhanced phenotypic robustness to genotype mutations, a feature essential to evolution. Virtually all of these studies make a simplifying assumption that each genotype—represented as a sequence—maps deterministically to a single phenotype, such as a discrete structure. Here, we introduce probabilistic genotype-phenotype (PrGP) maps, where each genotype maps to a vector of phenotype probabilities, as a more realistic and universal language for investigating robustness in a variety of physical, biological, and computational systems. We study three model systems to show that PrGP maps offer a generalized framework which can handle uncertainty emerging from various physical sources: (1) thermal fluctuation in RNA folding, (2) external field disorder in spin glass ground state finding, and (3) superposition and entanglement in quantum circuits, which are realized experimentally on IBM quantum computers. In all three cases, we observe a novel biphasic robustness scaling which is enhanced relative to random expectation for more frequent phenotypes and approaches random expectation for less frequent phenotypes. We derive an analytical theory for the behavior of PrGP robustness, and we demonstrate that the theory is highly predictive of empirical robustness.

## I. INTRODUCTION

Systems which take a sequence as input and nontrivially produce a structure, function, or behavior as output are ubiquitous throughout the sciences and engineering. In biological systems such as RNA folding [1–11], lattice protein folding [4], protein self-assembly [12, 13], and gene regulatory networks [14, 15], the relationship between genotype (stored biological information) and phenotype (observable or functional properties) can be structured as genotype-phenotype (GP) maps, which have a rich history of computational and analytical investigation [1–34]. Systems from physics and computer science have also been analyzed as GP maps, including the spin glass ground state problem [30], linear genetic programming [26], and digital circuits [31].

Despite being completely disparate systems, all of the GP maps above share a number of common structural features, most notably an enhanced robustness of the phenotypes to genotype mutations. Phenotypic *robustness* $\rho_{n}$ of a phenotype n is the average probability that a single character mutation of a genotype g which maps to n does not change the resultant phenotype n, averaged over all genotypes g mapping to n. A completely random assignment of genotype to phenotype predicts that $\rho_{n}\approx f_{n}$ [4], where $f_{n}$ is the fraction of genotypes that map to phenotype n. However, the systems mentioned above display a surprising and substantially enhanced robustness, exhibiting the relationship $\rho_{n}\approx a+b\;\text{log}\;f_{n}\gg f_{n}$ with system-dependent constants a and b, meaning that even for rare phenotypes, a small changes to the genotype do not necessarily result in change of the phenotype. It has been shown that, in evolution, this enhanced robustness facilitates discovery of new phenotypes [11, 19, 20, 35] and is crucial for navigating fitness landscapes [5]. As a result, it is important to accurately quantify robustness and its relationship with phenotype frequency.

All GP map studies referenced above, spanning several decades of research, make the assumption that a genotype maps deterministically to a single phenotype. However, we argue that for most of the above systems, this is a major simplification. For instance, by mapping an RNA genotype to only the ground state energy structure, previous studies [1–11] make an implicit zero temperature approximation for the ensemble of molecules, even if the Gibbs free energy of an individual molecule itself is calculated within the folding software at finite temperature. Similarly, in studies of gene regulatory networks, spin glasses, linear genetic programs, and digital circuits, the systems investigated do not interact with external networks or variables. These investigations assume that the environmental effect on the GP mapping of the subsystem of interest is static. Probabilistic mappings from genotype to phenotype have certainly existed in many realms of science, such as probabilistic classifications of images by neural networks (i.e. sequences of pixel intensities mapping to probabilities of classes). However, the literature still lacks a unifying framework to analyze the single-character mutational robustness of these maps, among other properties, in the way that there already exists a universal language for the deterministic GP (DGP) maps mentioned above.

In this article, we introduce probabilistic genotype-phenotype (PrGP) maps—in contrast to the above systems which we call DGP maps—as a universal framework for analyzing the mutational robustness of sequence-to-discrete classifications. DGP maps thereby emerge as the limiting case of PrGP maps in each genotype or sequence maps to a single phenotype with probability 1 and all other phenotypes with probability 0. The definitions of phenotypic robustness and transition probabilities retain the same physical meaning in PrGP maps as in DGP maps, and we emphasize that PrGP maps can handle disorder and uncertainty emerging from a variety of sources.

**FIG. 1.**
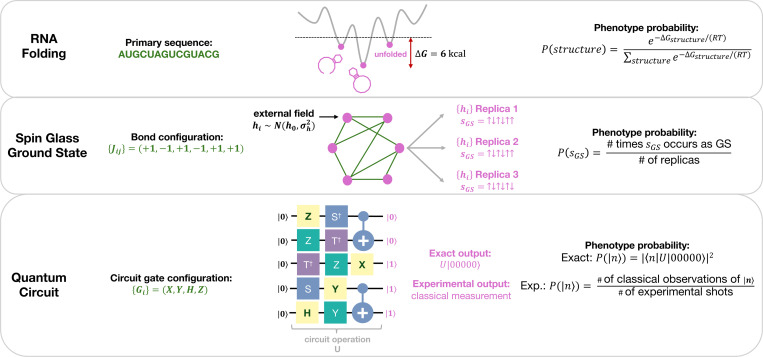
Schematic representations of the PrGP model systems studied in this work. For each system, its respective genotype (green), a visualization of the system, its phenotypes (pink), and its method for calculating the phenotype probability vector are shown. For RNA folding, the genotype is a primary sequence of nucleotides and the phenotype is the folded dot-bracket structure. For spin glass ground states, the genotype is a bond configuration. Each spin $s_{i}$ (pink dots) are connected via this bond configuration $J_{ij}$ (green lines) and a disordered external field $h_{i}$ is applied. The phenotype is the fraction of replicas in which each ground state appears. For quantum circuits, the genotype consists of a subset of gates from a random circuit. The circuit is given a set input state, |00…0〉, and the exact phenotype probability vector is the probability of classically measuring each basis state, $p_{n}\left(g\right)={\left|\langle n|U\left(g\right)|00\ldots 0\rangle \right|}^{2}$, where U(g) is the circuit operation as a function of the genotype g. The experimental phenotype probability vector is computed from tallying classically measured states from 1000 experimental shots on a quantum computer.

To address the implicit zero temperature approximation in sequence-to-structure mappings (RNA, lattice protein folding, protein self-assembly), we study the folding of RNA primary sequences to a canonical ensemble of secondary structures corresponding to low-lying local free energy minima. To address external variable disorder with a known distribution, we study the zero temperature mapping of a spin glass bond configuration to its ground state with quenched external field disorder, building a phenotype probability vector using many replicas of the disordered field. This has implications for viral fitness landscape inference [36–40], where external fields, in part, model host immune pressure [39]. Lastly, to investigate inherent uncertainty in phenotypes, we introduce quantum circuit GP maps where uncertainty emerges from superposition and entanglement of classically measurable basis states. Our experimental realization of these quantum circuits on a 7-qubit IBM quantum computer also introduces measurement noise, which has a clear and unique effect on robustness. The PrGP map properties of the three model systems are summarized in Table I and visually in Figure 1. We observe empirically that PrGP maps exhibit a novel biphasic scaling of robustness versus phenotype frequency which, for higher frequency phenotypes, resembles the $\rho_{n}\propto \text{log}f_{n}$ seen in DGP maps but is suppressed, and, for lower frequency phenotypes, settles closer to a linear relationship between $\rho_{n}$ and $f_{n}$. We then develop a set of approximations which yield an analytically solvable model of robustness which predicts empirical robustness well outside the approximation regime.

## II. THEORY

In this study, we focus on mappings of discrete genotypes, which can be written as sequences from a fixed alphabet, onto a discrete set of phenotypes (i.e. *discrete-to-discrete GP maps*).

Let Ω(g)=n represent the mapping of genotype g to phenotype n, where g is an element of $S_{\ell ,k}$, the set of all $k^{\ell }$ sequences of length ℓ drawn from an alphabet of k characters. A generalization of robustness is the *transition probability* $\phi_{mn}$, the average probability that a single character mutation of a genotype mapping to phenotype n will change the phenotype to m, with the average taken over all genotypes mapping to n. For DGP maps, $\phi_{mn}$ is given by

(1) $$\phi_{mn}=\frac{\sum_{\left\{g|\text{Ω}(g)=n\right\}}\left|\{h\in \text{nn}(g)|\text{Ω}(h)=m\}\right|}{\ell (k-1)|\{g|\text{Ω}(g)=n\}|}.$$

where nn(g) is the single character mutational neighborhood of sequence g. In this formula, the numerator is counting how many single-character mutational neighbors of some genotype g (which maps to phenotype m) map to phenotype n. This means that the robustness can be written as the special case m=n:

(2) $$\rho_{n}=\phi_{nn}=\frac{\sum_{\left\{g|\text{Ω}(g)=n\right\}}\left|\{h\in \text{nn}(g)|\text{Ω}(h)=n\}\right|}{\ell (k-1)k^{\ell }f_{n}}.$$

For PrGP maps, we show in Appendix A that the transition probability formula becomes a modified version of eq. (1) in which we take a *weighted* sum in the numerator. In particular, we have

(3) $$\phi_{mn}=\frac{\sum_{\{g,h\}\in {\text{Δ}}_{\ell ,k}}{\left[\text{p}(g)⊗\text{p}(h)+{\left(\text{p}(g)⊗\text{p}(h)\right)}^{T}\right]}_{mn}}{\ell (k-1)k^{\ell }f_{n}},$$

where $\text{p}(g)=\left(p_{0}(g),p_{1}(g),\ldots \right)$ with $p_{n}(g)=\mathbb{P}[\text{Ω}(g)=n]$, the probability that genotype g maps to phenotype n. Again, the robustness $\rho_{n}=\phi_{nn}$. In the above equation, ${\text{Δ}}_{\ell ,k}$ is the set of all $k^{\ell }\ell (k-1)/2$ unordered pairs of sequences in $S_{\ell ,k}$ which differ by exactly one character. The phenotype probability vector obeys the normalization conditions $k^{\ell }\text{f}=\sum_{g\in S_{\ell ,k}}\text{p}(g)$ and $1=\sum_{n\in \{\text{phenotypes}\}}p_{n}(g)$ for all $g\in S_{\ell ,k}$, and phenotype robustnesses are given by the diagonal of the transition probability matrix, $\rho_{n}=\phi_{nn}$. We also are interested in the phenotype entropy $S(g)=-\sum_{n\in \{\text{phenotypes}\}}p_{n}(g)\text{log}p_{n}(g)$, which quantifies the spread of a genotype’s mappings onto multiple phenotypes, and the genotype entropy

(4) $$S_{n}^{\gamma }=-\sum_{g\in \{\text{genotypes}\}}\frac{p_{n}(g)}{f_{n}k^{\ell }}\text{log}\frac{p_{n}(g)}{f_{n}k^{\ell }},$$

which quantifies the spread of a phenotype across all genotypes. In particular, we will show that the genotype entropy can be useful for estimating robustness.

In DGP maps, a random null model [4] for robustness can be built by randomly assigning genotype-phenotype pairings while keeping the frequencies f constant. As a result, the probability of a single character mutation leading to a change from phenotype n to phenotype m is approximately $\phi_{mn}\approx f_{m}$ for all m. For PrGP maps, a naive expectation can be built by letting all phenotype probability vectors equal the frequency vector, p(g)=f for all genotypes g. From eq. (3), one finds that $\phi_{mn}=f_{m}$; thus, the two random expectations are the same, even though they physically represent different scenarios.

A fundamental difference between PrGP maps and DGP maps is that DGP maps can have no frequencies lower than $k^{-\ell }$, but PrGP phenotypes in principle could have arbitrarily small frequencies, suggesting that the PrGP robustness curve has a tail, representing very rare phenotypes, that is not necessarily predictable from existing DGP robustness theory [4, 12, 34]. In this work, we show that under two approximations, the robustness becomes analytically solvable in terms of the phenotype frequency $f_{n}$ and genotype entropy $S_{n}^{\gamma }$. Although these approximations make specific assumptions on the shape and distribution of the phenotype’s probability over genotypes which do not necessarily coincide with empirical distributions, we demonstrate in the Results section that, amazingly, the resulting robustness formula below exhibits exceptional predictive performance well outside of the approximations made on the phenotype structure on all 3 systems empirically studied.

The two key approximations are as follows: (1) a phenotype n with frequency $f_{n}$ has probability mass evenly spread across a fixed number of genotypes, and (2) that fixed number of genotypes would be a robustness-maximizing set in the DGP sense (i.e. maximizing eq. (1)). Two central results of this paper which follow from the above assumptions (see Appendix B for the derivation) are the approximate PrGP robustness as a function of the phenotype frequency $f_{n}$ and the genotype entropy $S_{n}^{\gamma }$:

(5) $$\rho_{n}\left(f_{n},S_{n}^{\gamma }\right)\approx \frac{k^{\ell }f_{n}S_{n}^{\gamma }e^{-S_{n}^{\gamma }}}{\ell \text{log}k},$$

and approximate upper bounds on the PrGP robustness given by the piecewise continuous function

(6) $$\rho_{n}^{\text{PrGP}\;\text{upper}}\left(f_{n}\right)\approx \{\begin{array}{cc} \frac{f_{n}k^{\ell -1}}{\ell } & f_{n}\leq k^{1-\ell }\\ 1+\frac{\text{log}f_{n}}{\ell \text{log}k} & f_{n}\geq k^{1-\ell }. \end{array}$$

The upper bound illustrates two distinct scaling laws—namely, a DGP-like $\rho_{n}∼a+b\text{log}f_{n}$ scaling for sufficiently large frequencies, and a null model-like linear scaling $\rho_{n}∼f_{n}$ for small frequencies. Since empirical DGP robustness often scales like a “suppressed” downscaling of the DGP maximum $\rho_{n}^{\text{DGP}\max}\approx 1+\frac{\text{log}f_{n}}{\ell \text{log}k}$, the biphasic scaling of the PrGP upper bound suggests that empirical PrGP robustness may also appear biphasic and suppressed relative to the upper bound.

The upper bounds here are approximate because we rely on the genotypes forming a robustness-maximizing set (in the DGP sense, meaning the genotypes tend to be clustered in the sequence space) before we optimize or approximate the spread of the phenotype’s probability mass over those genotypes. Although this may very well be an exact and/or tight bound, we do not prove its tightness here. However, we discuss specific cases of how each of these upper bounds can be achieved in real phenotypes: for rare phenotypes in the “tail” of the robustness upper bound $\left(f_{n}\leq k^{1-\ell }\right)$, we find that phenotypes which maximize the robustness are spread evenly over exactly k genotypes whose sequences all differ at exactly one character in the sequence, with each of those genotypes having probability $p_{n}(g)=f_{n}k^{\ell -1}$ of mapping to the phenotype. For more common phenotypes with $f_{n}\geq k^{1-\ell }$, an instructive example appears when a phenotype frequency is $f_{n}=k^{r-\ell }$ for some integer 1≤r≤ℓ. We consider ℓ−r of the ℓ sites in the sequence to be “constrained” (using terminology from ref. [12]), meaning that mutating any of those sites will lead to a change in phenotype. The remaining r sites are “unconstrained,” meaning that phenotypes at those sites will not lead to any change in phenotype. If the phenotype probability is $p_{n}(g)=1$ at all $k^{r}$ of those genotypes, the robustness is exactly equal to the DGP robustness and is simply equal to the probability of mutating an unconstrained site, namely $\rho_{n}=r/\ell$, which attains the upper bound in eq. (6). For frequencies in which $f_{n}=k^{r-\ell }$ for some non-integer value of r, finding a configuration in which robustness maximized is nontrivial; for DGP maps, this problem was solved in ref. [34] and the maximal robustness was found to be given by a fractal curve though it asymptotically behaves like eq. (6) with small corrections. The exact upper bound for PrGP maps remains an open problem.

In the Results section, we show that eq. (5), which is highly successful at recapitulating empirical robustness in 3 systems (RNA, spin glasses, quantum circuits), is amenable to further analytical approximation given system-specific information about the genotype entropy $S_{n}^{\gamma }$, yielding such biphasic scaling in different frequency regimes.

## III. NUMERICAL METHODS

### A. RNA

In RNA folding DGP map studies [1–11], the global free energy minimum secondary structure (reported as a “dot-bracket” string indicating polymer connectivity) was calculated for every RNA sequence of fixed length drawn from the alphabet of the four canonical nucleotides {A,C,G,U} (alphabet size k=4). Here, we are interested in not only the global free energy minimum structures but also the low-lying local minima, and we additionally investigate the temperature-dependent behavior of the robustness. We use the RNAsubopt program from the ViennaRNA package (version 2.4.17) [41] to calculate the secondary structures and associated Gibbs free energies for the local free energy minima within 6 kcal/mol of the global free energy minimum (or all the nonpositive free energy local minima, if the global minimum is greater than −6 kcal/mol). Because of the increased computational time required to discover all the local minima within an energy range, we use a reduced alphabet of {C,G} for our main simulations with sequence length ℓ=20. A validation study with ℓ=12 and the full k=4 alphabet is reported in the Supplementary Material (SM) [42]. Simulations for the ℓ=20, k=2 trials were conducted at 20°C, 37 °C (human body temperature), and 70 °C. We take the low-lying local free energy minima structures to comprise a canonical ensemble at the simulation temperature, so the probability of RNA sequence g mapping to secondary structure n is determined from $p_{n}(g)=e^{-\text{Δ}G_{n}/(RT)}/Z$, where Z normalizes the vector.

### B. Spin Glasses

In a previous spin glass [43, 44] DGP map study [30], a zero temperature ±J spin glass on a random graph 𝒢(V,E) with Hamiltonian $H(\text{s};\text{J})=-\sum_{\{i,j\}\in E}J_{ij}s_{i}s_{j}-\sum_{i\in V}h_{i}s_{i}$ was considered. The genotype is the bond configuration where each $J_{ij}\in \{-1,+1\}$, and the phenotype is the ground state configuration where each $s_{i}\in \{-1,+1\}$. Degeneracies of the ground state were broken by the uniformly drawn, i.i.d. random external fields $h_{i}\in \left[-10^{-4},10^{-4}\right]$ which were fixed for each simulation. In our spin glass PrGP map, we use a similar setup, but we are interested in the effect of external field disorder on robustness. We therefore incorporate the effects of Gaussian-distributed external fields $h_{i}∼𝒩\left(h_{0,i},\sigma_{h}^{2}\right)$, where the uniformly distributed means $h_{0,i}\in [-0.1,0.1]$ are fixed across all realizations of the disorder for each simulation. To obtain accurate robustness measurements, we exactly calculate every ground state for spin glasses with |V|=9, and |E|=15 by exhaustive enumeration. We examine the effect of external field disorder by simulating 450 replicas of $\left\{h_{i}\right\}$ with variances $\sigma_{h}^{2}=0.001$, 0.01, and 0.1 and fixed means $\left\{h_{0,i}\right\}$. Phenotype probability vectors for each genotype g≡J were constructed by tallying and normalizing the number of appearances of each ground state across each replica. Graph topology 𝒢(V,E) corresponding to data presented here, as well as validation trial data, are in the SM [42].

### C. Quantum Circuits

Although methods to evolve quantum circuits have been suggested [45], to our knowledge this work is the first to analyze the structural properties of quantum circuit GP maps. We generate perform 7 trials in which we generate random quantum circuits (see SM for algorithm) with 7 qubits and 4 layers of gates; we also conduct an additional trial with 11 qubits and 4 layers of gates. Circuits are randomly seeded with *CNOT* gates which cannot participate in the genotype, and the remaining spaces are filled with single-qubit gates drawn from the alphabet $\left\{Z,X,Y,H,S,S^{†},T,T^{†}\right\}$. We choose ℓ=4 (ℓ=5 for the 11 qubit trial) of these gates to be variable gates which comprise the genotype. The input to the circuit is the prepared state |00…0〉≡|0〉⊗⋯⊗|0〉, and the exact probability of classically measuring the basis state $|n\rangle ={⊗}_{|q_{i}\rangle \in \left\{|0\rangle ,|1\rangle \right\}}|q_{i}\rangle$ is $p_{n}\left(g\right)={\left|\langle n|U\left(g\right)|00\ldots 0\rangle \right|}^{2}$, where $|q_{i}\rangle$ is the i-th qubit, and U(g) is the total circuit operation. We realize these quantum circuits on the *ibm_lagos* v1.2.0 quantum computer [42], one of the 7-qubit IBM Quantum Falcon r5.11H processors. Experimental phenotype probability vectors are constructed from tallying classical measurements from 1000 shots for each genotype. The 11-qubit trial is conducted on a Qiskit Aer simulator instead of an experimental quantum computer, using the *ibm_brisbane* noise profile to simulate noise. The circuits from our experimental trials are depicted in the SM [42].

## IV. RESULTS

**FIG. 2.**
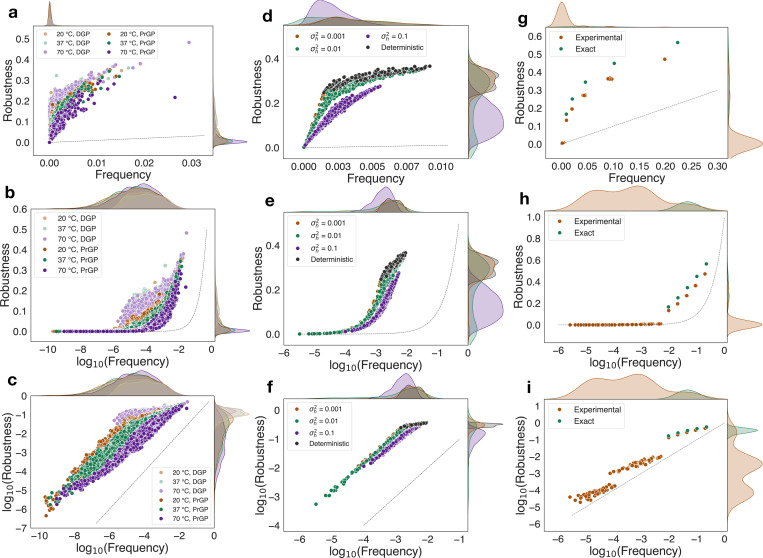
Plots of (a, d, g) robustness versus frequency, (b, e, h) robustness versus log_10_(frequency), and (c, f, i) log_10_(robustness) versus log_10_(frequency) for (a, b, c) RNA folding, (d, e, f) spin glass ground state, and (g, h, i) quantum circuit PrGP maps. The dashed line is the random null expectation $\rho_{n}=f_{n}$.

After running simulations to obtain the raw PrGP map data from the RNA, spin glass, and quantum circuit numerical experiments, we computed robustness, genotype entropy distributions, and phenotype distributions, which we plot in Figure 2, Appendix C, and Appendix E, respectively. Transition probabilities between different phenotypes and the RNA k=4, ℓ=12 genotype entropy distribution are plotted in the SM [42]. As noted previously, validation trial data for spin glasses on a different random graph as well as multiple experimental quantum circuit trials’ data are also provided in the SM [42]. The SM also contains a table in which we note the frequencies of the RNA “unfolded” phenotype; notably, in the k=2, ℓ=20 cases, the unfolded phenotype frequency is less than 3% while for k=4, ℓ=12 case, the unfolded phenotype frequency is more than 80% and there are much fewer phenotypes, as expected from the RNA12 DGP study [3].

In Figure 2 we plot robustness versus frequency, robustness versus log frequency, and log robustness versus log frequency for each of the 3 main systems studied (additional RNA, spin glass, and quantum circuit trials are in the SM [42]). Notable common features across all systems include robustness much higher than predicted by the null model for sufficiently large frequencies and a convergence toward the null model behavior for sufficiently small frequencies. The RNA PrGP maps, all show suppressed robustness relative to their DGP counterparts, and this scaling is further suppressed as temperature increases.

Similarly, in spin glasses, the DGP robustness is highest and closest to the linear-log relationship; the PrGP maps show increasingly suppressed scaling as the disorder variance is tuned higher. In quantum circuit PrGP maps, the trials with experimental or simulated noise show the appearance of a long tail of many new small-frequency phenotypes with, leading to the suppression of the robustness of the large-frequency phenotypes with a maintenance of the approximate log $f_{n}$ scaling.

From the phenotype entropy distributions in Appendix E, we see that as disorder parameters are increased (temperature, field variance, measurement noise), phenotype entropy distributions widen, meaning a genotype is more likely to have a broader distribution of phenotypes to which it maps.

**FIG. 3.**
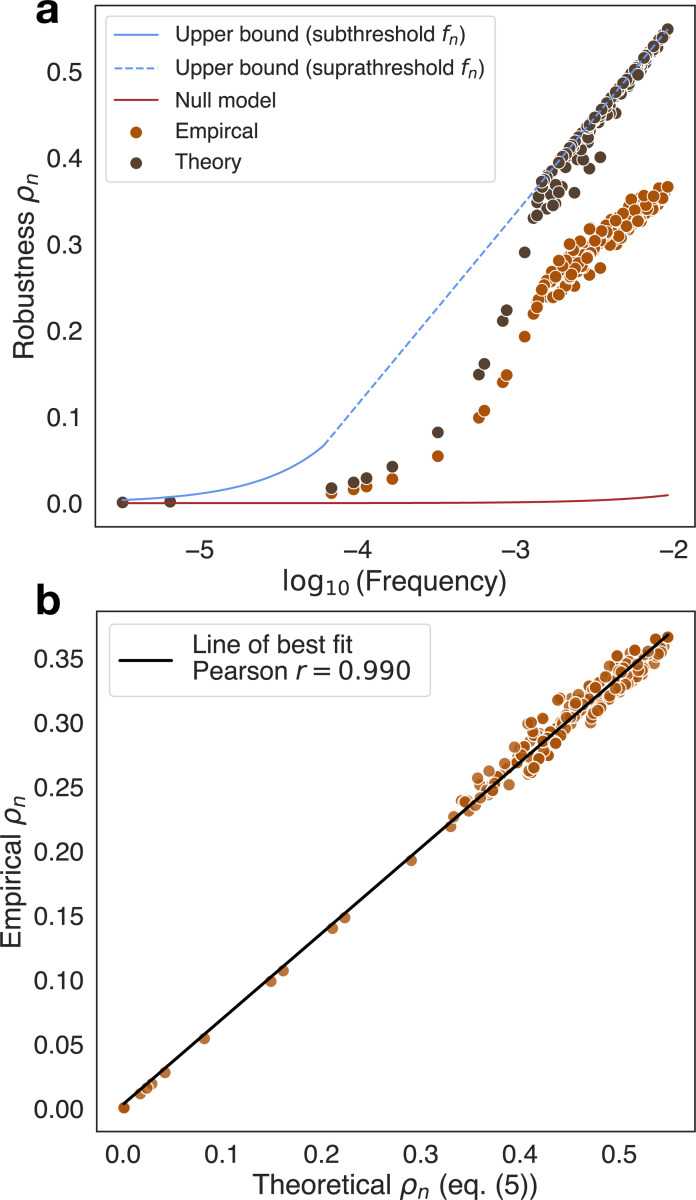
(a) Plot of log_10_(frequency) versus robustness $\rho_{n}$, where $\rho_{n}$ has either been computed empirically from the experimental data or theoretically from eq. (5) for the spin glass system ($\sigma_{h}^{2}=0.001$). Includes upper bounds from eq. (6) and null model. (b) Scatter plot of theoretical $\rho_{n}$ versus empirical $\rho_{n}$ for the spin glass system ($\sigma_{h}^{2}=0.001$) with Pearson r=0.990.

We now make predictions of robustness by directly plugging in measurements of $S_{n}^{\gamma }$ and $f_{n}$ into eq. (5). We show an example plot of the theoretical robustness, empirical robustness, null model, and upper bound for spin glasses with $\sigma_{h}^{2}=0.001$ in Figure 3(a). Not only does the the theoretical robustness, given only $S_{n}^{\gamma }$ and $f_{n}$, recapitulate the salient scaling behavior of the empirical robustness, as shown in Figure 3(b), but the Pearson correlation between the predicted and empirical robustness is r=0.990; in Table II, we show that the Pearson correlations from robustness obtained from eq. (5) for all systems tabulated range from 0.947–0.99994 and outperformed the null model and DGP maximum robustness formulas across all systems, illustrating the success of eq. (5). While the Pearson correlations are high, the prediction from eq. (5) varies by additive or multiplicative constant factors likely due to violation of one or both assumptions mentioned in the Theory section. As disorder parameters increase, these violations become more prominent and eq. (5) and the null model’s relative performance becomes better (see Table II), meaning that biphasic scaling starts to fade away in favor of null model-like linear scaling when there is too much disorder. Nonetheless, in all cases our analytical theory performs the best and remains highly predictive.

We now combine empirical results for $S_{n}^{\gamma }$ versus $f_{n}$ with eq. (5) to develop a semi-empirical theory for understanding how robustness $\rho_{n}$ scales versus $f_{n}$. We observe in Appendix C that genotype entropy, which is exactly $S_{n}^{\gamma }=\ell \text{log}k+\text{log}f_{n}$ for DGP maps, empirically maintains similar scaling

(7) $$S_{n}^{\gamma }\approx \alpha +\eta \text{log}f_{n}$$

for some α and η in PrGP maps, but is generally increased with respect to the DGP genotype entropy and generally with 0≤η≤1. This means that a phenotype with fixed frequency is likely to be spread out over more genotypes in the PrGP case than in the DGP case, as expected. This relationship tends to hold over many orders of magnitude, for all 3 systems, though with slightly differing behavior. For instance, there are some cases where η depends on $f_{n}$, but is constant for large stretches of frequencies. One example is spin glasses with $\sigma_{h}^{2}=0.001$, where η≈1 for most common phenotypes and then suddenly transitions to η≈0 for sufficiently small frequencies. Regardless, eq. (7) can still be combined with eq. (5) to understand how $\rho_{n}$ scales with $f_{n}$, and different values of η can be used in limits of small or $f_{n}$.

Substituting in eq. (7) into eq. (5), we have the robustness expression

(8) $$\rho_{n}=\frac{k^{\ell }}{e^{\alpha }\ell \text{log}k}\left[f^{1-\eta }\left(\alpha +\eta \text{log}f_{n}\right)\right],$$

Notably, when η=0 (e.g. for sufficiently small frequencies in the spin glass $\sigma_{h}^{2}=0.001$), eq. (8) becomes $\rho_{n}∼f_{n}$. In RNA, quantum circuits, and the spin glass $\sigma_{h}^{2}=0.1$ case, a slope 0<η<1 is observed. In these cases, we can see from the formula above that $\rho_{n}\to -\infty$ when $f_{n}\to 0$. However, the appropriate limit should actually take into account the fact that the smallest phenotype frequency, $f_{\min}$, is finite. We show in Appendix D that when $f_{n}\geq f_{\min}\gg e^{-\alpha /\eta }$, which is the case for the empirical systems in which 0<η<1, then a power law relationship $\text{log}\rho_{n}∼C+(1-\eta )\text{log}f_{n}$ is expected for many orders of magnitude of frequency. Only after frequencies are so small that $f_{\min}\gg e^{-\alpha /\eta }$ is violated would a sharp divergence to −∞ occur, but empirical frequencies observed in this study do not reach this regime. In Figure 2, we indeed observe a clear power law relationship for sufficiently small frequencies, which of course simplifies to the aforementioned linear relationship when η=0. When η is small but not 0, it may be difficult to distinguish a power law from a linear relationship from Figure 2.

Lastly, for sufficiently large frequencies, for 0<η<1, we generally have a complex behavior $\rho ∼f^{1-\eta }\left(\alpha +\eta \text{log}f_{n}\right)$ which can be expanded to leading order in $f_{n}$ or to leading order in $\text{log}f_{n}$, depending on the variable choice. For example, substituting $x_{n}=\text{log}f_{n}$ into eq. (8), the leading order behavior for small $x_{n}$ becomes $\rho_{n}∼a+b\text{log}f_{n}$, which is the expected large frequency behavior. It is important to note that, for examples such as the aforementioned spin glasses with $\sigma_{h}^{2}=0.001$ and even $\sigma_{h}^{2}=0.01$, η≈1 yields the “robust” logarithmic scaling seen in robust DGP maps and the PrGP upper bound computed here. Moreover, in the RNA systems, for sufficiently high frequencies the $S_{n}^{\gamma }$ versus $f_{n}$ plot points reach the exact DGP $S_{n}^{\gamma }$ curve, which has η=1. In quantum circuits, the general trend of $S_{n}^{\gamma }$ is to lay parallel or almost parallel to the DGP $S_{n}^{\gamma }$ curve, which also suggests η≈1 or slightly less than 1. However, there are clusters of phenotypes with 0<η<1, which would each have different estimated α values. This leads to a fragmented genotype entropy curve and robustness curve which may not be entirely explained by the monotonic behavior predicted by eq. (8), though combining empirical measurements in eq. (5) still yields excellent predictions. Nonetheless, different regimes of eq. (8) provide evidence for the complex behavior seen in PrGP map robustness.

## V. DISCUSSION

Compared to existing DGP maps, PrGP maps not only allow for the inclusion of realistic, physical sources of disorder like thermal fluctuation and external variables, but they also permit the analysis of new systems like quantum circuits with inherent uncertainty. We emphasize the broad applicability of this framework to a vast array of systems across biology, physics, and computer science, and other disciplines for the analysis of robustness and stability. The analytical theory introduced here, which functions well outside of the approximation regimes used to derive it, provides a link between a phenotype’s frequency $f_{n}$, the genotype entropy $S_{n}^{\gamma }$, and the robustness $\rho_{n}$. Given the empirical observation of a logarithmic relationship between $S_{n}^{\gamma }$ and $f_{n}$, we can show that for high frequencies a complex $\rho ∼f^{1-\eta }\left(\alpha +\eta \text{log}f_{n}\right)$ robustness relationship, which becomes linear-log (DGPlike) robustness when η=1 is obtained, while for small frequencies linear or power law relationship is expected, depending on system specific information. Moreover, as disorder in a system is increased, phenotypes spread over a larger number of genotypes, leading to increasingly suppressed robustness and more null model-like behavior. Most notably, our theory in eq. (5) is highly successful, measured by Pearson correlation, in predicting empirical robustness across all systems.

The scaling we observe empirically and justify theoretically in this article is observed in all three studied systems, despite being disparate, hinting at its universality. How this robustness trend affects navigability of (probabilistic) fitness landscapes is an important direction for further investigation. We suggest that evolutionary dynamics on fitness landscapes where the genotype-to-phenotype mapping is probabilistic may display unique phenomena which are not present on fitness landscapes with purely deterministic GP mapping.

We also suggest that the mapping of genotypes to probability vectors instead of discrete phenotypes may facilitate the taking of gradients of, for instance, a negative loss-likelihood loss function in the process of learning PrGP or even DGP maps using statistical learning methods. Specifically, one might model a GP map using a graph neural network [46] and predict the phenotype or related properties of neighboring nodes. Such a model may ultimately aid in inferring fitness landscapes from limited initial GP data [47–49].

## Supplementary Material

Supplement 1

## Figures and Tables

**TABLE I.** Overview of the genotypes and phenotypes of each PrGP system, as well as their respective sources of uncertainty.

System	Genotype Alphabet	Alphabet size k	Phenotype	Source of Uncertainty

RNA folding	{A,U,G,C} (or {G,C})	4 (or 2)	Folded dot-bracket structure	Thermal fluctuation, T>0
Spin glass ground state	{−1,+1}	2	Ground state spin configuration	Disordered external field
Quantum circuit	$\left\{\text{Z},\text{X},\text{Y},\text{H},\text{S},{\text{S}}^{†},\text{T},{\text{T}}^{†}\right\}$	8	Classical measurement of circuit output	Superposition and entanglement

**TABLE II.** Pearson correlation coefficient r between robustness predicted from eq. (5) versus empirically measured robustness. In general, the theory outperforms the null model and the DGP maximum approximations, and overall Pearson correlations are very high, close to 1, highlighting the success of eq. (5). Bold indicates the best-performing model.

System	Details	Theory vs. Real Pearson r	Null vs. Real Pearson r	DGP Max vs. Real Pearson r
Spin Glass	$\sigma_{h}^{2}=0.001$	**0.990**	0.766	0.940
Spin Glass	$\sigma_{h}^{2}=0.01$	**0.994**	0.874	0.924
Spin Glass	$\sigma_{h}^{2}=0.1$	**0.995**	0.976	0.954
RNA GC20	20°C	**0.947**	0.811	0.797
RNA GC20	37°C	**0.951**	0.854	0.766
RNA GC20	70°C	**0.965**	0.856	0.665
RNA12	37°C	**0.958**	0.832	0.882
Quantum Circuit	11 qubit (exact)	**0.99994**	0.901	0.997
Quantum Circuit	11 qubit (simulation)	**0.9991**	0.865	0.576
Quantum Circuit	7 qubit, trial 1 (exact)	**0.99994**	0.926	0.998
Quantum Circuit	7 qubit, trail 1 (exp.)	**0.9996**	0.912	0.712
