# Supplementary material for: Probabilistic Genotype-Phenotype Maps Reveal Mutational Robustness of RNA Folding, Spin Glasses, and Quantum Circuits
Source: ArXiv. 2025 Jan 3:arXiv:2301.01847v3. Originally published 2023 Jan 4. Preprint. [Version 3] (PMC9882568)
Supplement: Supplement 1 [file NIHPP2301.01847v3-supplement-1.pdf]

I. EXTENDED DATA FOR MAIN TEXT RNA FOLDING PrGP MAP, GC ALPHABET, $\ell = 20, k = 2$

In the main text, we presented robustness versus frequency plots with linear and log scaling for RNA folding PrGP and DGP maps at three temperatures. For clarity, we have included robustness versus frequency, robustness versus $\log_{10}$(frequency), and $\log_{10}$(robustness) versus $\log_{10}$(frequency) plots separately for PrGP and DGP maps in Figure S1. First, we see that the DGP map results reproduce the expected $\rho_n \propto \log f_n$ relationship for most phenotypes, with significant elevation above the random null model expectation. We also note that there is little temperature dependence in DGP robustness calculations, which suggests the effect of temperature does little to alter the exact ground state phenotype. In contrast, our PrGP map results showcase a different robustness behavior in which as simulation temperature increases, there is a gradual but clear suppression of the robustness versus frequency relationship; see main text for discussion of these features. In the PrGP map results we also note a biphasic behavior in which for high frequency phenotypes, the PrGP map robustness, similar to the DGP map robustness, is substantially elevated above the random null expectation and for lower frequencies, the robustness behaves more like the random model.

In Table S1, we include the Pearson correlation coefficient r and Spearman rank correlation coefficient ρ for each map (PrGP, DGP), temperature (20 °C, 37 °C, 70 °C), and axis transformation presented in Figure 2(a-b) and Figure S1. The primary feature we point out is the relative decrease of the PrGP Pearson r coefficients in robustness versus $\log_{10}$(frequency) plots as compared to the DGP plots; this suggests a deviation from the empirical $\rho_n \propto \log f_n$ trend observed in DGP studies.

In the GP map literature, *phenotype bias*, the finding that phenotype frequencies can vary over many orders of magnitude with a small number of phenotypes being the targets of a large number of genotypes, has been shown for many systems [1–4]. In Figure S2, we present plots of $\log_{10}$(frequency) versus normalized rank and $\log_{10}$(frequency) versus $\log_{10}$(normalized rank) for each temperature and map pairing which show phenotype bias for this RNA folding system. Notably, the $\log_{10}$(frequency) versus $\log_{10}$(normalized rank) plot suggests a deviation from Zipf's law.

Figure S3 presents transition probabilities ϕ_{mn} for the most frequently occurring phenotype n to the other phenotypes m due to a single nucleotide mutation for both PrGP and DGP maps at three different temperatures. For each respective map, a plot including and excluding the most robust transition (*i.e.* from phenotype $n \to n$) is shown for added clarity. This figure demonstrates that the off-diagonal transition probabilities for PrGP maps maintained an approximate relationship $\phi_{mn} \propto f_m$ for $m \neq n$ in concordance with DGP maps, and in concordance with the random null expectation for PrGP maps (see main text). A proportionality constant not equal to 1 for $\phi_{mn} \propto f_m$ with $m \neq n$ is likely due to transition probability mass that is acquired by the diagonal element ϕ_{nn}. It is also apparent that the most robust transition is much more likely than the transition to any other phenotype, in support of our claim that PrGP maps, like DGP maps, exhibit enhanced robustness.

System	Alphabet, Length	Map	Temperature	Axes	Pearson r	Spearman ρ
RNA	GC, 20	PrGP	20 °C	Robust v. Freq	0.811	0.962
RNA	GC, 20	PrGP	20 °C	Robust v. $\log_{10}$(Freq)	0.813	0.962
RNA	GC, 20	PrGP	20 °C	$\log_{10}$(Robust) v. $\log_{10}$(Freq)	0.951	0.962
RNA	GC, 20	PrGP	37 °C	Robust v. Freq	0.854	0.974
RNA	GC, 20	PrGP	37 °C	Robust v. $\log_{10}$(Freq)	0.784	0.974
RNA	GC, 20	PrGP	37 °C	$\log_{10}$(Robust) v. $\log_{10}$(Freq)	0.970	0.974
RNA	GC, 20	PrGP	70 °C	Robust v. Freq	0.856	0.982
RNA	GC, 20	PrGP	70 °C	Robust v. $\log_{10}$(Freq)	0.665	0.982
RNA	GC, 20	PrGP	70 °C	$\log_{10}$(Robust) v. $\log_{10}$(Freq)	0.982	0.982
RNA	GC, 20	DGP	20 °C	Robust v. Freq	0.721	0.860
RNA	GC, 20	DGP	20 °C	Robust v. $\log_{10}$(Freq)	0.868	0.860
RNA	GC, 20	DGP	20 °C	$\log_{10}$(Robust) v. $\log_{10}$(Freq)	0.839	0.860
RNA	GC, 20	DGP	37 °C	Robust v. Freq	0.717	0.856
RNA	GC, 20	DGP	37 °C	Robust v. $\log_{10}$(Freq)	0.859	0.856
RNA	GC, 20	DGP	37 °C	$\log_{10}$(Robust) v. $\log_{10}$(Freq)	0.836	0.856
RNA	GC, 20	DGP	70 °C	Robust v. Freq	0.759	0.914
RNA	GC, 20	DGP	70 °C	Robust v. $\log_{10}$(Freq)	0.903	0.914
RNA	GC, 20	DGP	70 °C	$\log_{10}$(Robust) v. $\log_{10}$(Freq)	0.884	0.914

TABLE S1. Pearson and Spearman correlation coefficients for all robustness versus frequency plots in main text/Supplemental Material for RNA $k = 2, \ell = 20$ simulations with reduced alphabet, for each simulation temperature.

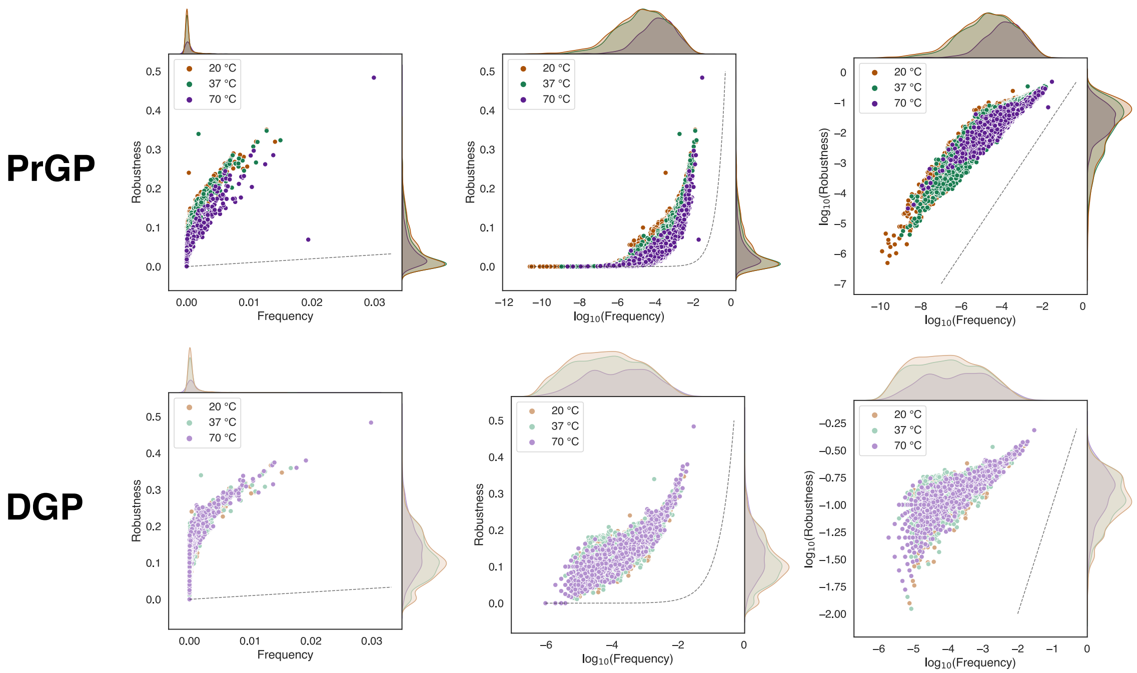

FIG. S1. Plots of (left) robustness versus frequency, (middle) robustness versus $\log_{10}$(frequency), and (right) $\log_{10}$(robustness) versus $\log_{10}$(frequency) for RNA folding (top row) PrGP maps and (bottom row) DGP maps for three temperatures. These data are the same results from main text Figure 2, with axis scaling adjusted and with PrGP and DGP data shown separately for clarity. The dashed line is the random null expectation for both PrGP and DGP maps given by $\phi_{mn} = f_m$ for all m and n.

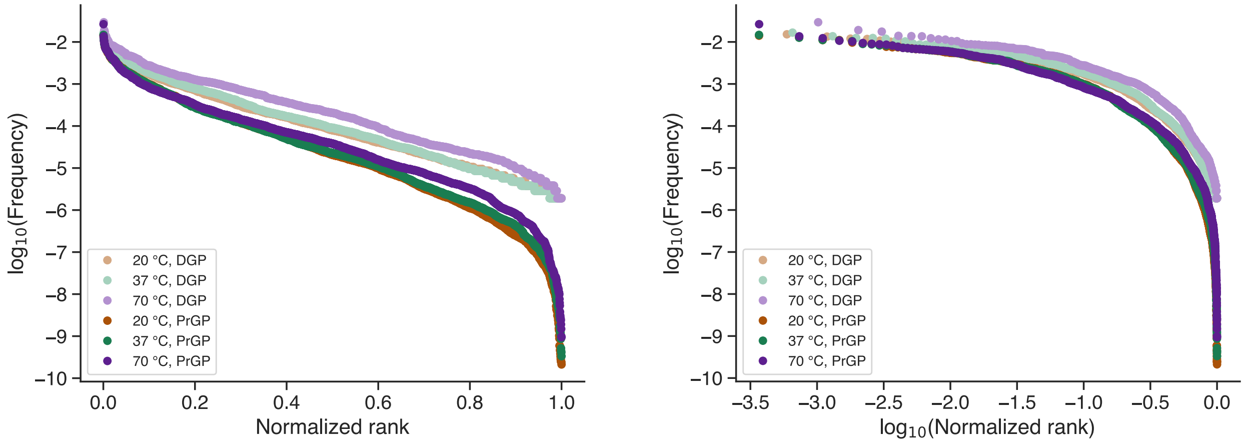

FIG. S2. Plots of (left) $\log_{10}$(frequency) versus normalized rank and (right) $\log_{10}$(frequency) versus $\log_{10}$(normalized rank) for RNA folding PrGP and DGP maps for three temperatures. When computing ranks, ties were broken arbitrarily.

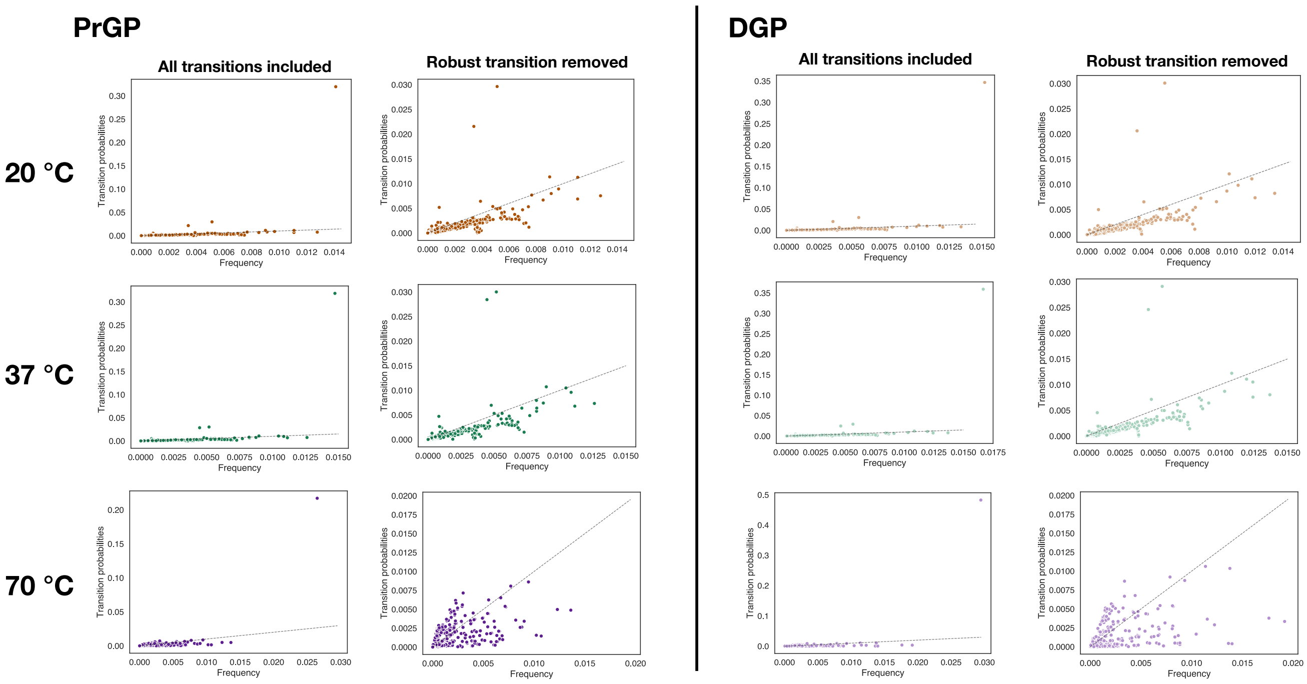

FIG. S3. Plots of transition probabilities versus frequency for RNA folding (left) PrGP maps and (right) DGP maps for three temperatures, (top) 20° C, (middle) 37° C, and (bottom) 70° C. For each respective map, plots include either (left) all transitions or (right) have the most robust transition removed. The dashed line is the random null expectation for both PrGP and DGP maps given by $\phi_{mn} = f_m$ for all m and n.

II. VALIDATION TRIAL FOR RNA FOLDING PrGP MAP, FULL ALPHABET, $\ell = 12, k = 4$

Here, we present results of a validation trial for RNA folding PrGP maps for sequences of length $\ell = 12$ utilizing the full alphabet of size $k = 4$, $\{A, C, G, U\}$. In Figure S4, we present robustness versus frequency, robustness versus $\log_{10}$(frequency), and $\log_{10}$(robustness) versus $\log_{10}$(frequency) plots for RNA folding for PrGP and DGP maps. As with the reduced alphabet case, we see both PrGP and DGP map results show significant elevation above the random null model expectation, with PrGP map results demonstrating a gradual but clear suppression of the robustness versus frequency relationship compared to DGP map results. The expected $\rho_n \propto \log f_n$ relationship for phenotypes in the DGP map results as well as the biphasic behavior of the PrGP map results is present but less clear in this case, likely due to a small size effect from the limited number of phenotypes present in this complete alphabet ($k = 4$, $\ell = 12$) system compared to the reduced alphabet system ($k = 2$), which contains sequences of longer length ($\ell = 20$). Also in Figure S4, we plot the distribution of phenotype entropy $S(g)$ across all genotypes g; most phenotype entropies are zero due to their being deterministic because for the RNA folding, $k = 4$, $\ell = 12$ system most genotypes do not fold.

In Table S2, we include the Pearson correlation coefficient r and Spearman rank correlation coefficient ρ for each map (PrGP, DGP) and axis transformation presented in Figure S4. In Figure S5, we present plots of $\log_{10}$(frequency) versus normalized rank and $\log_{10}$(frequency) versus $\log_{10}$(normalized rank) for each temperature and map pairing which show phenotype bias for this RNA folding system. Notably, the $\log_{10}$(frequency) versus $\log_{10}$(normalized rank) plot suggests a deviation from Zipf's law.

Figure S6 presents transition probabilities ϕ_{mn} for the most frequently occurring phenotype n to the other phenotypes m due to a single nucleotide mutation for both PrGP and DGP maps. For each respective map, a plot including and excluding the most robust transition is shown for added clarity. This figure demonstrates that the off-diagonal transition probabilities for PrGP maps maintained an approximate relationship $\phi_{mn} \propto f_m$ for $m \neq n$ in concordance with DGP maps, and in concordance with the random null expectation for PrGP maps (see main text). A proportionality constant not equal to 1 for $\phi_{mn} \propto f_m$ with $m \neq n$ is likely due to transition probability mass that is acquired by the diagonal element ϕ_{nn}. It is also apparent that the most robust transition is much more likely than the transition to any other phenotype, in support of our claim that PrGP maps, like DGP maps, exhibit enhanced robustness.

**System**	**Alphabet, Length**	**Map**	**Axes**	**Pearson** r	**Spearman** ρ
RNA	AUCG, 12	PrGP	Robust v. Freq	0.832	0.927
RNA	AUCG, 12	PrGP	Robust v. $\log_{10}$(Freq)	0.882	0.927
RNA	AUCG, 12	PrGP	$\log_{10}$(Robust) v. $\log_{10}$(Freq)	0.877	0.927
RNA	AUCG, 12	DGP	Robust v. Freq	0.788	0.244
RNA	AUCG, 12	DGP	Robust v. $\log_{10}$(Freq)	0.814	0.244
RNA	AUCG, 12	DGP	$\log_{10}$(Robust) v. $\log_{10}$(Freq)	0.855	0.244

TABLE S2. Pearson and Spearman correlation coefficients for all robustness versus frequency plots for RNA $k = 4, \ell = 12$ validation trial with reduced alphabet. Simulations were conducted at 37 °C.

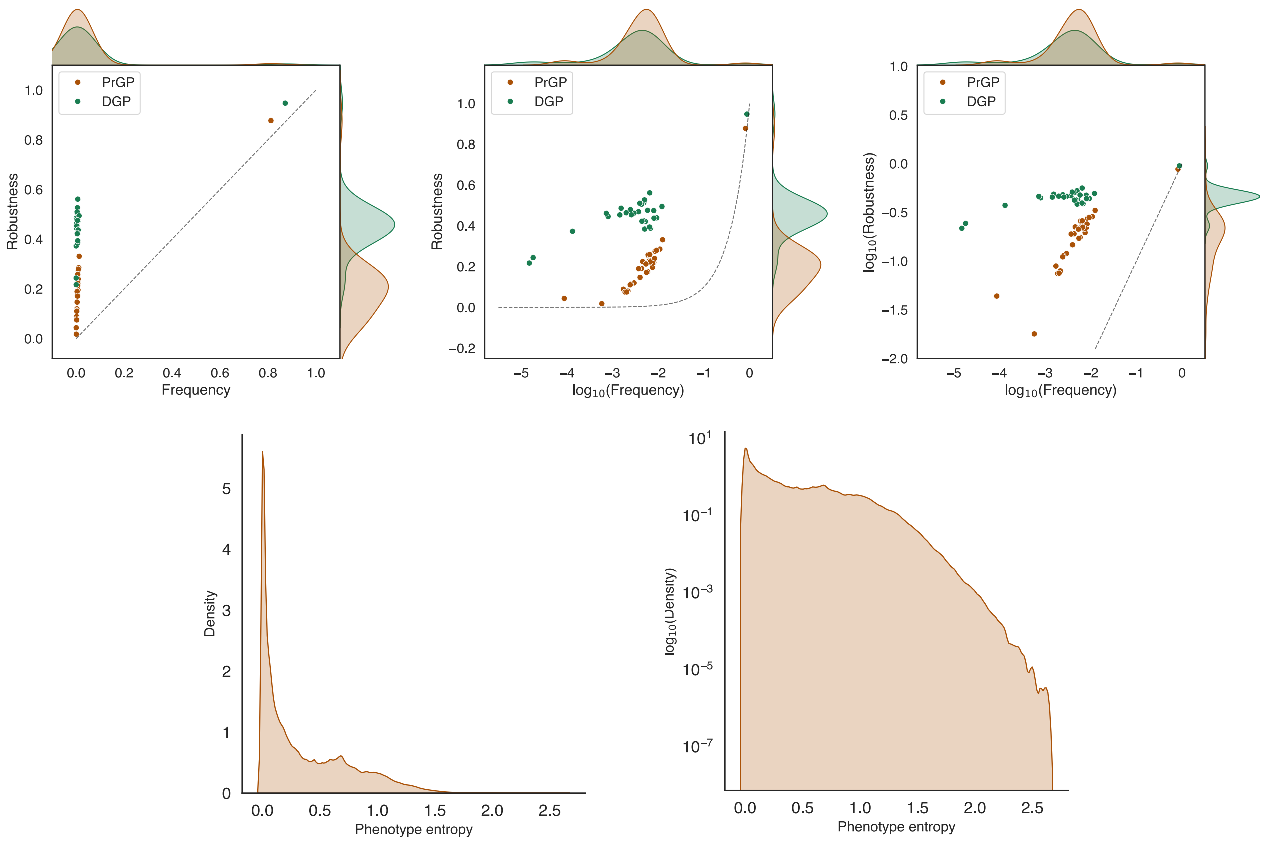

FIG. S4. Plots of (top left) robustness versus frequency, (top middle) robustness versus $\log_{10}$(frequency), and (top right) $\log_{10}$(robustness) versus $\log_{10}$(frequency) for RNA folding PrGP and DGP maps. Plots of (bottom left) density versus phenotype entropy and (bottom right) $\log_{10}$(density) versus phenotype entropy. The dashed line is the random null expectation for both PrGP and DGP maps given by $\phi_{mn} = f_m$ for all m and n.

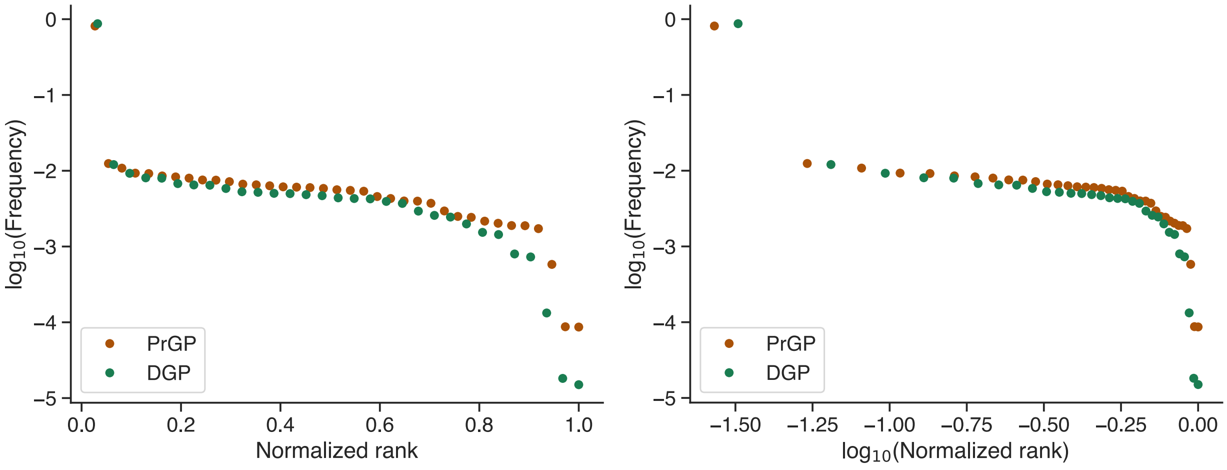

FIG. S5. Plots of (left) $\log_{10}$(frequency) versus normalized rank and (right) $\log_{10}$(frequency) versus $\log_{10}$(normalized frequency) for RNA folding PrGP and DGP maps. When computing ranks, ties were broken arbitrarily.

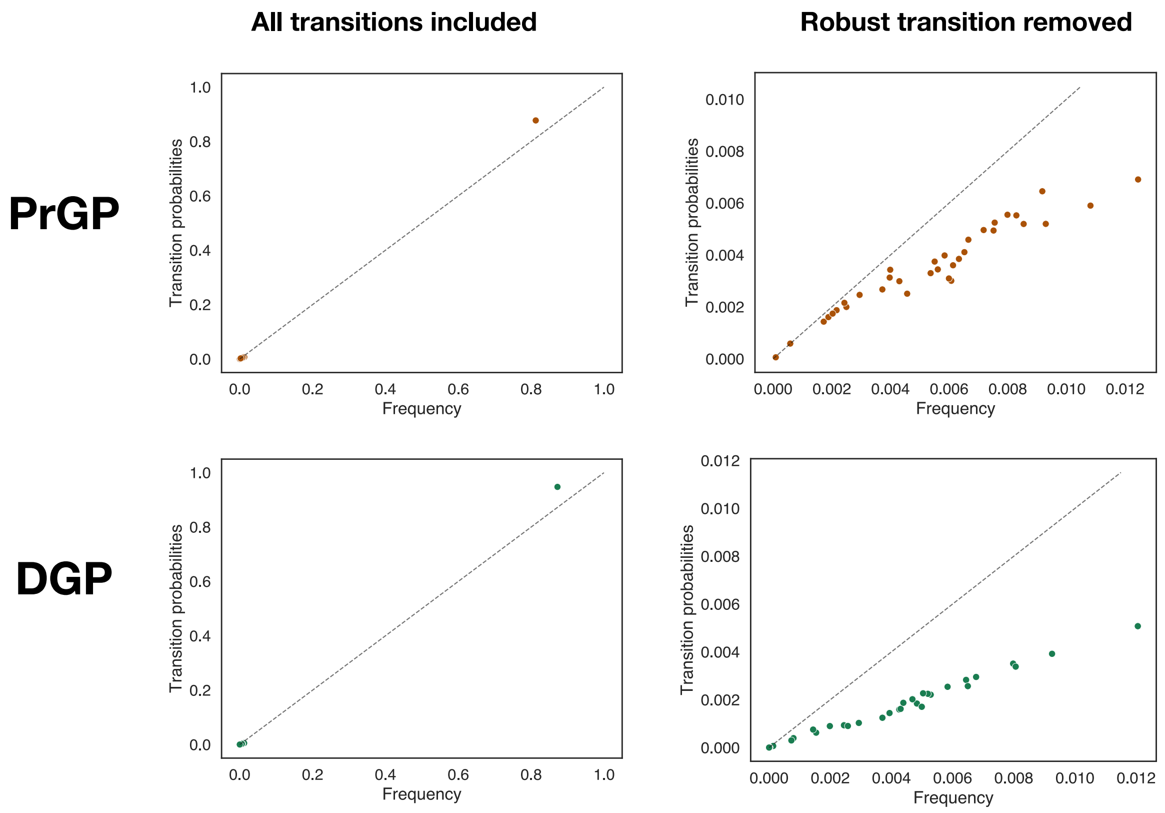

FIG. S6. Plots of transition probabilities versus frequency RNA folding (top) PrGP maps and (bottom) DGP maps. For each respective map, plots include either (left) all transitions or (right) have the most robust transition removed. The dashed line is the random null expectation for both PrGP and DGP maps given by $\phi_{mn} = f_m$ for all m and n.

A. Genotype Entropy for $k = 4, \ell = 12$ Case

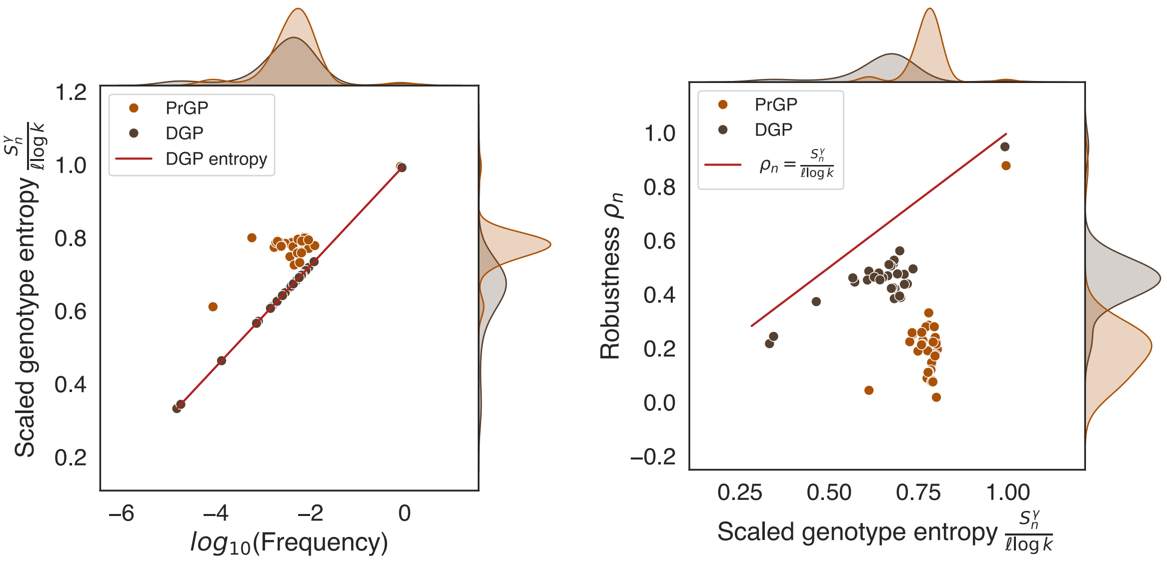

FIG. S7. Plots of (left) $\log_{10}$(frequency) versus scaled genotype entropy $\frac{S_n^\gamma}{\ell \log k}$ and (right) scaled genotype entropy $\frac{S_n^\gamma}{\ell \log k}$ versus robustness ρ_n for RNA folding PrGP maps with $k = 4, \ell = 12$.

III. FREQUENCY OF UNFOLDED PHENOTYPES FOR ALL RNA STUDIES

**System**	**Details**	**DGP Map Frequency f_n**	**PrGP Map Frequency f_n**
RNA GC20	20 °C	0.000347	0.000650
RNA GC20	37 °C	0.00147	0.00187
RNA GC20	70 °C	0.0294	0.0265
RNA12	37 °C	0.872	0.812

TABLE S3. Frequency of unfolded phenotypes for each RNA case, both for the DGP map and PrGP map.

We note that in all of the GC20 cases, the unfolded phenotype has very low frequency, meaning that the vast majority of phenotypes fold, even in the deterministic cases. In the RNA12 case, which has fewer phenotypes, the vast majority of sequences do not fold. However, the robustness curves remain qualitatively similar to the GC20 cases, and the topological properties of genotype networks formed in the RNA12 DGP map [5] are consistent with those of other DGP maps [4].

IV. EXTENDED DATA FOR MAIN TEXT SPIN GLASS PrGP MAP

In the main text, we compared a spin glass DGP map with a fixed random external field $\{h_{0,i}\}$ with our spin glass PrGP map, which introduces a Gaussian distribution to the external field whose means are fixed at $\{h_{0,i}\}$ and whose variance σ_h^2 is varied as an independent variable. Figure S8 shows the topology of the graph $\mathcal{G}(V, E)$ (with $|V| = 9$, $|E| = 15$) that corresponds to the spin glass PrGP map data presented in the main text.

In Table S4, we include the Pearson correlation coefficient r and Spearman rank correlation coefficient ρ for each map (PrGP, DGP), external field variance ($\sigma_h^2 = 0.001$, $\sigma_h^2 = 0.01$, $\sigma_h^2 = 0.1$), and axis transformation presented in Figure 2. The primary feature we point out is the relative decrease of the PrGP Pearson r coefficients in robustness versus $\log_{10}$(frequency) plots as compared to the DGP (deterministic) plot; this suggests a deviation from the empirical $\rho_n \propto \log f_n$ trend observed in the spin glass DGP study [6].

In Figure S9, we present plots of $\log_{10}$(frequency) versus normalized rank and $\log_{10}$(frequency) versus $\log_{10}$(normalized rank) for each external field variance and the deterministic case. Notably, the $\log_{10}$(frequency) versus $\log_{10}$(normalized rank) plot suggests a deviation from Zipf's law.

Figure S10 presents transition probabilities ϕ_{mn} for the most frequently occurring ground state n to the other ground states m due to a single bond perturbation. For each setting of external random field variance, a plot including and excluding the most robust transition is shown for added clarity. This figure demonstrates that the off-diagonal transition probabilities for PrGP maps maintained an approximate relationship $\phi_{mn} \propto f_m$ for $m \neq n$ in concordance with DGP maps, and in concordance with the random null expectation for PrGP maps (see main text). A proportionality constant not equal to 1 for $\phi_{mn} \propto f_m$ with $m \neq n$ is likely due to transition probability mass that is acquired by the diagonal element ϕ_{nn}. It is also apparent that the most robust transition is much more likely than the transition to any other phenotype, in support of our claim that PrGP maps, like DGP maps, exhibit enhanced robustness.

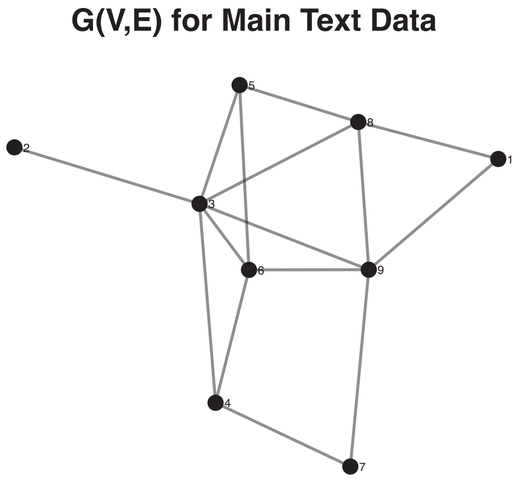

FIG. S8. Graph $\mathcal{G}(V, E)$ corresponding to the spin glass PrGP map data presented in the main text.

System	Map	σ_h^2	Axes	Pearson r	Spearman ρ
Spin glass	PrGP	0.001	Robust v. Freq	0.766	0.962
Spin glass	PrGP	0.001	Robust v. $\log_{10}$(Freq)	0.940	0.962
Spin glass	PrGP	0.001	$\log_{10}$(Robust) v. $\log_{10}$(Freq)	0.920	0.962
Spin glass	PrGP	0.01	Robust v. Freq	0.874	0.985
Spin glass	PrGP	0.01	Robust v. $\log_{10}$(Freq)	0.924	0.985
Spin glass	PrGP	0.01	$\log_{10}$(Robust) v. $\log_{10}$(Freq)	0.986	0.985
Spin glass	PrGP	0.1	Robust v. Freq	0.976	0.987
Spin glass	PrGP	0.1	Robust v. $\log_{10}$(Freq)	0.954	0.987
Spin glass	PrGP	0.1	$\log_{10}$(Robust) v. $\log_{10}$(Freq)	0.989	0.987
Spin glass	DGP	Deterministic	Robust v. Freq	0.930	0.962
Spin glass	DGP	Deterministic	Robust v. $\log_{10}$(Freq)	0.964	0.962
Spin glass	DGP	Deterministic	$\log_{10}$(Robust) v. $\log_{10}$(Freq)	0.962	0.962

TABLE S4. Pearson and Spearman correlation coefficients for all robustness versus frequency plots for the spin glass PrGP map with $|V| = 9$ and $|E| = 15$ whose data are shown in the main text and here in the Supplemental Material.

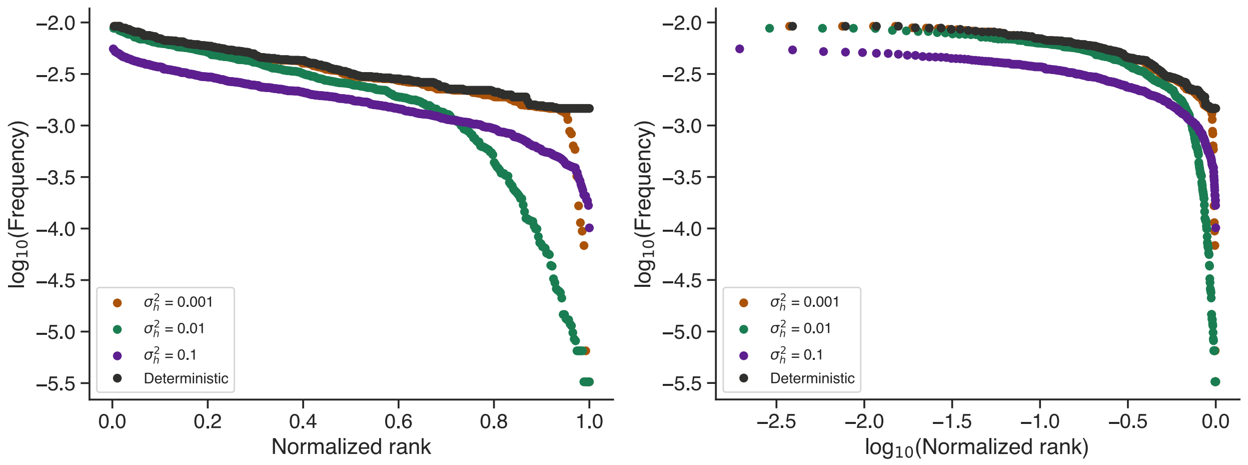

FIG. S9. Plots of (left) $\log_{10}$(frequency) versus normalized rank and (right) $\log_{10}$(frequency) versus $\log_{10}$(normalized frequency) for spin glass ground states for PrGP maps at three external field variances and DGP maps the deterministic case. When computing ranks, ties were broken arbitrarily.

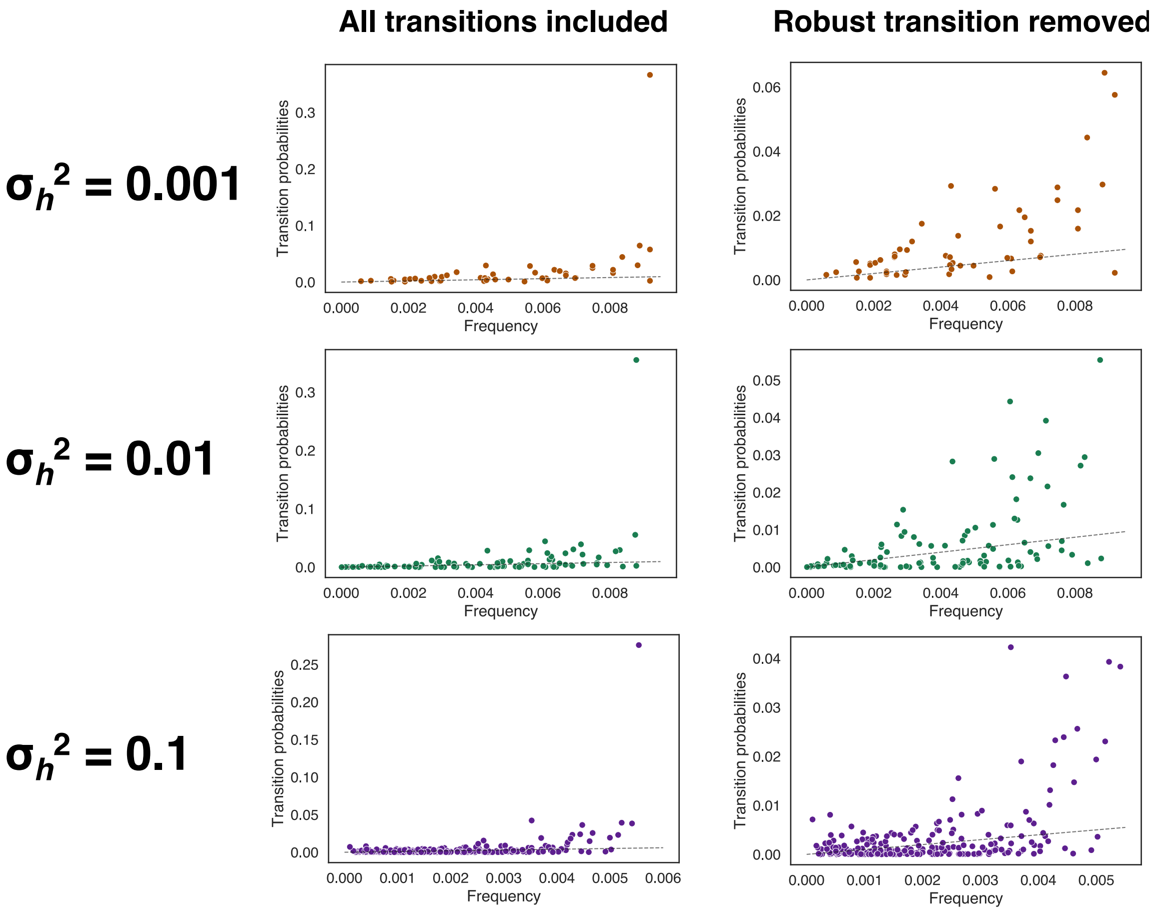

FIG. S10. Plots of transition probabilities versus frequency for spin glass ground states for PrGP maps at three external field variances, (top) $\sigma_h^2 = 0.001$, (middle) $\sigma_h^2 = 0.01$, and (bottom) $\sigma_h^2 = 0.1$. For each, plots include either (left) all transitions or (right) have the most robust transition removed. The dashed line is the random null expectation for both PrGP and DGP maps given by $\phi_{mn} = f_m$ for all m and n.

V. VALIDATION TRIAL FOR SPIN GLASS PrGP MAP

We provide a second spin glass PrGP map trial here in the Supplemental Material to illustrate that the spin glass trends described above and in the main text hold across multiple random graph instances. We generate a new $\mathcal{G}(V, E)$, once again with $|V| = 9$, $|E| = 15$ with topology shown in Figure S11. Figure S12 presents robustness versus frequency, robustness versus $\log_{10}$(frequency), and $\log_{10}$(robustness) versus $\log_{10}$(frequency) for spin glass PrGP maps at three different external field variances and for the deterministic case for DGP maps. The results from this validation trial exhibit the same behavior as observed in the trial presented in the main text. In particular, we see that as the disorder parameter increases the uncertainty in the genotype-phenotype pairing, the robustness versus frequency relationship in PrGP maps becomes suppressed relative to the DGP map limit. Again, these spin glass results are highly suggestive of a biphasic robustness relationship where at high frequencies, ρ_n is substantially enhanced above the random null expectation and behavior close to the deterministic limit is observed. However, as is clear from the corresponding $\log_{10}$(frequency) versus $\log_{10}$(robustness) plots, nearly linear behavior is observed for the smallest frequencies with the empirical robustness nearly parallel to the random expectation, signaling $\rho_n \propto f_n$. See main text for discussion of these features. Additionally, Figure S12 plots the distribution of phenotype entropy $S(g)$ across all genotypes g for PrGP maps at each external field variance experimental value. As is the case in the main text simulation, we observe that the entropy distributions shift rightward as the disorder parameter increases.

In Table S5, we include the Pearson correlation coefficient r and Spearman rank correlation coefficient ρ for each map (PrGP, DGP), external field variance ($\sigma_h^2 = 0.001$, $\sigma_h^2 = 0.00$, $\sigma_h^2 = 0.1$), and axis transformation presented in Figure S12. The primary feature we point out is the relative decrease of the PrGP Pearson r coefficients in robustness versus $\log_{10}$(frequency) plots as compared to the DGP (deterministic) plot; this suggests a deviation from the empirical $\rho_n \propto \log f_n$ trend observed in the spin glass DGP study [6].

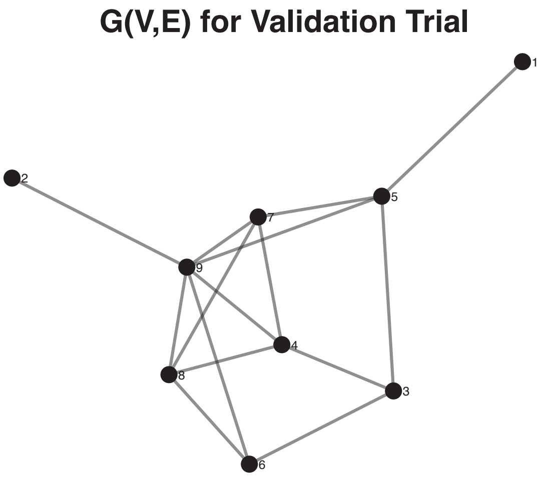

FIG. S11. Graph $\mathcal{G}(V, E)$ corresponding to the spin glass PrGP map validation trial data shown here in the Supplemental Material.

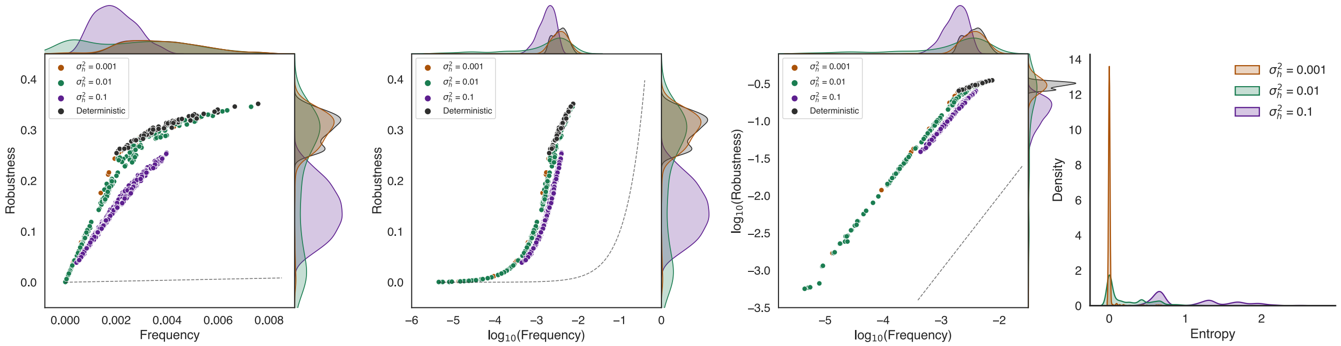

FIG. S12. Plots of (leftmost) robustness versus frequency, (middle left) robustness versus $\log_{10}$(frequency), and (middle right) $\log_{10}$(robustness) versus $\log_{10}$(frequency) for spin glass ground states for PrGP maps at three different external field variances and for the deterministic case for DGP maps. Additionally, the (rightmost) density versus phenotype entropy for the spin glass ground states at three difference external field variances is plotted. The dashed line is the random null expectation for both PrGP and DGP maps given by $\phi_{mn} = f_m$ for all m and n.

**System**	**Map**	σ_h^2	**Axes**	**Pearson** r	**Spearman** ρ
Spin glass	PrGP	0.001	Robust v. Freq	0.806	0.994
Spin glass	PrGP	0.001	Robust v. $\log_{10}$(Freq)	0.940	0.994
Spin glass	PrGP	0.001	$\log_{10}$(Robust) v. $\log_{10}$(Freq)	0.950	0.994
Spin glass	PrGP	0.01	Robust v. Freq	0.932	0.996
Spin glass	PrGP	0.01	Robust v. $\log_{10}$(Freq)	0.916	0.996
Spin glass	PrGP	0.01	$\log_{10}$(Robust) v. $\log_{10}$(Freq)	0.993	0.996
Spin glass	PrGP	0.1	Robust v. Freq	0.993	0.997
Spin glass	PrGP	0.1	Robust v. $\log_{10}$(Freq)	0.981	0.997
Spin glass	PrGP	0.1	$\log_{10}$(Robust) v. $\log_{10}$(Freq)	0.997	0.997
Spin glass	DGP	Deterministic	Robust v. Freq	0.962	0.995
Spin glass	DGP	Deterministic	Robust v. $\log_{10}$(Freq)	0.993	0.995
Spin glass	DGP	Deterministic	$\log_{10}$(Robust) v. $\log_{10}$(Freq)	0.990	0.995

TABLE S5. Pearson and Spearman correlation coefficients for all robustness versus frequency plots for the spin glass PrGP map validation trial with $|V| = 9$ and $|E| = 15$ whose data are shown above in this section.

VI. QUANTUM CIRCUIT GENERATION ALGORITHM

In this study, we generated quantum circuits with 7 qubits and 4 layers. We take the genotype of the quantum circuit PrGP map to be a subset of single qubit gates (which are varied to reflect each genotype). We first start by seeding the circuit randomly with $CNOT$ gates which cannot participate in the genotype gate list. Only certain pairs of qubits which are physically connected in the 7-qubit *ibm_lagos* v1.2.0 quantum computer can participate in the same $CNOT$ gate. The remaining open places are seeded with single qubit gates, and we choose $\ell = 4$ of these gates to be the variable gates for the genotype. The alphabet chosen is of size $k = 8$: $\{Z, X, Y, H, S, S^\dagger, T, T^\dagger\}$. Circuit diagrams used in our experimental trials are shown in the subsequent sections.

VII. EXTENDED DATA FOR MAIN TEXT QUANTUM CIRCUIT PrGP MAP

To our knowledge, this work is the first to analyze the structural properties of quantum circuit GP maps. We generate random quantum circuits as described in the main text and in the previous section with 7 qubits and 4 layers of gates. Figure S13 shows a schematic representation of the random quantum circuit generated for the quantum circuit PrGP map data presented in main text Figure 2 and in this Supplemental Material section.

In Table S6, we include the Pearson correlation coefficient r and Spearman rank correlation coefficient ρ for both exact and experimental quantum circuit PrGP results for each axis transformation presented in main text Figure 2. The primary features we point out are the high Perason correlation $r = 0.998$ of the robustness versus $\log_{10}$(frequency) relationship for the exact phenotype probability vectors, and the relative decrease of the experimental Pearson r coefficients in robustness versus $\log_{10}$(frequency) plot as compared to the exact plot. This suggests that the exact relationship exhibits behavior similar to the empirical $\rho_n \propto \log f_n$ trend observed in DGP studies, and that the experimental trials introduce measurement noise which induces a deviation from the exact results.

In Figure S14, we present plots of $\log_{10}$(frequency) versus normalized rank and of $\log_{10}$(frequency) versus $\log_{10}$(normalized rank) for experimental and exact quantum circuit PrGP map results. Notably, the plot showing $\log_{10}$(frequency) versus $\log_{10}$(normalized rank) suggests a deviation from Zipf's law.

Figure S15 presents transition probabilities ϕ_{mn} for the most frequently occurring circuit output state n to the other circuit output states m due to a single variable gate perturbation. For both experimental and exact phenotype probability vectors, a plot including and excluding the most robust transition is shown for added clarity. This figure demonstrates that the off-diagonal transition probabilities for quantum circuit PrGP maps are positively correlated with the frequency f_m, though there appears to be some additional nonrandom relationship which is not predicted from standard DGP or PrGP theory. It is also apparent that the most robust transition is much more likely than the transition to any other phenotype, in support of our claim that PrGP maps, like DGP maps, exhibit enhanced robustness.

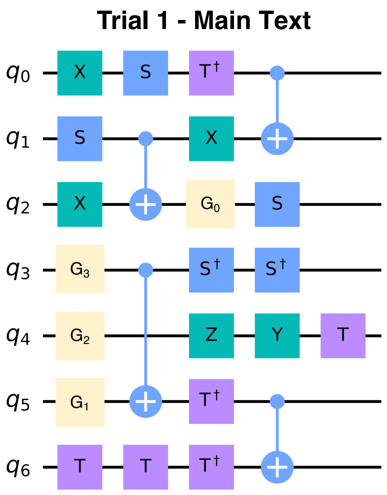

FIG. S13. Random circuit generated for quantum circuit trial 1, whose robustness data is plotted in the main text and below in the remainder of this section. The genotype is the set of variable gates $g = (G_0, G_1, G_2, G_3)$, so the length of the input sequence is $\ell = 4$ drawn from an alphabet of $k = 8$ single qubit gates: $\{Z, X, Y, H, S, S^\dagger, T, T^\dagger\}$.

System	Map	Trial	Exact or Exp	Axes	Pearson r	Spearman ρ
Quantum circuit	PrGP	1	Exact	Robust v. Freq	0.926	0.996
Quantum circuit	PrGP	1	Exact	Robust v. $\log_{10}$(Freq)	0.998	0.996
Quantum circuit	PrGP	1	Exact	$\log_{10}$(Robust) v. $\log_{10}$(Freq)	0.993	0.996
Quantum circuit	PrGP	1	Experimental	Robust v. Freq	0.912	0.987
Quantum circuit	PrGP	1	Experimental	Robust v. $\log_{10}$(Freq)	0.712	0.987
Quantum circuit	PrGP	1	Experimental	$\log_{10}$(Robust) v. $\log_{10}$(Freq)	0.983	0.987

TABLE S6. Pearson and Spearman correlation coefficients for all robustness versus frequency plots quantum circuit PrGP map whose robustness data was presented in the main text and in the above log-log plot. This includes both exact results as well as experimental results for realization/Trial 1, whose circuit is also printed earlier in this section.

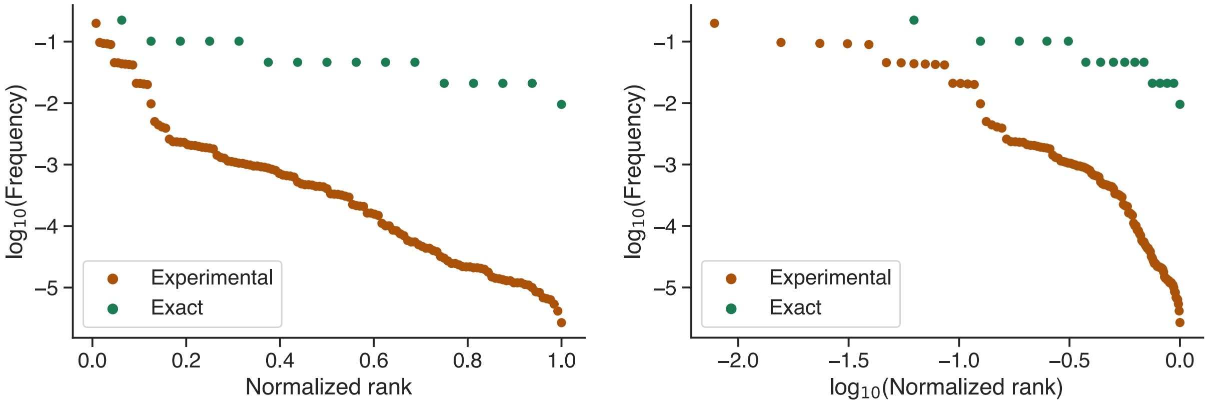

FIG. S14. Plots of (left) $\log_{10}$(frequency) versus normalized rank and (right) $\log_{10}$(frequency) versus $\log_{10}$(normalized frequency) for the quantum circuit trial 1 for experimental and exact data. The dashed line is the random null expectation for both PrGP and DGP maps given by $\phi_{mn} = f_m$ for all m and n.

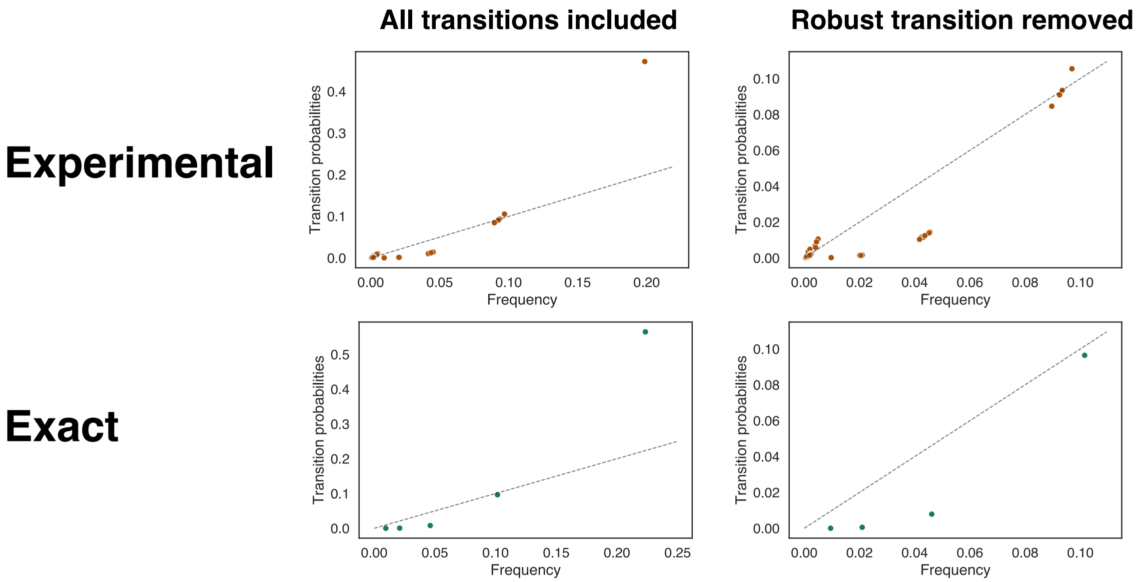

FIG. S15. Plots of (top) transition probabilities versus frequency for quantum circuit trial 1 for experimental and (bottom) exact data. For each model framework, plots include either (left) all transitions or (right) have the most robust transition removed. The dashed line is the random null expectation for both PrGP and DGP maps given by $\phi_{mn} = f_m$ for all m and n. The dashed line is the random null expectation for both PrGP and DGP maps given by $\phi_{mn} = f_m$ for all m and n.

VIII. 7 QUBIT VALIDATION TRIALS FOR QUANTUM CIRCUIT PrGP MAP

To validate the quantum circuit PrGP map results presented in the main text and Supplemental Material, six additional trials were conducted. A schematic of the random quantum circuit generated for the first of these validation trials is shown in Figure S16. Figure S17 presents robustness versus frequency, robustness versus $\log_{10}$(frequency), and $\log_{10}$(robustness) versus $\log_{10}$(frequency) for this quantum circuit PrGP map validation trial. As with the first quantum circuit PrGP map trial, these data strongly support the enhanced $\rho_n \propto \log f_n$ scaling. Again, we see the spread of phenotypes observed in the frequency domain due to superposition and/or entanglement and that many of the phentoypes are degenerate with identical frequency and robustness. This degeneracy is broken in our experimental measurements, which exhibit measurement noise. Once again, the frequency and robustness of these logarithmically scaling phenotypes is suppressed relative to the exact case as probability mass is drawn towards additional phenotypes which are observed experimentally which were not observed in the exact case. These results illustrate our suggested biphasic robustness scaling in which the low frequency phenotypes, which are introduced due to measurement noise in the experimental trials, lie much closer to the random null expectation than the higher frequency phenotypes observed in the exact calculations, which rather scale with enhanced robustness similar to what is seen in standard DGP maps. Figure S17 also presents a plot of the distribution of phenotype entropy $S(g)$ across all genotypes g for exact and experimental quantum circuit PrGP maps. Notably, the experimental entropy distribution is shifted rightward relative to the exact result due to measurement noise as well as a finite number of experimental trials.

In Table S7, we include the Pearson correlation coefficient r and Spearman rank correlation coefficient ρ for both exact and experimental quantum circuit PrGP results for each axis transformation presented in Figure S17. The primary features we point out are the high Perason correlation $r = 0.950$ of the robustness versus $\log_{10}$(frequency) relationship for the exact phenotype probability vectors, and the relative decrease of the experimental Pearson r coefficients in robustness versus $\log_{10}$(frequency) plot as compared to the exact plot. This suggests that the exact relationship exhibits behavior similar to the empirical $\rho_n \propto \log f_n$ trend observed in DGP studies, and that the experimental trials introduce measurement noise which induces a deviation from the exact results.

Figure S18 presents robustness versus frequency and robustness versus $\log_{10}$(frequency) plots as well as schematics of the corresponding random quantum circuits for validation trials 3-7. In each trial, the suggested biphasic robustness scaling is clear. Additionally, these trials support the enhanced $\rho_n \propto \log f_n$ scaling.

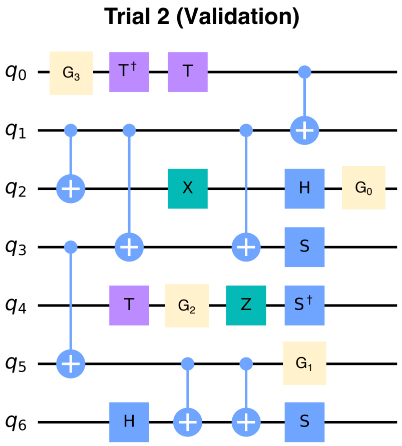

FIG. S16. Random circuit generated for quantum circuit trial 2, whose robustness and entropy data are plotted below as a validation trial. The genotype is the set of variable gates $g = (G_0, G_1, G_2, G_3)$, so the length of the input sequence is $\ell = 4$ drawn from an alphabet of $k = 8$ single qubit gates: $\{Z, X, Y, H, S, S^\dagger, T, T^\dagger\}$.

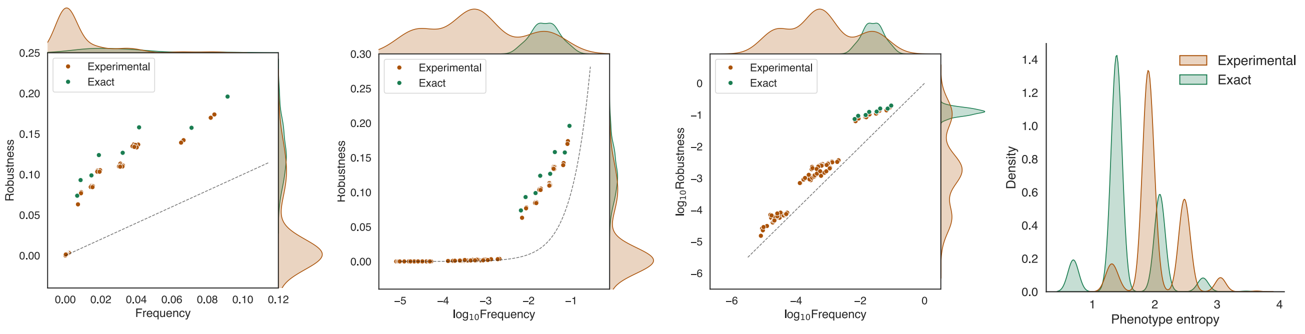

FIG. S17. Plot of (leftmost) robustness versus frequency, (left middle) robustness versus $\log_{10}$(frequency), and (middle right) $\log_{10}$(robustness) versus $\log_{10}$(frequency) for quantum circuit trial 2 for experimental and exact data. Additionally, the (rightmost) density versus phenotype entropy for quantum circuit trial 2 is plotted. The dashed line is the random null expectation for both PrGP and DGP maps given by $\phi_{mn} = f_m$ for all m and n.

**System**	**Map**	**Trial**	**Exact or Exp**	**Axes**	**Pearson** r	**Spearman** ρ
Quantum circuit	PrGP	2	Exact	Robust v. Freq	0.910	0.973
Quantum circuit	PrGP	2	Exact	Robust v. $\log_{10}$(Freq)	0.950	0.973
Quantum circuit	PrGP	2	Exact	$\log_{10}$(Robust) v. $\log_{10}$(Freq)	0.954	0.973
Quantum circuit	PrGP	2	Experimental	Robust v. Freq	0.916	0.979
Quantum circuit	PrGP	2	Experimental	Robust v. $\log_{10}$(Freq)	0.837	0.979
Quantum circuit	PrGP	2	Experimental	$\log_{10}$(Robust) v. $\log_{10}$(Freq)	0.989	0.979

TABLE S7. Pearson and Spearman correlation coefficients for all robustness versus frequency plots quantum circuit PrGP map whose robustness data is shown above as a Validation trial (i.e. Trial 2). This includes both exact results as well as experimental results for Trial 2, whose circuit is also printed earlier in this section.

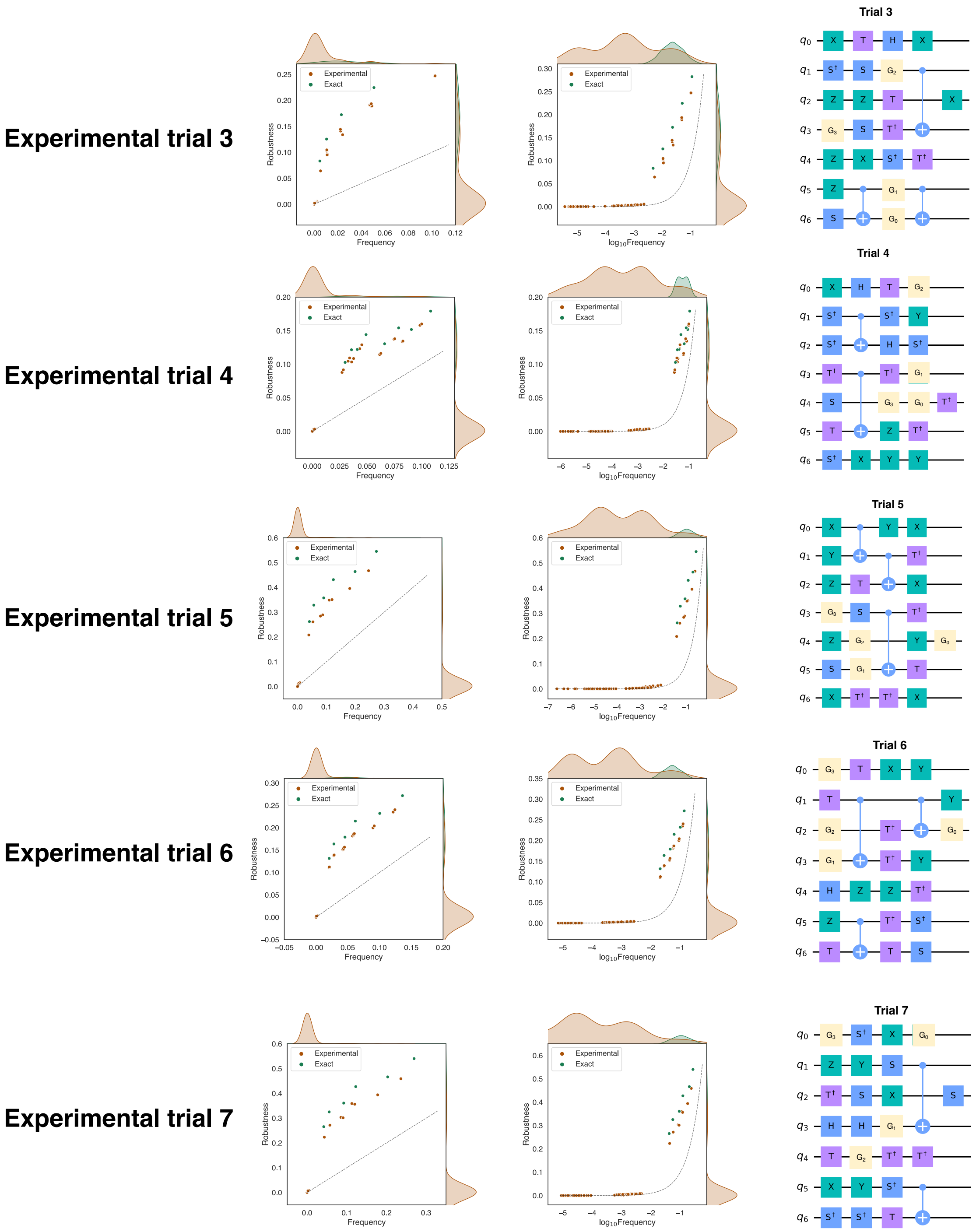

FIG. S18. Plots of (left) robustness versus frequency and (middle) robustness versus $\log_{10}$(frequency) for quantum circuit trials 3-7, as well as (right) corresponding random quantum circuits for Trials 3-7 (validation trials). The genotype is the set of variable gates $g = (G_0, G_1, G_2, G_3)$, so the length of the input sequence is $\ell = 4$ drawn from an alphabet of $k = 8$ single qubit gates: $\{Z, X, Y, H, S, S^{\dagger}, T, T^{\dagger}\}$. The dashed line is the random null expectation for both PrGP and DGP maps given by $\phi_{mn} = f_m$ for all m and n.

IX. 11 QUBIT VALIDATION TRIAL FOR QUANTUM CIRCUIT PrGP MAP

To further validate the quantum circuit PrGP map results presented in the main text and Supplemental Material, an additional validation trial with a larger circuit was conducted. In this trial, a noisy simulated quantum circuit was run with 11-qubits and 5 variable gates. Note that unlike the 7-qubit trials (which were run on the *ibm_lagos* machine), this simulation was conducted using the Qiskit Aer backend simulator with noise profile from *ibm_brisbane*.

A schematic of the random quantum circuit generated for this trial is shown in Figure S19. Figure S20 presents robustness versus frequency, robustness versus $\log_{10}$(frequency), and $\log_{10}$(robustness) versus $\log_{10}$(frequency) for this quantum circuit PrGP map validation trial. As with the 7-qubit quantum circuit PrGP map trials, these data strongly support the enhanced $\rho_n \propto \log f_n$ scaling. Again, we see the spread of phenotypes observed in the frequency domain due to superposition and/or entanglement and that many of the phentoypes are degenerate with identical frequency and robustness. This degeneracy is broken in our experimental measurements, which exhibit measurement noise. Once again, the frequency and robustness of these logarithmically scaling phenotypes is suppressed relative to the exact case as probability mass is drawn towards additional phenotypes which are observed experimentally which were not observed in the exact case. These results illustrate our suggested biphasic robustness scaling in which the low frequency phenotypes, which are introduced due to measurement noise in the experimental trials, lie much closer to the random null expectation than the higher frequency phenotypes observed in the exact calculations, which rather scale with enhanced robustness similar to what is seen in standard DGP maps.

An interesting feature of the robustness versus log frequency plots for the 11 qubit simulations is that the two phases of the robustness curve overlap, with some range of frequencies having phenotypes with both elevated log-scaling robustness, while other phenotypes have low, linear-scaling robustness. As we showed in previous sections, knowledge of ξ_n allows one to obtain even this behavior from theory.

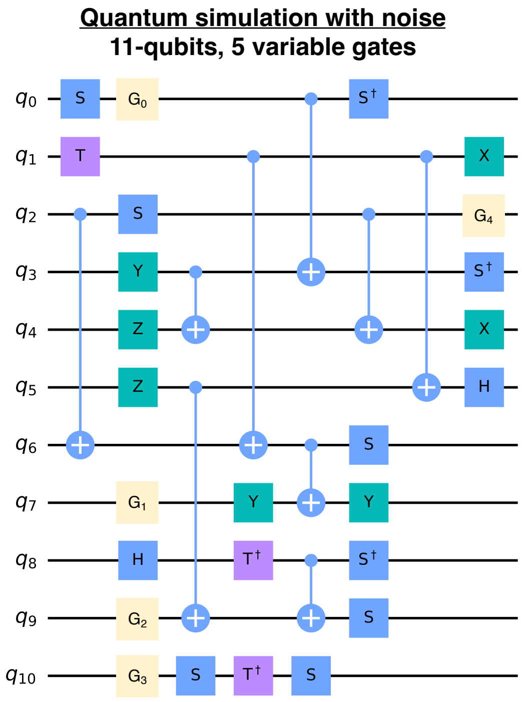

FIG. S19. Random circuit generated for 11-qubit quantum circuit trial, whose robustness data is plotted in below as a validation trial. The genotype is the set of variable gates $g = (G_0, G_1, G_2, G_3, G_4)$, so the length of the input sequence is $\ell = 5$ drawn from an alphabet of $k = 8$ single qubit gates: $\{Z, X, Y, H, S, S^\dagger, T, T^\dagger\}$.

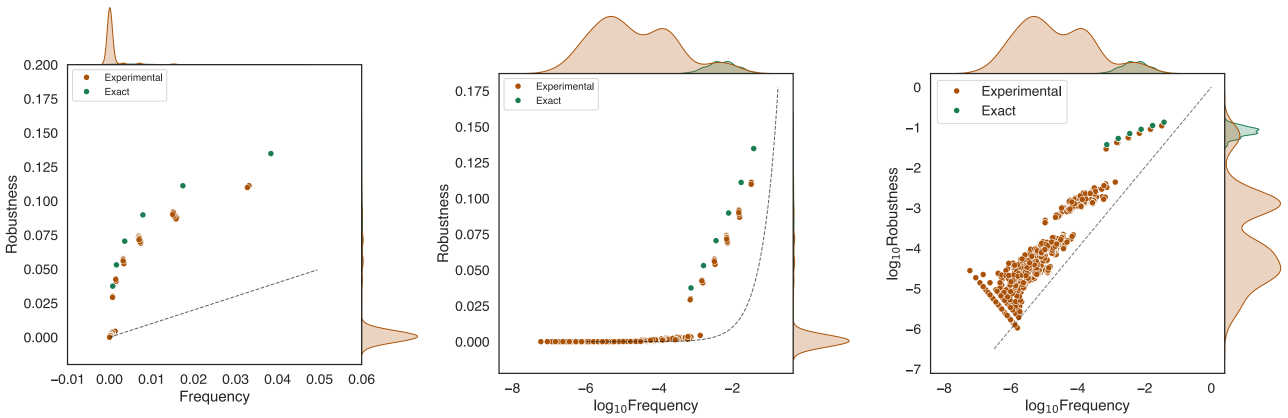

FIG. S20. Plot of (left) robustness versus frequency, (middle) robustness versus $\log_{10}$(frequency), and (right) $\log_{10}$(robustness) versus $\log_{10}$(frequency) for quantum circuit 11-qubit simulated validation trial for experimental and exact data. The dashed line is the random null expectation for both PrGP and DGP maps given by $\phi_{mn} = f_m$ for all m and n.

[1] T. Jörg, O. C. Martin, and A. Wagner, Neutral network sizes of biological RNA molecules can be computed and are not atypically small, BMC Bioinformatics **9**, 464 (2008).
[2] S. E. Ahnert, Structural properties of genotype–phenotype maps, Journal of The Royal Society Interface **14**, 20170275 (2017).
[3] S. Manrubia, J. A. Cuesta, J. Aguirre, S. E. Ahnert, L. Altenberg, A. V. Cano, P. Catalán, R. Diaz-Uriarte, S. F. Elena, J. A. García-Martín, P. Hogeweg, B. S. Khatri, J. Krug, A. A. Louis, N. S. Martin, J. L. Payne, M. J. Tarnowski, and M. Weiß, From genotypes to organisms: State-of-the-art and perspectives of a cornerstone in evolutionary dynamics, Physics of Life Reviews **38**, 55 (2021).
[4] S. F. Greenbury, S. Schaper, S. E. Ahnert, and A. A. Louis, Genetic Correlations Greatly Increase Mutational Robustness and Can Both Reduce and Enhance Evolvability, PLOS Computational Biology **12**, e1004773 (2016).
[5] J. Aguirre, J. M. Buldú, M. Stich, and S. C. Manrubia, Topological Structure of the Space of Phenotypes: The Case of RNA Neutral Networks, PLoS ONE **6**, e26324 (2011).
[6] V. Mohanty and A. A. Louis, Robustness and stability of spin-glass ground states to perturbed interactions, Physical Review E **107**, 014126 (2023), publisher: American Physical Society.
