# Supplementary material for: Probabilistic Genotype-Phenotype Maps Reveal Mutational Robustness of RNA Folding, Spin Glasses, and Quantum Circuits
Source: ArXiv. 2024 Aug 22:arXiv:2301.01847v2. Originally published 2023 Jan 4. Preprint. [Version 2] (PMC9882568)
Supplement: Supplementary file 1 [file NIHPP2301.01847v2-supplement-1.pdf]

Supplemental Materials for "Probabilistic Genotype-Phenotype Maps Reveal Mutational Robustness of RNA Folding, Spin Glasses, and Quantum Circuits"

Anna Sappington[1,2,*] and Vaibhav Mohanty[3,2,*]

[1]Department of Electrical Engineering and Computer Science, Massachusetts Institute of Technology, Cambridge, MA 02139

[2]Harvard-MIT Health Sciences and Technology, Harvard Medical School, Boston, MA 02115 and Massachusetts Institute of Technology, Cambridge, MA 02139

[3]Department of Chemistry and Chemical Biology, Harvard University, Cambridge, MA 02138

CONTENTS

I. Overview of PrGP Systems 2

II. Definitions of DGP and PrGP Map Transition Probability and Robustness 3

III. Analytical Derivation of Biphasic Robustness Curve for PrGPs 5
A. Review of Recent Analytical Results for DGP Robustness 5
B. Derivation of the Biphasic PrGP Robustness and Upper Bound 5
1. General PrGP Robustness Approximation 6
2. Upper Bound on PrGP Robustness for Small Frequencies (PrGP Robustness Tail) 7

IV. Validation of the PrGP Robustness Theory 12
A. Spin Glass PrGPs ξ_n versus f_n and Theoretical versus Empircal Robustness 12
B. RNA PrGPs ξ_n versus f_n and Theoretical versus Empircal Robustness 14
C. Quantum Circuit PrGPs ξ_n versus f_n and Theoretical versus Empircal Robustness 16

V. Phenotype Entropy Distributions for Main Text Systems 18

VI. Genotype Entropy Distributions for Various Systems 19
A. DGP Genotype Entropy 19
B. PrGP Genotype Entropy within Approximation Scheme 19
C. Maximum PrGP Genotype Entropy 19
D. Empirical Results for Genotype Entropy 20

VII. Extended Data for Main Text RNA Folding PrGP Map, GC Alphabet, $\ell = 20, k = 2$ 25

VIII. Validation Trial for RNA Folding PrGP Map, Full Alphabet, $\ell = 12, k = 4$ 28

IX. Extended Data for Main Text Spin Glass PrGP Map 31

X. Validation Trial for Spin Glass PrGP Map 35

XI. Quantum Circuit Generation Algorithm 37

XII. Extended Data for Main Text Quantum Circuit PrGP Map 38

XIII. 7 Qubit Validation Trials for Quantum Circuit PrGP Map 41

XIV. 11 Qubit Validation Trial for Quantum Circuit PrGP Map 44

References 46

* The authors contributed equally to this work. Correspondence: asappington@hms.harvard.edu and mohanty@hms.harvard.edu.

I. OVERVIEW OF PRGP SYSTEMS

Here, we provide a visualization of each of the three systems studied in this work. In the RNA folding PrGP map, each genotype maps to a Boltzmann distributed set of secondary structures which correspond to low-lying local free energy minima. We take the local free energy minima within 6 kcal of the global minima when computing each genotype's probability vector. In the spin glass PrGP map, a bond configuration for a $\pm J$ spin glass with a disordered external field on a random graph maps to a distribution of ground state spin configurations where the probability is determined by calculating the fraction of replicas in which each ground state appears. In the quantum circuit PrGP map, a random circuit is generated in which a subset of gates specify the genotype. The circuit's input state is always $|00\ldots0\rangle$, and the exact phenotype probability vector is the probability of classically measuring each basis state, $p_n(g) = |\langle n|\, U(g)\, |00\ldots0\rangle|^2$, where $U(g)$ is the circuit operation as a function of the genotype g. Experimentally, the phenotype probability vector is built from tallying classically measured states from 1000 experimental shots on the 7-qubit IBM Lagos quantum computer.

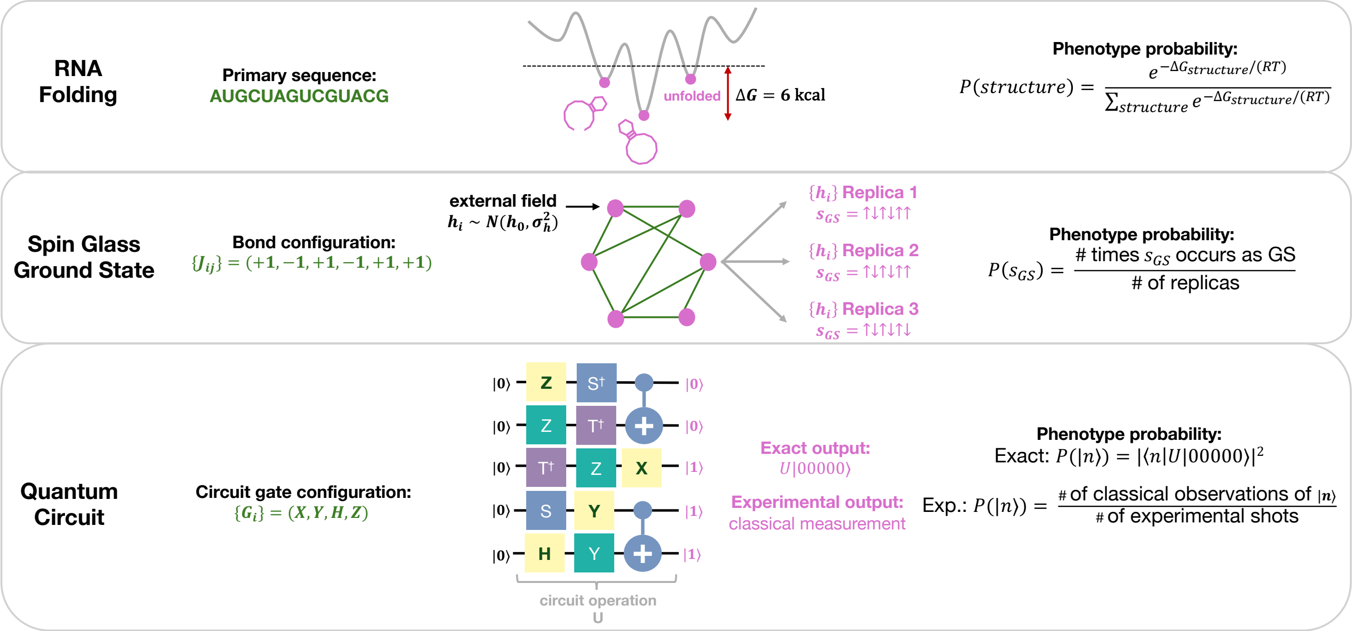

FIG. S1. Schematic representations of the PrGP model systems studied in this work. For each system, its respective genotype (green), a visualization of the system, its phenotypes (pink), and its method for calculating the phenotype probability vector are shown. For RNA folding, the genotype is a primary sequence of nucleotides and the phenotype is the folded dot-bracket structure. For spin glass ground states, the genotype is a bond configuration. Each spin s_i (pink dots) are connected via this bond configuration J_{ij} (green lines) and a disordered external field h_i is applied. The phenotype is the fraction of replicas in which each ground state appears. For quantum circuits, the genotype consists of a subset of gates from a random circuit. The circuit is given a set input state, $|00\ldots0\rangle$, and the exact phenotype probability vector is the probability of classically measuring each basis state, $p_n(g) = |\langle n|\, U(g)\, |00\ldots0\rangle|^2$, where $U(g)$ is the circuit operation as a function of the genotype g. The experimental phenotype probability vector is computed from tallying classically measured states from 1000 experimental shots on a quantum computer.

II. DEFINITIONS OF DGP AND PRGP MAP TRANSITION PROBABILITY AND ROBUSTNESS

Let a genotype-phenotype map Ω map a genotype g to phenotype n be denoted by $\Omega(g) = n$. The set of all genotypes g which map to phenotype n is called a *netural set* and is given by $\{g|\Omega(g) = n\}$. As stated in the main text, the transition probability ϕ_{mn} is the average probability that a single character mutation of a genotype mapping to phenotype n will change the phenotype to m, with the average taken over all genotypes mapping to n. This is exactly

$$\phi_{mn} = \frac{\sum_{\{g|\Omega(g)=n\}} |\{h \in \mathrm{nn}(g)|\Omega(h) = m\}|}{\ell(k-1)|\{g|\Omega(g) = n\}|}. \tag{S1}$$

The numerator is the number of neighbors of some genotype g which map to a phenotype m, averaged over all genotypes g which map to a phenotype n. The *robustness* of a phenotype n is the probability that a neighboring genotype maps back onto the same phenotype n:

$$\rho_n = \phi_{nn}. \tag{S2}$$

We note that we can write

$$|\{h \in \mathrm{nn}(g)|\Omega(h) = m\}| = \sum_{h \in \mathrm{nn}(g)} \mathbb{I}[\Omega(h) = m], \tag{S3}$$

where $\mathbb{I}[\cdot]$ is an indicator function, and similarly

$$|\{g|\Omega(g) = n\}| = \sum_{g \in S_{\ell,k}} \mathbb{I}[\Omega(g) = n], \tag{S4}$$

where $S_{\ell,k}$ is the set of all sequences of length ℓ drawn from an alphabet of k letters. We can also write the summation

$$\sum_{\{g|\Omega(g)=n\}} y(g) = \sum_{g \in S_{\ell,k}} \mathbb{I}[\Omega(g) = n] y(g). \tag{S5}$$

Putting together all three substitutions, we have

$$\phi_{mn} = \frac{\sum_{g \in S_{\ell,k}} \mathbb{I}[\Omega(g) = n] \sum_{h \in \mathrm{nn}(g)} \mathbb{I}[\Omega(h) = m]}{\ell(k-1) \sum_{g \in S_{\ell,k}} \mathbb{I}[\Omega(g) = n]}. \tag{S6}$$

Now, we relax the indicator functions to probabilites $\mathbb{I}[\Omega(g) = n] \mapsto \mathbb{P}[\Omega(g) = n]$. Note that this preserves the meaning of the transition probability—namely, that of an average probability of a change from phenotype m to n—as we now are weighting the contribution of every genotype by the probability that that genotype maps to phenotype m or n. So, we have for PrGPs,

$$\phi_{mn} = \frac{\sum_{g \in S_{\ell,k}} \mathbb{P}[\Omega(g) = n] \sum_{h \in \mathrm{nn}(g)} \mathbb{P}[\Omega(h) = m]}{\ell(k-1) \sum_{g \in S_{\ell,k}} \mathbb{P}[\Omega(g) = n]}. \tag{S7}$$

Now, writing $p_n(g) = \mathbb{P}[\Omega(g) = n]$, and rearranging the numerator, we have

$$\phi_{mn} = \frac{\sum_{g \in S_{\ell,k}} \sum_{h \in \mathrm{nn}(g)} p_n(g) p_m(h)}{\ell(k-1) \sum_{g \in S_{\ell,k}} p_n(g)}. \tag{S8}$$

We recognize that

$$\sum_{g \in S_{\ell,k}} p_n(g) = f_n k^{\ell} \tag{S9}$$

because the total probability of encountering phenotype n is still f_n. We also recognize that the sums

$$\sum_{g\in S_{\ell,k}} \sum_{h\in \mathrm{nn}(g)} y(g,h) = \sum_{\{g,h\}\in \Delta_{\ell,k}} [y(g,h) + y(h,g)], \tag{S10}$$

where the left hand summations represent a sum over all genotypes and each genotype's neighbors, whereas the right hand summations are a sum over all pairs of neighboring genotypes. We now have

$$\phi_{mn} = \frac{\sum_{\{g,h\}\in \Delta_{\ell,k}} [\mathbf{p}(g) \otimes \mathbf{p}(h) + (\mathbf{p}(g) \otimes \mathbf{p}(h))^T]_{mn}}{\ell(k-1)k^\ell f_n}, \tag{S11}$$

where we use the vector of phenotype probabilities $\mathbf{p}(g) = (p_0(g), p_1(g), \ldots)$ at a particular genotype for convenience.

III. ANALYTICAL DERIVATION OF BIPHASIC ROBUSTNESS CURVE FOR PRGPS

Here we show an analytical derivation of the biphasic robustness curve which supports the empirical observations that we report in this paper that for larger frequencies $\rho_n \propto \log f_n$ as in the DGP case and $\rho_n \propto f_n$.

A. Review of Recent Analytical Results for DGP Robustness

We first review recent exact analytical results on the upper bound of robustness in DGPs, shown recently by one of the authors [1]. In DGPs, the maximum mutational robustness for a phenotype n which has frequency $f_n = m_n k^\ell$ is given by

$$\rho_n^{\mathrm{DGP\ max}}(m_n) = \frac{2S_k(m_n)}{m_n \ell (k-1)} = \frac{\log_k m_n}{\ell} - \frac{g_k(k^{\{log_k m_n\}})}{\ell(k-1)}, \tag{S12}$$

where $\{x\}$ indicates the fractional part of x, $S_k(m) = \sum_{i=0}^{m-1} s_k(m)$, with $s_k(m)$ being the sum of all the digits in the base-k representation of integer $m = fk^\ell$, where f is the frequency of the phenotype, and

$$g_k(x) = (k-1)\log_k x + \frac{D_k(x)}{x}. \tag{S13}$$

Here, $D_k(x)$ is the modified Delange function defined by

$$D_k(x) = \sum_{n=0}^{\infty} \frac{D_{k,0}(k^x)}{k^n}, \quad D_{k,0}(x) = \int_0^x \mathrm{d}t\,(2k[t] - 2[kt] + k - 1), \tag{S14}$$

where $[x]$ is the integer part of x. The function above is a rescaling and shifting of the self-similar, continuous-everywhere, but differentiable-nowhere Takagi function or blancmange curve. The maximum mutational robustness curve is tightly bounded [1]

$$\frac{\log_k m_n}{\ell} + \frac{2A_k}{(k-1)\ell} \leq \rho_n^{\mathrm{DGP\ max}} \leq \frac{\log_k m_n}{\ell}, \tag{S15}$$

where [2] has defined

$$A_k = \frac{k}{2}\left[1 - \frac{\log\log k}{\log k} + \mathcal{O}\left(\frac{1}{\log k}\right)\right]. \tag{S16}$$

A_k is typically very small relative to the value of the robustness, so the maximum robustness is well-approximated by

$$\rho_n^{\mathrm{DGP\ max}} \approx \frac{\log_k m_n}{\ell} = 1 + \frac{\log f_n}{\ell \log k}, \quad \frac{1}{k^\ell} \leq f_n \leq 1 \tag{S17}$$

We find from empirical studies [3, 4] that empirical robustness in many DGP systems is, **due to noise in the** GP map, a suppression of the maximum robustness curve, *i.e.*

$$\rho_n^{\mathrm{DGP\ emp}} \approx a + b \log f_n. \tag{S18}$$

generally with $a < 1$ and for various b. This motivates our search for an equivalent robustness bound which, when suppressed, matches the empirical observations we have shown in RNA, spin glasses, and quantum circuits.

B. Derivation of the Biphasic PrGP Robustness and Upper Bound

To justify why biphasic robustness is necessary/expected, we first point out a critical difference between PrGPs and DGPs. DGPs have a fundamental limit on the lower bound of the frequency of a phenotype—namely, in DGPs, since a single genotype maps to a single phenotype, a phenotype with nonzero frequency must be mapped to by at least one genotype. Therefore, the lower bound on the phenotype frequency for DGPs is $f_n = 1/k^\ell$, and for this frequency the DGP robustness is always 0 since $S_k(0) = 0$; intuitively, a single genotype (node) will have zero neighbors which map

to the same phenotype, so this phenotype must necessarily have zero robustness. But, PrGPs have no such restriction on the lower bound of the frequency of a phenotype. In fact, we find many phenotypes which occur at frequencies $f_n < 1/k^\ell$. Therefore, we must expect a different scaling below $f_n < 1/k^\ell$, and perhaps even for some frequencies $f_n \geq 1/k^\ell$. We will now show this.

For sufficiently large frequencies f_n, we expect the PrGP robustness to obey at least

$$\rho_n^{\text{PrGP}} \lesssim \rho_n^{\text{DGP max}} \approx 1 + \frac{\log f_n}{\ell \log k} \tag{S19}$$

We will later be able to derive a condition for "sufficiently large." Now, consider a phenotype with small frequency f_n; for our current purposes, "small" means $f_n < 1/k^\ell$, but we will actually later find that this holds for some frequencies $f_n \geq 1/k^\ell$. Placing all of the probability mass f_n of this phenotype onto a single genotype ensures that the robustness is zero. If we were to instead map one genotype to phenotype n with probability $f_n/2$ and a neighboring genotype to the same phenotype n also with probability $f_n/2$, we'd find that the robustness is larger than zero. So, the question is, over how many nodes $\xi_n(f_n) \times k^\ell \geq 1$ should the probability mass f_n be spread out so that the robustness is maximized? Now, $\xi_n(f_n)$ is normalized to be between $1/k^\ell$ and 1.

1. *General PrGP Robustness Approximation*

We now take a graph theoretic approach. We consider the space of all genotypes to be the Hamming graph $H_{\ell,k}$, a graph in which each of the k^ℓ vertices represents a genotype, and an edge exists between two vertices if and only if those two corresponding genotype sequences differ by exactly one character. Let $G(\xi_n)$ be the subgraph containing $|V(G(\xi_n))| = \xi_n k^\ell$ number of vertices over which the n-th phenotype's probability mass is spread. It can readily be shown from the definition of robustness given in the main text (or, for example, in [1, 5]) that DGP robustness in graph theoretic terms is

$$\rho(\xi_n) = \frac{2}{\ell(k-1)} \frac{|E(G(\xi_n))|}{|V(G(\xi_n))|}, \tag{S20}$$

where $E(G(\xi_n))$ is the set of edges of $G(\xi_n)$, and $V(G(\xi_n))$ is the set of vertices of G. We now readily substitute $|V(G(\xi_n))| = \xi_n k^\ell$ to find that

$$|E(G(\xi_n))| = \frac{k^\ell \ell(k-1)}{2} \xi_n(f_n) \rho(\xi_n) \tag{S21}$$

In our approximation scheme, we assume that G is arranged to be maximally robust (if it were a DGP). Since $\xi_n(f_n) k^\ell \geq 1$, we can approximate its robustness as eq. (S70)

$$\rho_n(\xi_n) \approx 1 + \frac{\log \xi_n(f_n)}{\ell \log k}. \tag{S22}$$

Strictly, the equation above should be an inequality, but assuming that G is maximally robust means that eq. (S70) applies (see previous section). t now follows that

$$|E(G(\xi_n))| \approx \frac{k^\ell \ell(k-1)}{2} \xi_n(f_n) \left[1 + \frac{\log \xi_n(f_n)}{\ell \log k}\right]. \tag{S23}$$

Now, we can use the main text definition of transition probability to compute the PrGP robustness for a phenotype with frequency f_n whose probability mass is spread optimally (in the sense of robustness-maximizing) over $\xi_n(f_n) k^\ell$ nodes. From the main text, we have the PrGP transition probability

$$\phi_{mn} = \frac{\sum_{\{g,h\} \in \Delta_{\ell,k}} [\mathbf{p}(g) \otimes \mathbf{p}(h) + (\mathbf{p}(g) \otimes \mathbf{p}(h))^T]_{mn}}{\ell(k-1) k^\ell f_n}, \tag{S24}$$

where we can identify $\Delta_{\ell,k} \equiv E(H_{\ell,k})$ as the edge set of the Hamming graph $H_{\ell,k}$ (genotype space). For small f_n,

the maximum PrGP robustness is thus

$$\begin{aligned}
\rho_n(f_n) = \phi_{nn} &= \frac{\sum_{\{g,h\}\in\Delta_{\ell,k}}[\mathbf{p}(g)\otimes\mathbf{p}(h) + (\mathbf{p}(g)\otimes\mathbf{p}(h))^T]_{nn}}{\ell(k-1)k^\ell f_n} \\
&= \frac{\sum_{\{g,h\}\in\Delta_{\ell,k}} p_n(g)p_n(h) + p_n(h)p_n(g)}{\ell(k-1)k^\ell f_n} \\
&= \frac{2\sum_{\{g,h\}\in\Delta_{\ell,k}} p_n(g)p_n(h)}{\ell(k-1)k^\ell f_n}.
\end{aligned} \tag{S25}$$

We now note that, within our approximation scheme, for $g \in G(\xi_n)$, $p_n(g) = f_n/\xi_n(f_n)$, and for $g \notin G(\xi_n)$, $p_n(g) = 0$. Therefore,

$$\begin{aligned}
\sum_{\{g,h\}\in\Delta_{\ell,k}} p_n(g)p_n(h) &\approx \left(\frac{f_n}{\xi_n(f_n)}\right)^2 \sum_{\{g,h\}\in E(G(\xi_n))} 1 \\
&= \left(\frac{f_n}{\xi_n(f_n)}\right)^2 |E(G(\xi_n))| \\
&\approx \frac{k^\ell \ell(k-1)}{2}\frac{f_n^2}{\xi_n(f_n)}\left[1 + \frac{\log\xi_n(f_n)}{\ell\log k}\right].
\end{aligned} \tag{S26}$$

Finally, we can write that for small f_n, the maximum PrGP robustness is

$$\rho_n(f_n) \approx \frac{f_n}{\xi_n(f_n)}\left[1 + \frac{\log\xi_n(f_n)}{\ell\log k}\right], \tag{S27}$$

This an approximate upper bound due to the assumption that the probability mass is equally spread over the $\xi_n k^\ell$ nodes, though we expect it to be greater than most, if not all, real PrGP robustness values because the network of $\xi_n k^\ell$ nodes is optimally robust.

For the purposes of our paper, we use this eq. (S27) as an upper bound and suggest that some rescaling of eq. (S27), such as

$$\rho_n(f_n) \approx c + \frac{f_n}{\xi_n(f_n)}\left[a + b\frac{\log\xi_n(f_n)}{\ell\log k}\right], \tag{S28}$$

is what we expect to empirically observe in real PrGP systems, in analogy with how empirical DGP robustness $a + b\log f_n$ is a suppression of the DGP maximum in eq. (S70). Since our upper bound is approximate, and also the empirical PrGP does not strictly to be a "suppression."

Since eq. (S28) contains additional fitting parameters, we directly use eq. (S27) in most of this paper to test the theory. This is a central result of the paper.

2. *Upper Bound on PrGP Robustness for Small Frequencies (PrGP Robustness Tail)*

The first approach we take to derive the PrGP robustness tail (for small frequencies) is direct optimization of eq. (S27). We will show that this result has some special properties, but is not the tightest bound.

a. Direct Optimization of eq. (S27). We proceed by determining function $\xi_n(f_n)$ will maximize the robustness for small frequencies. By taking the functional derivative of $\rho_n(f_n)$ with respect to $\xi_n(f_n)$ and setting it to zero

$$\frac{\delta\rho_n(f_n)}{\delta\xi_n(f_n)} = -\frac{f_n}{[\xi_n(f_n)]^2}\left[1 + \frac{\log\xi_n(f_n)}{\ell\log k}\right] + \frac{f_n}{\xi_n(f_n)}\left[\frac{1}{\xi_n(f_n)\ell\log k}\right] = 0, \tag{S29}$$

we have that

$$-1 - \frac{\log\xi_n(f_n)}{\ell\log k} + \frac{1}{\ell\log k} = 0, \tag{S30}$$

from which it follows that

$$\log \xi_n(f_n) = 1 - \ell \log k, \tag{S31}$$

so

$$\xi_n(f_n) = \frac{e}{k^\ell}. \tag{S32}$$

We find that $\xi_n(f_n)$ is independent of f_n. Plugging eq. (S32) back into eq. (S27), we find that the maximum robustness (for small f_n) is

$$\rho_n^{\text{PrGP max, ``tail''}} = \frac{f_n k^\ell}{e\ell \log k}, \quad f_n < \frac{1}{k^\ell}. \tag{S33}$$

We now need to establish exactly what is the condition for "small f_n." As we mentioned previously, it is indeed possible that eq. (S33) holds as the maximum PrGP robustness for some $f_n \geq 1/k^\ell$. To check this, we compute the intersection between eq. (S33) and eq. (S70):

$$\begin{aligned} \rho_n^{\text{PrGP max, ``tail''}} &= \rho_n^{\text{DGP max}} \\ \Rightarrow \quad \frac{f_n k^\ell}{e\ell \log k} &= 1 + \frac{\log f_n}{\ell \log k} \\ \Rightarrow \quad \frac{1}{e} &= \frac{\log(f_n k^\ell)}{f_n k^\ell}. \end{aligned} \tag{S34}$$

Letting $x = -\log(f_n k^\ell)$, we can rewrite the above equation as

$$x e^x = -\frac{1}{e}, \tag{S35}$$

whose solution is

$$x = W_0\left(-\frac{1}{e}\right) = W_{-1}\left(-\frac{1}{e}\right) = -1, \tag{S36}$$

where W_0 and W_{-1} are the two branches of the Lambert W function, and $-1/e$ is the point at which the two branches meet and produce the same value. It thus follows that $x = -1 = -\log(f_n k^\ell)$, so the point of intersection of the two curves is

$$f_n^{\text{intersection}} = \frac{e}{k^\ell}. \tag{S37}$$

It is quick to verify that for $f_n > \frac{e}{k^\ell}$, the DGP maximum eq. (S70) has higher robustness than the tail function eq. (S33), and for $f_n < \frac{e}{k^\ell}$, the DGP maximum eq. (S70) has lower robustness than the tail function eq. (S33).

We can now bound the PrGP robustness from above with the following piecewise smooth function:

$$\rho_n^{\text{PrGP upper}}(f_n) = \begin{cases} \dfrac{f_n k^\ell}{e\ell \log k} & f_n \leq \dfrac{e}{k^\ell} \\ 1 + \dfrac{\log f_n}{\ell \log k} & f_n \geq \dfrac{e}{k^\ell}. \end{cases} \tag{S38}$$

Although this is not the tightest possible upper bound on the robustness tail (shown in the next section), the above has some nice properties. First, both parts of the piecewise function are closely related to or derived from eq. (S27), and eq. (S38) can be equivalently written as

$$\xi_n(f_n) = \begin{cases} \dfrac{e}{k^\ell} & f_n \leq \dfrac{e}{k^\ell} \\ f_n & f_n \geq \dfrac{e}{k^\ell}. \end{cases} \tag{S39}$$

Next, we can show that eq. (S38) is once differentiable everywhere. We only need to show that the derivatives match at $f_n = e/k^\ell$. From the left,

$$\frac{\partial \rho_n^{\text{PrGP upper}}}{\partial f_n} = \frac{k^\ell}{e\ell \log k}, \quad f_n \leq \frac{e}{k^\ell}, \tag{S40}$$

so

$$\left.\frac{\partial \rho_n^{\text{PrGP upper}}}{\partial f_n}\right|_{f_n \to \frac{e}{k^\ell}^-} = \frac{k^\ell}{e\ell \log k}. \tag{S41}$$

and from the right

$$\frac{\partial \rho_n^{\text{PrGP upper}}}{\partial f_n} = \frac{1}{f_n \ell \log k}, \quad f_n \geq \frac{e}{k^\ell}, \tag{S42}$$

so

$$\left.\frac{\partial \rho_n^{\text{PrGP upper}}}{\partial f_n}\right|_{f_n \to \frac{e}{k^\ell}^+} = \frac{k^\ell}{e\ell \log k}. \tag{S43}$$

It is clear that eq. (S38) is an upper bound and once differentiable everywhere.

To interpret the above derivation, we consider a phenotype of frequency $f_n \leq 1/k^\ell$. The derivation in this section shows that by leaving the f_n probability mass on a single genotype, the robustness is less than optimal (it is zero). It suggests that robustness could be optimized by spreading out the probability mass f_n over e genotypes. (Since the number of genotypes can be only integer valued, this means, that the phenotype would need to be spread over 2 or 3 nodes.) However, below we show that spreading the phenotype over e nodes puts the actual robustness far enough below the bound eq. (S38) such that higher-robustness configurations can be constructed which are still below eq. (S38). Therefore, eq. (S38) is not the tightest possible bound, and we derive a tighter one below.

b. Tighter Bound on PrGP Robustness Tail. Consider a phenotype with small frequency $f_n \leq k/k^\ell = k^{1-\ell}$. Now, suppose that this frequency is spread over exactly N genotypes, with $1 \leq N \leq k$. Since $N \leq k$, it is possible to form a complete graph with these N nodes, which will have exactly $N(N-1)/2$ edges. Below, we will show that the PrGP robustness will be maximized by spreading probability mass evenly over N nodes arranged in a complete graph, which can only occur if $N \leq k$.

Theorem III.1. *A phenotype with $f_n \leq k/k^\ell = k^{1-\ell}$ spread over a complete graph with N nodes, with $1 \leq N \leq k$, will have maximum PrGP robustness when the phenotype probability is equal across all nodes and $N = k$. This maximum robustness is*

$$\rho = \frac{f_n k^{\ell-1}}{\ell}, \quad 0 < f_n \leq k^{1-\ell}. \tag{S44}$$

Proof. We can write down the adjacency matrix of the complete graph as $A = \mathbb{1}\mathbb{1}^T - \mathbb{I}$, where $\mathbb{I}$ is the $N \times N$ identity matrix, and $\mathbb{1}$ is the length N vector of all ones. Letting $p_g \equiv p_n(g)$, the PrGP robustness is exactly

$$\begin{aligned} \rho(\mathbf{p}) &= \frac{2}{\ell(k-1)k^\ell f_n}\left[\frac{1}{2}\sum_{g,h} p_g A_{gh} p_h\right] \\ &= \frac{1}{\ell(k-1)k^\ell f_n}\left[\left(\sum_g p_g\right)^2 - \sum_g p_g^2\right]. \end{aligned} \tag{S45}$$

Using a Lagrange multiplier λ, we impose a normalization constraint on the probability vector with the Langrangian

$$\mathcal{L}[\mathbf{p}] = \rho(\mathbf{p}) - \lambda\left(\sum_g p_g - f_n k^\ell\right). \tag{S46}$$

We optimize the Lagrangian

$$
\begin{aligned}
0 = \frac{\delta \mathcal{L}}{\delta p_h} &= \frac{1}{\ell(k-1)k^\ell f_n}\left[2\left(\sum_g p_g\right)\sum_g \delta_{gh} - 2\sum_g p_g \delta_{gh}\right] - \lambda\left(\sum_g \delta_{gh}\right) \\
&= \frac{2}{\ell(k-1)k^\ell f_n}\left[\sum_g p_g - p_h\right] - \lambda,
\end{aligned}
\tag{S47}
$$

and

$$
\begin{aligned}
0 = \frac{\delta \mathcal{L}}{\delta \lambda} &= -\left(\sum_g p_g - f_n k^\ell\right) \\
\Rightarrow \quad & \sum_g p_g = f_n k^\ell.
\end{aligned}
\tag{S48}
$$

Summing eq. (S47) over h and plugging in the normalization constraint, we have

$$
\begin{aligned}
0 &= \frac{2}{\ell(k-1)k^\ell f_n}\left(N f_n k^\ell - \sum_h p_h\right) - N\lambda \\
&= \frac{2}{\ell(k-1)k^\ell f_n}\left(N f_n k^\ell - f_n k^\ell\right) - N\lambda,
\end{aligned}
\tag{S49}
$$

so

$$
\lambda = \frac{2(N-1)}{N\ell(k-1)}. \tag{S50}
$$

Plugging this into eq. (S47), we have

$$
0 = \frac{2}{\ell(k-1)k^\ell f_n}\left[f_n k^\ell - p_h\right] - \frac{2(N-1)}{N\ell(k-1)}, \tag{S51}
$$

from which it follows that

$$
p_h = \frac{f_n k^\ell}{N} \tag{S52}
$$

for any genotype h. This means that for a complete graph with N nodes, PrGP robustness is optimized by spreading the phenotype probability equally over all N nodes.

We can now plug in this result into eq. (S45) to find the robustness in terms of N:

$$
\begin{aligned}
\rho(N) &= \frac{1}{\ell(k-1)k^\ell f_n}\left[f_n^2 k^{2\ell} - \sum_g \frac{f_n^2 k^{2\ell}}{N^2}\right] \\
&= \frac{f_n k^\ell}{\ell(k-1)}\left[1 - \frac{1}{N}\right].
\end{aligned}
\tag{S53}
$$

Since $1 \leq N \leq k$ and N is an integer, it is clear that $\rho(N)$ is maximized when $N = k$, so the maximum robustness is

$$
\rho(N=k) = \frac{f_n k^\ell}{\ell(k-1)}\left[1 - \frac{1}{k}\right] = \frac{f_n k^{\ell-1}}{\ell}. \tag{S54}
$$

This completes the proof. □

For $f_n \leq k^{1-\ell}$, we have now provided the tightest possible upper bound on robustness, and we point out the critical point that the tail in Theorem III.1 still scales as $\rho_n \propto f_n$, just like in eq. (S38). It can easily be verified that the bound from Theorem III.1 is strictly lower/tighter than eq. (S38) by comparing the slopes; that is, $k^{\ell-1}\ell \geq k^\ell/(e\ell \log k)$, with equality when $k = e$. In terms of interpretation, we also now understand that spreading the phenotype over e

nodes, as suggested by eq. (S38), does not produce a robustness that is as high as spreading the phenotype over k nodes.

We now compute the intersection between the robustness from Theorem III.1 and the approximation to the DGP maximum robustness in eq. (S70):

$$\begin{aligned} \frac{f_n k^{\ell-1}}{\ell} &= 1 + \frac{\log f_n}{\ell \log k} \\ \Rightarrow \quad \frac{(f_n k^{\ell})}{k\ell} &= \frac{\log\big(f_n k^{\ell}\big)}{\ell \log k}. \end{aligned} \tag{S55}$$

By inspection we can see that $f_n k^\ell = k$ (i.e. $f_n = k^{1-\ell}$) is a solution to the above equation and provides the point of intersection between the two robustness curves. This leads to a tighter upper bound on robustness:

$$\rho_n^{\text{PrGP upper}}(f_n) = \begin{cases} \dfrac{f_n k^{\ell-1}}{\ell} & f_n \leq k^{1-\ell} \\ \\ 1 + \dfrac{\log f_n}{\ell \log k} & f_n \geq k^{1-\ell}. \end{cases} \tag{S56}$$

This form is not, in general, differentiable at $f_n = k^{1-\ell}$, unlike eq. (S38). The derivative from the left is

$$\frac{\partial \rho_n^{\text{PrGP upper}}}{\partial f_n} = \frac{k^{\ell-1}}{\ell}, \quad f_n \leq k^{1-\ell}, \tag{S57}$$

so

$$\left.\frac{\partial \rho_n^{\text{PrGP upper}}}{\partial f_n}\right|_{f_n \to k^{1-\ell-}} = \frac{k^{\ell-1}}{\ell}. \tag{S58}$$

and from the right

$$\frac{\partial \rho_n^{\text{PrGP upper}}}{\partial f_n} = \frac{1}{f_n \ell \log k}, \quad f_n \geq k^{1-\ell}, \tag{S59}$$

so

$$\left.\frac{\partial \rho_n^{\text{PrGP upper}}}{\partial f_n}\right|_{f_n \to k^{1-\ell+}} = \frac{k^{\ell-1}}{\ell \log k}. \tag{S60}$$

Equating the two derivatives, using similar analysis as eq. (S35), we find that the first derivatives only match when $k = e$. Thus, since k only takes on integer values ≥ 2, in general the robustness upper bound is not differentiable at $f_n = k^{1-\ell}$.

IV. VALIDATION OF THE PRGP ROBUSTNESS THEORY

We return to a discussion eq. (S27), which is an approximate upper bound on the PrGP, but we use its functional form as an empirical approximation for the real PrGP robustness under the approximations that (1) probability mass for a phenotype n is equally spread over $\xi_n k^\ell$ nodes and that (2) the $\xi_n k^\ell$ are maximally robust. We would expect that that empirical PrGP looks like some rescaling eq. (S27) in analogy with how empirical DGP robustness looks relative to the rigorously proven DGP maximum.

We now examine $\xi_n(f_n)$. In general, we expect ξ_n to be positively correlated with f_n. The simplest analytical form that we can assume for their relationship is $\xi_n(f_n) \approx \alpha + \beta f_n$, with the DGP maximum robustness emerging when $\alpha = 0$ and $\beta = 1$. The resulting PrGP robustness, plugging this approximation into eq. (S27), would be

$$\rho_n(f_n) \approx \frac{f_n}{\alpha + \beta f_n}\left[1 + \frac{\log(\alpha + \beta f_n)}{\ell \log k}\right] \tag{S61}$$

In the limit of small frequencies $f_n \ll |\alpha/\beta|$, the above equation reduces to

$$\rho_n(f_n) \approx \frac{f_n}{\alpha}\left[1 + \frac{\log(\alpha)}{\ell \log k}\right] \sim f_n. \tag{S62}$$

In the limit of larger frequencies $f_n \gg |\alpha/\beta|$, the equation reduces to

$$\rho_n(f_n) \approx \frac{1}{\beta}\left(1 + \frac{\log \beta}{\ell \log k}\right) + \frac{\log(f_n)}{\beta \ell \log k} \sim a + b \log f_n. \tag{S63}$$

This suggests that the functional form eq. (S27) produces the empirically observed biphasic in the limits of low and high frequencies, though there are many other functional forms which would yield the same biphasic robustness curves, or at least ones which are difficult to distinguish from the scaling laws presented here.

One commonly observed scaling between ξ_n and f_n seen for small frequencies in RNA and for all frequencies in quantum circuits is a power law: $\xi_n \approx \alpha f_n^\eta$. This formula can also be used

In particular, as we see in the subsequent sections, a sublinear power law $0 < \eta < 1$ is common. This leads to a theoretical robustness of

$$\rho_n(f_n) = \frac{f_n^{1-\eta}}{\alpha}\left(1 + \frac{\log \alpha}{\ell \log k} + \eta \frac{\log f_n}{\ell \log k}\right). \tag{S64}$$

We can see that this formula interpolates between the linear and logarithmic scaling. In the limit of $\eta = 0$, it is clear that $\rho_n \sim f_n$, while in the limit of $\eta = 1$, we have $\rho \approx a + b \log f_n$. For intermediate $0 < \eta < 1$, we have

$$\rho_n(f_n) \approx \frac{\eta f_n^{1-\eta} \log f_n}{\alpha \ell \log k} \sim f_n^{1-\eta}(a + b\eta \log f_n), \tag{S65}$$

and for high frequencies we make the substitution $x = \log f_n$ and expand for small x:

$$\rho_n(f_n) \approx \frac{1}{\alpha}\left(1 + \frac{\log \alpha}{\ell \log k} + \frac{\eta x}{\ell \log k}\right) \sim a + b\eta \log f_n. \tag{S66}$$

In the following subsections, we examine the validity of the theoretical formulas in eq. (S27) and eq. (S64) to the empirical systems studied in this paper.

A. Spin Glass PrGPs ξ_n versus f_n and Theoretical versus Empircal Robustness

The spin glass PrGPs, especially the $\sigma_h^2 = 0.001$ and $\sigma_h^2 = 0.01$ cases, show excellent agreement with the theory, including the approximations in this subsection section.

In the ξ_n versus f_n plots in Figure S2, for $\sigma_h^2 = 0.001$, we see that $\xi_n \approx f_n$ for f_n above a certain threshold, while $\xi_n \approx \alpha$ for some constant α frequencies below the threshold. Similar behavior is observed for the $\sigma_h^2 = 0.01$ case, though the flattening out of the curve for small f_n is more apparent on the log-log plot. For the $\sigma_h^2 = 0.1$ case, the curve is mostly constant $\xi_n \approx \alpha$ for nearly all frequencies, with a small f_n tail which is too diffuse to quantify with a scaling law. This leads to $\rho_n \sim f_n$ for the vast majority of frequencies, which is the same scaling law as the null

System	Details	Theory vs. Real Pearson r	Null vs. Real Pearson r	DGP Max vs. Real Pearson r
Spin Glass	$\sigma_h^2 = 0.001$	**0.979**	0.766	0.940
Spin Glass	$\sigma_h^2 = 0.01$	**0.956**	0.874	0.924
Spin Glass	$\sigma_h^2 = 0.1$	**0.976**	0.976	0.954
RNA GC20	20 °C	**0.967**	0.811	0.797
RNA GC20	37 °C	**0.978**	0.854	0.766
RNA GC20	70 °C	**0.859**	0.856	0.665
RNA12	37 °C	**0.884**	0.832	0.882
Quantum Circuit	11 qubit (exact)	**0.9996**	0.901	0.997
Quantum Circuit	11 qubit (simulation)	**0.981**	0.865	0.576
Quantum Circuit	7 qubit, trial 1 (exact)	**0.9997**	0.926	0.998
Quantum Circuit	7 qubit, trial 1 (exp.)	**0.961**	0.912	0.712

TABLE S1. Pearson correlation coefficient r between robustness predicted from eq. (S27) versus empirically measured robustness. In general, the theory outperforms the null model and the DGP maximum approximations, and overall Pearson correlations are very high, close to 1, highlighting the success of eq. (S27). Bold indicates the best performing model.

model.

In the ρ_n vs $\log f_n$ plots in Figure S3 in which we show both theoretical ρ_n (given empirical values of f_n and $\xi_n(f_n)$) and the empirical ρ_n, we find excellent agreement in the scaling trends in all three cases. This is quantified by the high Pearson correlation coefficients, as shown in Table S1. Notably, as the disorder increases, the null model gets better at predicting the robustness; this is explained above by the fact that $\xi_n = \alpha$ is essentially constant for the $\sigma_h^2 = 0.001$ case. It should be emphasized that in these plots, zero fitting parameters have been used; only the f_n and $\xi_n(f_n)$ data have been plugged into eq. (S27).

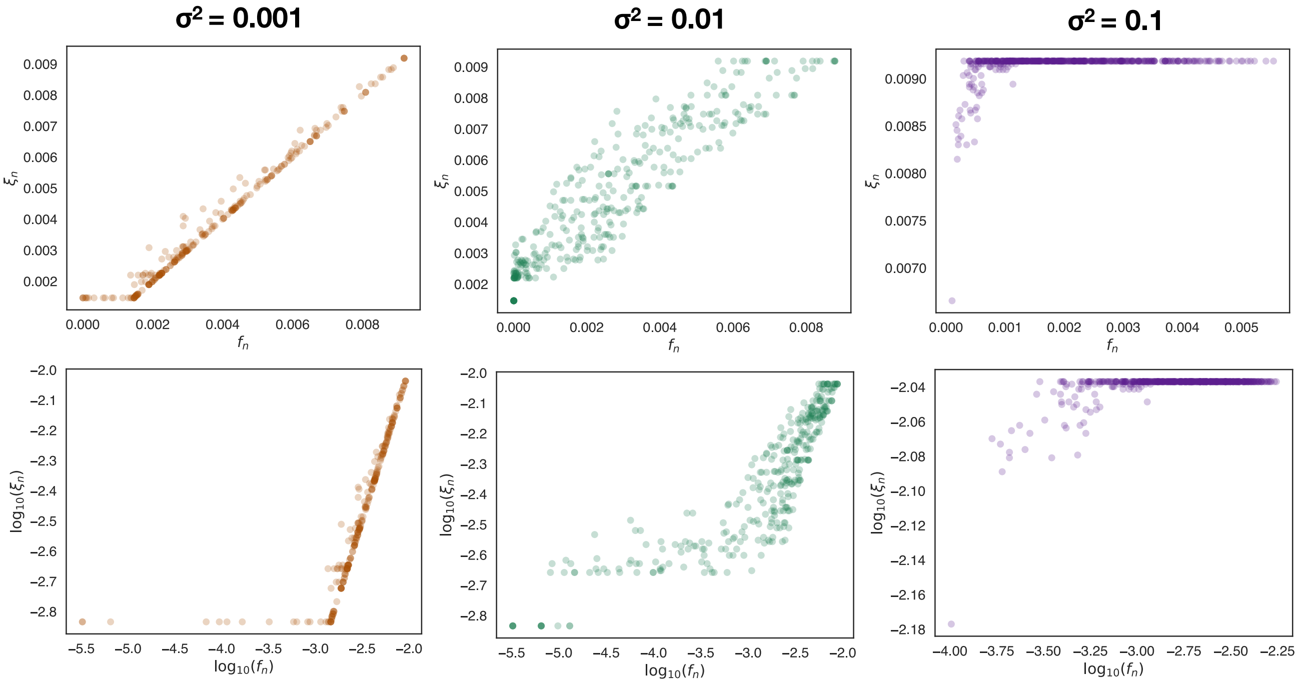

FIG. S2. Plots of (top) phenotype frequency f_n versus ξ_i and (bottom) $\log_{10}(f_n)$ versus $\log_{10}(\xi_n)$ for the spin glass ground state main text system at three levels of external field disorder ($\sigma_h^2 = 0.001, \sigma_h^2 = 0.01, \sigma_h^2 = 0.1$).

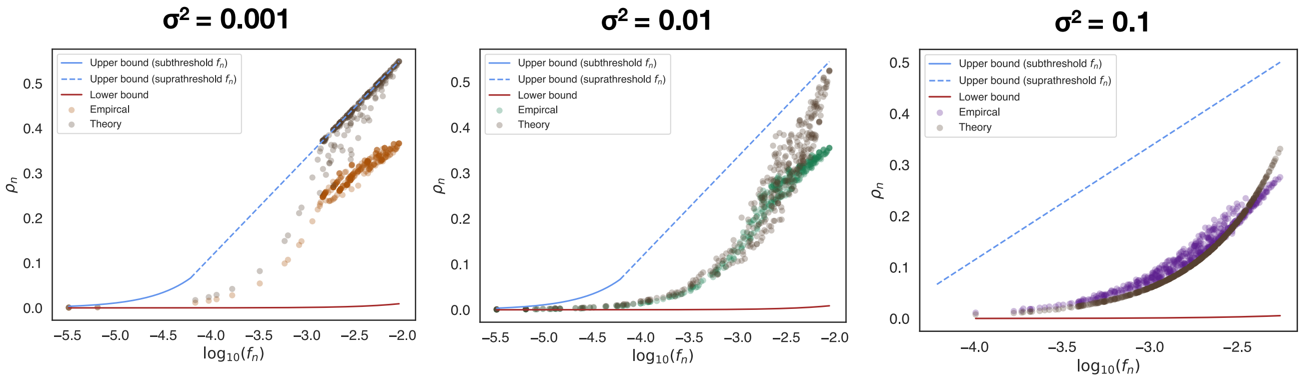

FIG. S3. Plots of $\log_{10}(f_n)$ versus ρ_n for each phenotype n for the spin glass ground state main text system at three levels of external field disorder ($\sigma_h^2 = 0.001, \sigma_h^2 = 0.01, \sigma_h^2 = 0.1$). Here, empirical points are the true $\log_{10}(f_n)$ and ρ_n values calculated from the data while theory points have been computed using eq. (S27) with zero fitting parameters. Also shown is the lower bound (null model) and upper bound for subthreshold f_n and suprathreshold f_n as derived in eq. (S56).

B. RNA PrGPs ξ_n versus f_n and Theoretical versus Empircal Robustness

In RNA PrGPs, the ξ_n versus f_n plots in fig. S4 show much less clear monotonic relationship. The log-log plots, however, suggest a power law for small frequencies, which (fits not shown here) appears to be sublinear (exponent $\eta \approx 0.68$ for GC20 20 °C, $\eta \approx 0.70$ for GC20 37 °C, $\eta \approx 0.82$ for GC20 70 °C). For GC20, the theory therefore predicts $\rho_n \sim (a + b\eta \log f_n)$ for small f_n, as derived from eq. (S64). The RNA12 model has such a small number of phenotypes that no clear scaling law is apparent.

The plots in fig. S5 show the real robustness plotted with the theoroetical robustness. The theory does an excellent job at predicting the real robustness, as evidenced by the Perason correlation coefficients in Table S1. As the temperature in the GC20 systems increases, we see that the null model and theory have essentially the same Pearson correlation coefficient. Increased disorder means that not only do phenotypes become more spread out across genotypes, but also the probability vectors at each genotype are noisier. The null model's prediction comes from evenly spreading out all phenotypes over the entire genotype space (see section below on Genotype Entropy Distributions); in the limit where disorder is high, this picture becomes closer and closer to reality.

It should be emphasized that in these plots, zero fitting parameters have been used; only the f_n and $\xi_n(f_n)$ data have been plugged into eq. (S27).

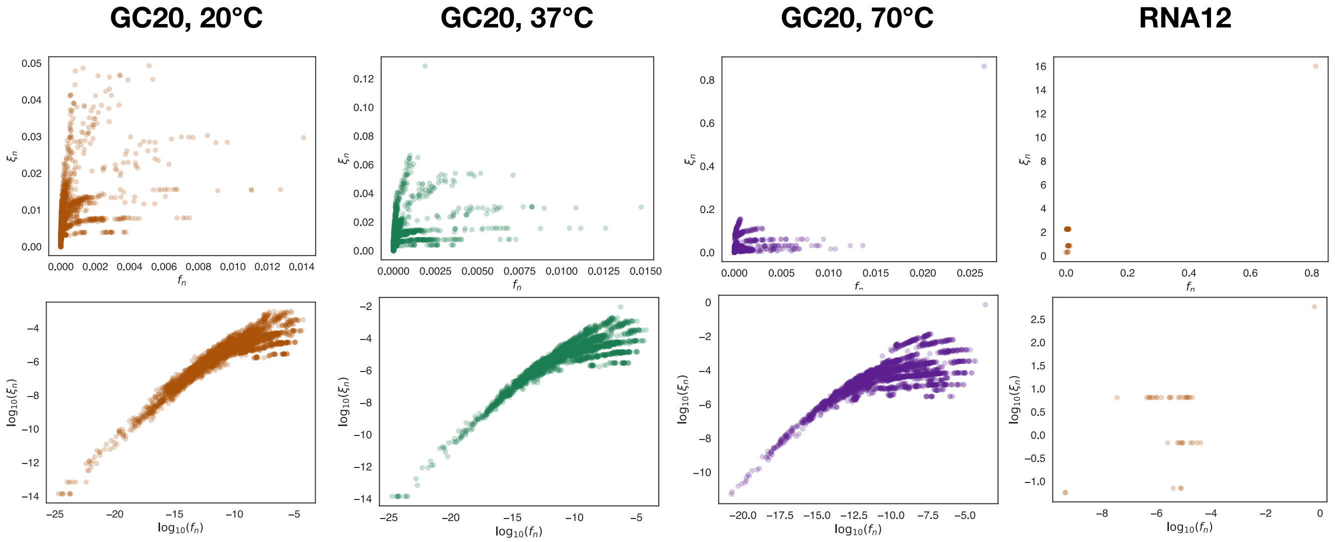

FIG. S4. Plots of (top) phenotype frequency f_n versus ξ_i and (bottom) $\log_{10}(f_n)$ versus $\log_{10}(\xi_n)$ for the RNA folding system at three temperatures $(20\,^{\circ}\mathrm{C}, 37\,^{\circ}\mathrm{C}, 70\,^{\circ}\mathrm{C})$ for $k = 2, \ell = 20$ and at $37\,^{\circ}\mathrm{C}$ for $k = 4, \ell = 12$.

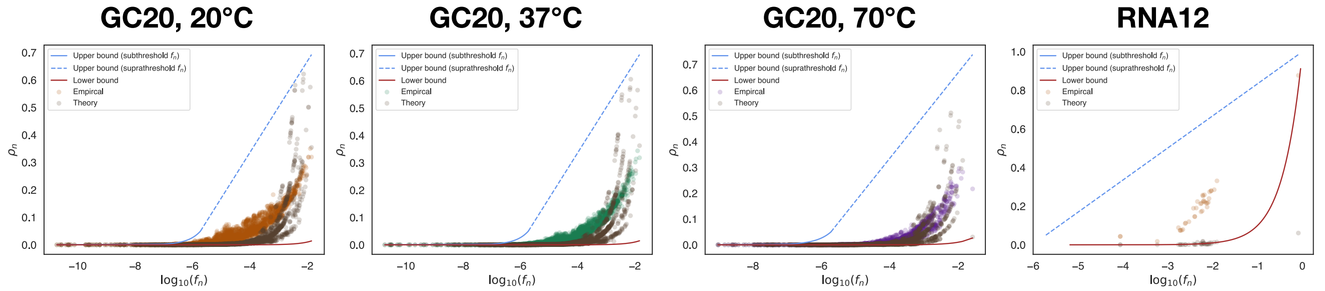

FIG. S5. Plots of $\log_{10}(f_n)$ versus ρ_n for each phenotype n for the RNA folding system at three temperatures $(20\,^{\circ}\mathrm{C}, 37\,^{\circ}\mathrm{C}, 70\,^{\circ}\mathrm{C})$ for $k = 2, \ell = 20$ and at $37\,^{\circ}\mathrm{C}$ for $k = 4, \ell = 12$. Here, empirical points are the true $\log_{10}(f_n)$ and ρ_n values calculated from the data while theory points have been computed using eq. (S27) with zero fitting parameters. Also shown is the lower bound (null model) and upper bound for subthreshold f_n and suprathreshold f_n as derived in eq. (S56).

C. Quantum Circuit PrGPs ξ_n versus f_n and Theoretical versus Empircal Robustness

In the quantum circuit PrGPs, the exact PrGPs (with no measurement noise), have a small number of points, but they seem to suggest a power law relationship $\xi_n \approx \alpha f_n^{\eta}$; for the 11 qubit example, the exponent for the power law was sublinear: $\eta \approx 0.88$. This would lead to a predicted robustness given by eq. (S64), which we report has high Pearson correlation (essentially 1.0 to several decimal places) with the exact empirical data (with no measurement noise) when compared to the measured robustness; however, of course there are only a very small number of data points.

When examining experimental (i.e. with measurement noise) curves in Figure S6 for ξ_n versus f_n, the plots suggest that the relationship between ξ_n and f_n for several groups of phenotypes behaves like a power law, but there are several groups of phenotypes with different power laws.

In the plots of theoretical versus actual robustness in Figure S7, we again find excellent agreement for both the main text 7 qubit experiment and 11 qubit simulated experiment, as greatly evidenced by high Pearson correlation coefficients between the real and theoretical robustness.

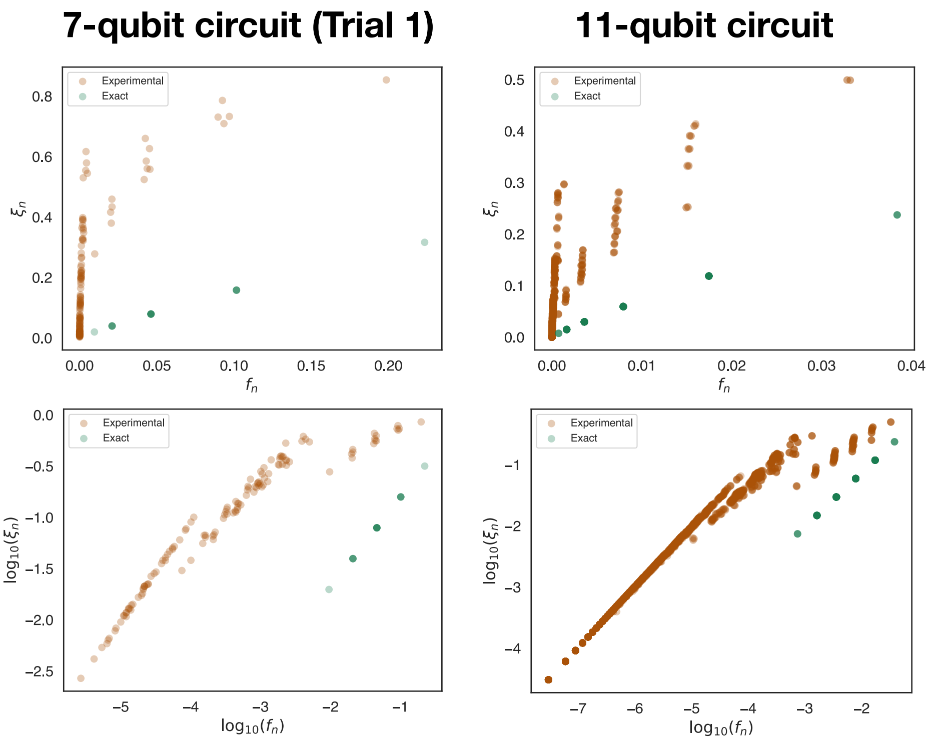

FIG. S6. Plots of (top) phenotype frequency f_n versus ξ_i and (bottom) $\log_{10}(f_n)$ versus $\log_{10}(\xi_n)$ for the quantum circuit system for the main text 7-qubit circuit trial (exact and experimental results) and the 11-qubit circuit validation trial (exact and simulated results).

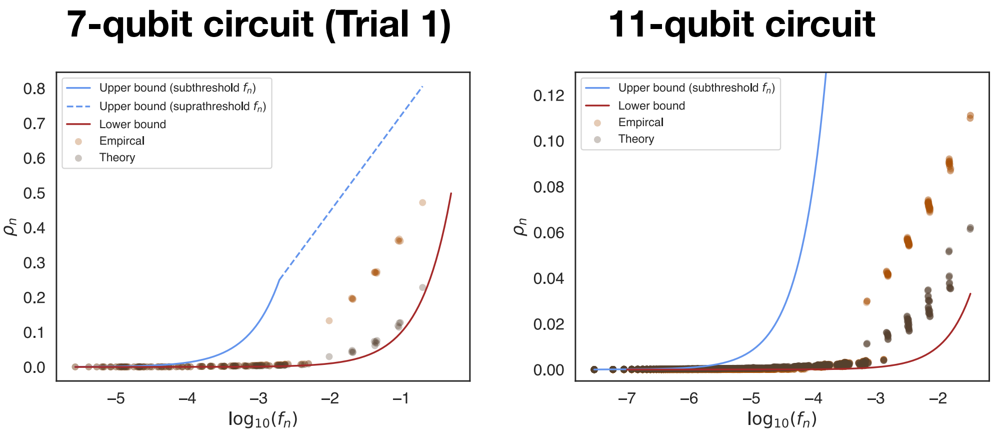

FIG. S7. Plots of $\log_{10}(f_n)$ versus ρ_n for each phenotype n for the quantum circuit system for the main text 7-qubit circuit trial (exact and experimental results) and the 11-qubit circuit validation trial (exact and simulated results). Here, empirical points are the true $\log_{10}(f_n)$ and ρ_n values calculated from the data while theory points have been computed using eq. (S27) with zero fitting parameters. Also shown is the lower bound (null model) and upper bound for subthreshold f_n and suprathreshold f_n as derived in eq. (S56).

V. PHENOTYPE ENTROPY DISTRIBUTIONS FOR MAIN TEXT SYSTEMS

In the main text, we presented robustness versus frequency plots for RNA folding, spin glass ground state, and quantum circuit PrGP maps. For the spin glass ground state and quantum circuit PrGP maps, data for a single representative realization were presented in the main text. In Figure S8, we plot the distribution of phenotype entropy $S(g)$ across all genotypes g for each of these PrGP maps. Recall that phenotype entropy is defined in the main text as

$$S(g) = -\sum_{n\in\{\text{phenotypes}\}} p_n(g)\log p_n(g). \tag{S67}$$

For RNA folding and spin glasses, we observe that the phenotype entropy distributions shift rightward as the disorder parameter increases. For RNA, this corresponds to increasing temperature and for spin glasses, this corresponds to increasing external field variance σ_h^2. For the quantum circuits, we plot both exact and experimental results (from the 7-qubit IBM quantum computer); the experimental phenotype entropy distribution is shifted rightward relative to the exact result, due to measurement noise as well as a finite number of experimental trials.

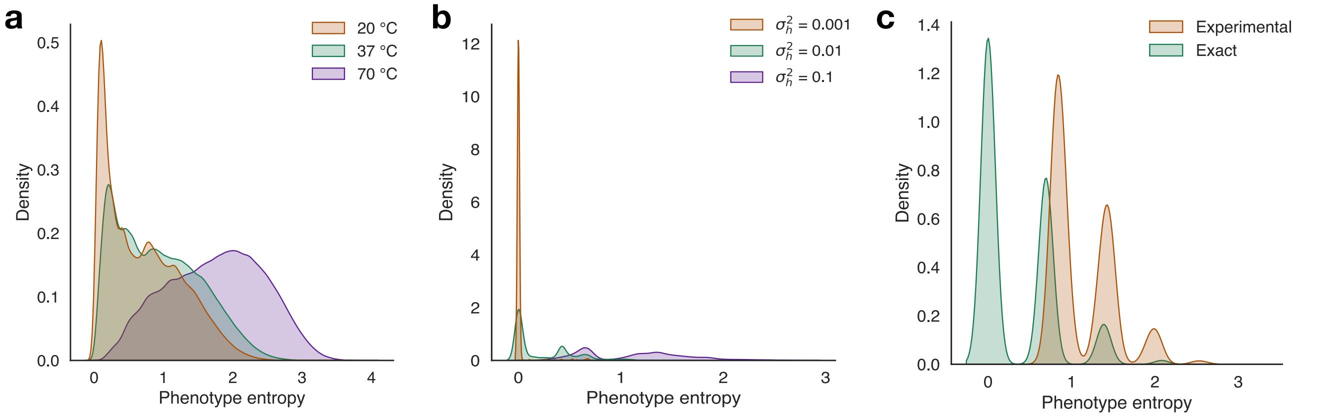

FIG. S8. Phenotype entropy distributions for the (a) RNA folding, (b) spin glass ground state, and (c) quantum circuit PrGP maps whose robustness plots are presented in the main text. As disorder parameters increase in (a) and (b) due to increased temperature and increased external field variance, respectively, the entropies shift rightward. The same occurs due to measurement noise in (c).

VI. GENOTYPE ENTROPY DISTRIBUTIONS FOR VARIOUS SYSTEMS

In the main text, we define the genotype entropy for a phenotype n as

$$S_n^\gamma = -\sum_{g\in\{\text{genotypes}\}} \frac{p_n(g)}{f_n k^\ell} \log \frac{p_n(g)}{f_n k^\ell}. \tag{S68}$$

where the factor $f_n k^\ell$ in the probabilities ensures that $\frac{p_n(g)}{f_n k^\ell}$ can be treated as a probability mass since

$$\sum_{g\in\{\text{genotypes}\}} \frac{p_n(g)}{f_n k^\ell} = 1. \tag{S69}$$

We now derive a few key analytical results.

A. DGP Genotype Entropy

First, the relationship between genotype entropy S_n^γ and frequency f_n for DGP systems can be analytically derived, since $p_n(g)$ takes on values of 0 or 1 for each genotype g. Letting $p_n(g) = 1$ means that

$$S_n^{\gamma,\text{DGP}} = -\sum_{g\in\{\text{genotypes}\}} \frac{1}{f_n k^\ell} \log \frac{1}{f_n k^\ell} = -\log \frac{1}{f_n k^\ell} = \ell \log k + \log f_n. \tag{S70}$$

Therefore, for DGPs, the scaled genotype entropy $S_n^{\gamma,\text{DGP}}/(\ell \log k)$ is equal to the asymptotic maximum robustness. All DGPs will have genotype entropy which falls along this curve.

B. PrGP Genotype Entropy within Approximation Scheme

Second, we obtain the genotype entropy within the approximation scheme used to derive the PrGP robustness in eq. (S27): namely, we assume that the phenotype has frequency f_n, is spread over $\xi_n(f_n)k^\ell$ genotypes, and has equal probability across all of these genotypes. From the normalization rule, we must have that

$$\xi_n(f_n)k^\ell p_n(g) = f_n k^\ell, \tag{S71}$$

so the probability on any node will be

$$p_n(g) = \frac{f_n}{\xi_n(f_n)}. \tag{S72}$$

It follows that

$$S_n^{\gamma,\text{PrGP approx}} = -\sum_{g\in\{\text{genotypes}\}} \frac{1}{\xi_n(f_n)k^\ell} \log \frac{1}{\xi_n(f_n)k^\ell} = \ell \log k + \log \xi_n(f_n). \tag{S73}$$

C. Maximum PrGP Genotype Entropy

Third, we can now find the maximum PrGP robustness. Since there are no constraints other than normalization, it would be straightforward to show that entropy should be maximized when probability mass is equally spread over all genotypes that map at all to phenotype n. This is the same as maximizing eq. (S73) with respect to $\xi_n(f_n)k^\ell$ over the domain of integers $1, \ldots, k^\ell$. It is clear that for $\xi_n(f_n)k^\ell = k^\ell$ (or $\xi_n(f_n) = 1$), we have that

$$S_n^{\gamma,\text{PrGP max}} = \ell \log k, \tag{S74}$$

regardless of the phenotype frequency f_n.

D. Empirical Results for Genotype Entropy

In Figure S9, we plot the genotype entropy S_n^γ for all phenoypes n for each of the three RNA GC20 PrGP map temperatures. For each of the three temperatures, the plots on the left show $\log_{10}$(frequency) versus scaled genotype entropy $\frac{S_n^\gamma}{\ell \log k}$ for each phenotype. In these plots, the DGP entropy line is also shown, as well as the maximum PrGP entropy line. The plots on the right show scaled genotype entropy $\frac{S_n^\gamma}{\ell \log k}$ versus robustness ρ_n for each phenotype. The line $\rho_n = \frac{S_n^\gamma}{\ell \log k}$ is also plotted. The PrGP genotype entropy tends to be lower than the corresponding DGP genotype entropy, but generally maintains the overall relationship between $S_n^\gamma \sim a + b \log f_n$. In the case of RNA GC20 at $70\,^\circ$C, we find that PrGP genotype entropy can exceed the DGP entropy but remains far from the maximum value. We also plot robustness versus the scaled genotype entropy and generally find a positive correlation, though with PrGPs typically having lower genotype entropy for the same robustness. At different temperatures (hence, disorder), we see behavior that is consistent with theory. Phenotype probability mass spreads spread over more genotypes as temperature increases, thus aquiring higher genotype entropy and straying farther away from the maximumally robust PrGP spread as derived in eq. (S74). In Figure S10, we similarly plot the genotype entropy S_n^γ for all phenoypes n for the RNA12 PrGP map.

In Figure S11 we plot the genotype entropy S_n^γ for all phenoypes n for each of the three spin glass PrGP map external field disorder settings for the main text spin glass trial. For each of the three external field disorder settings, the plots on the left show $\log_{10}$(frequency) versus scaled genotype entropy $\frac{S_n^\gamma}{\ell \log k}$ for each phenotype. In these plots, the DGP entropy line is also shown, as well as the maximum PrGP entropy line. The plots on the right show scaled genotype entropy $\frac{S_n^\gamma}{\ell \log k}$ versus robustness ρ_n for each phenotype. The line $\rho_n = \frac{S_n^\gamma}{\ell \log k}$ is also plotted. The PrGP genotype entropy tends to be lower than the corresponding DGP genotype entropy, but generally maintains the overall relationship between $S_n^\gamma \sim a + b \log f_n$. We also plot robustness versus the scaled genotype entropy and generally find a positive correlation, though with PrGPs typically having lower genotype entropy for the same robustness. At different external field disorder settings, we see behavior that is consistent with theory. Phenotype probability mass spreads spread over more genotypes as xternal field disorder increases, thus aquiring higher genotype entropy and straying farther away from the maximumally robust PrGP spread as derived in eq. (S74).

In Figure S12 we plot the genotype entropy S_n^γ for all phenoypes n for an 11-qubit quantum circuit PrGP map validation trial. We observe several groups of phenotypes behave linearly between log frequency and scaled genotype entropy. It is interesting to note that the genotype entropy from the noiseless quantum circuit fall exactly along the DGP genotype entropy line, while the noisy simulations show groups of phenotypes which each cross the same line, each with higher slopes than in eq. (S73).

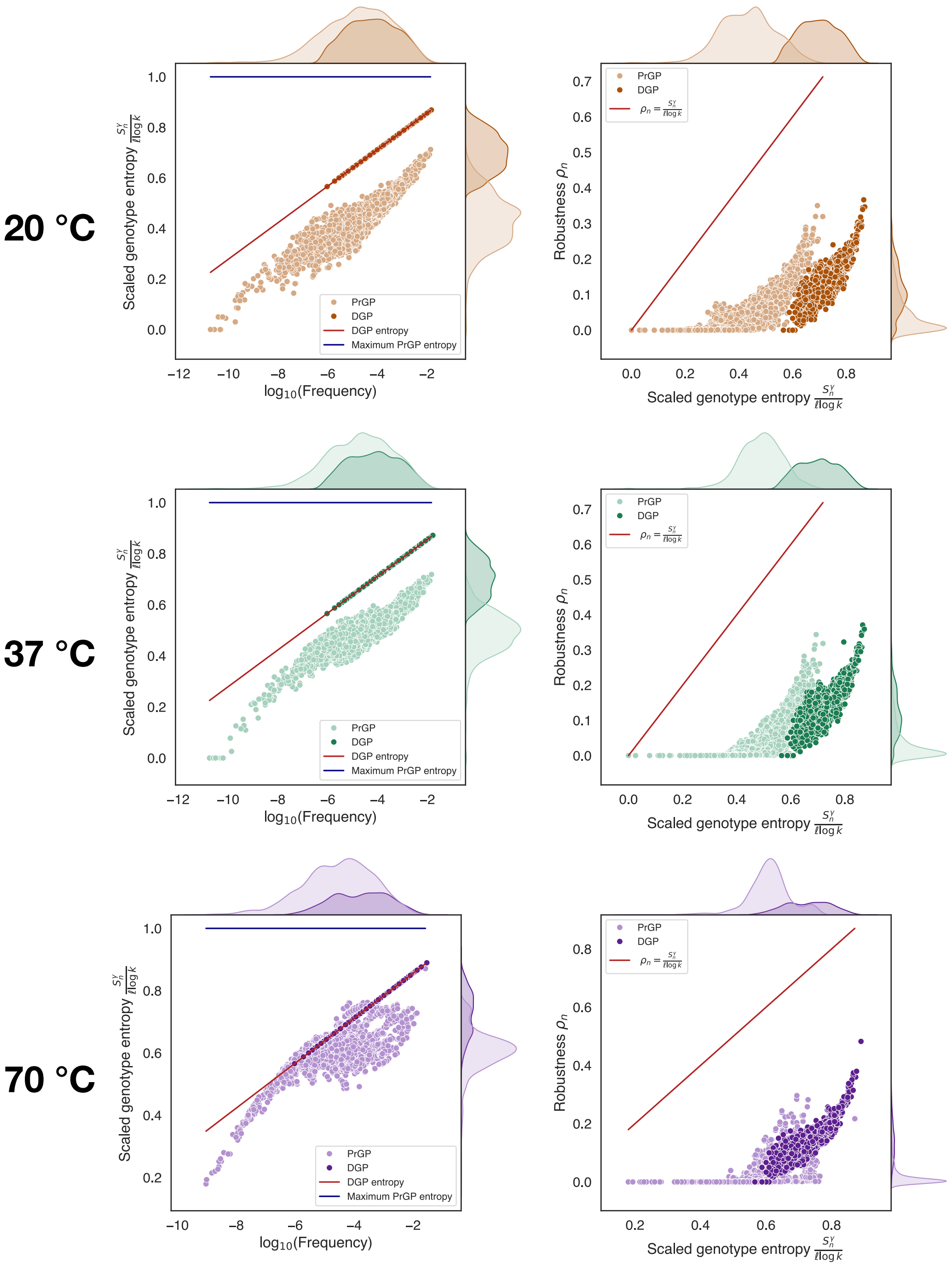

FIG. S9. Plots of (left) log$_{10}$(frequency) versus scaled genotype entropy $\frac{S_n^\gamma}{\ell \log k}$ and (right) scaled genotype entropy $\frac{S_n^\gamma}{\ell \log k}$ versus robustness ρ_n for RNA folding PrGP maps across three temperatures with $k = 2, \ell = 20$.

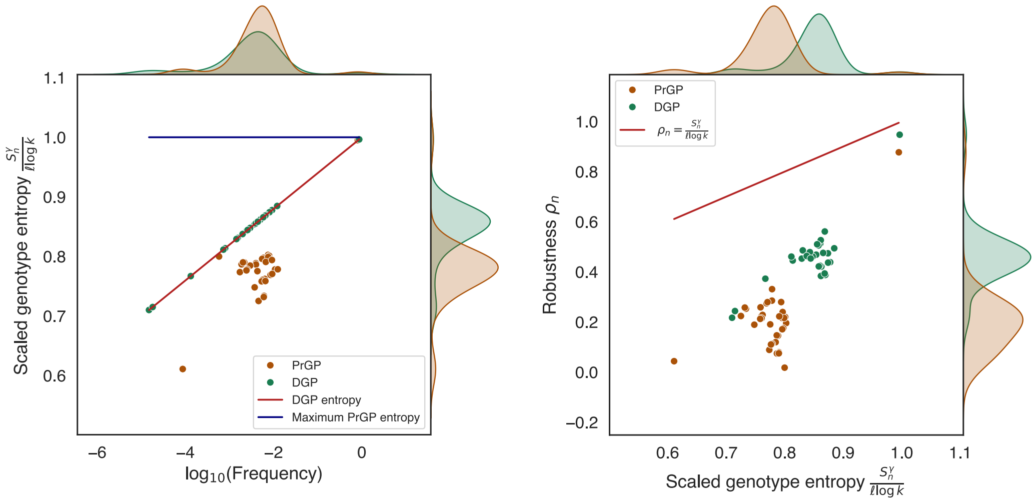

FIG. S10. Plots of (left) $\log_{10}$(frequency) versus scaled genotype entropy $\frac{S_n^\gamma}{\ell \log k}$ and (right) scaled genotype entropy $\frac{S_n^\gamma}{\ell \log k}$ versus robustness ρ_n for RNA folding PrGP maps with $k = 4, \ell = 12$.

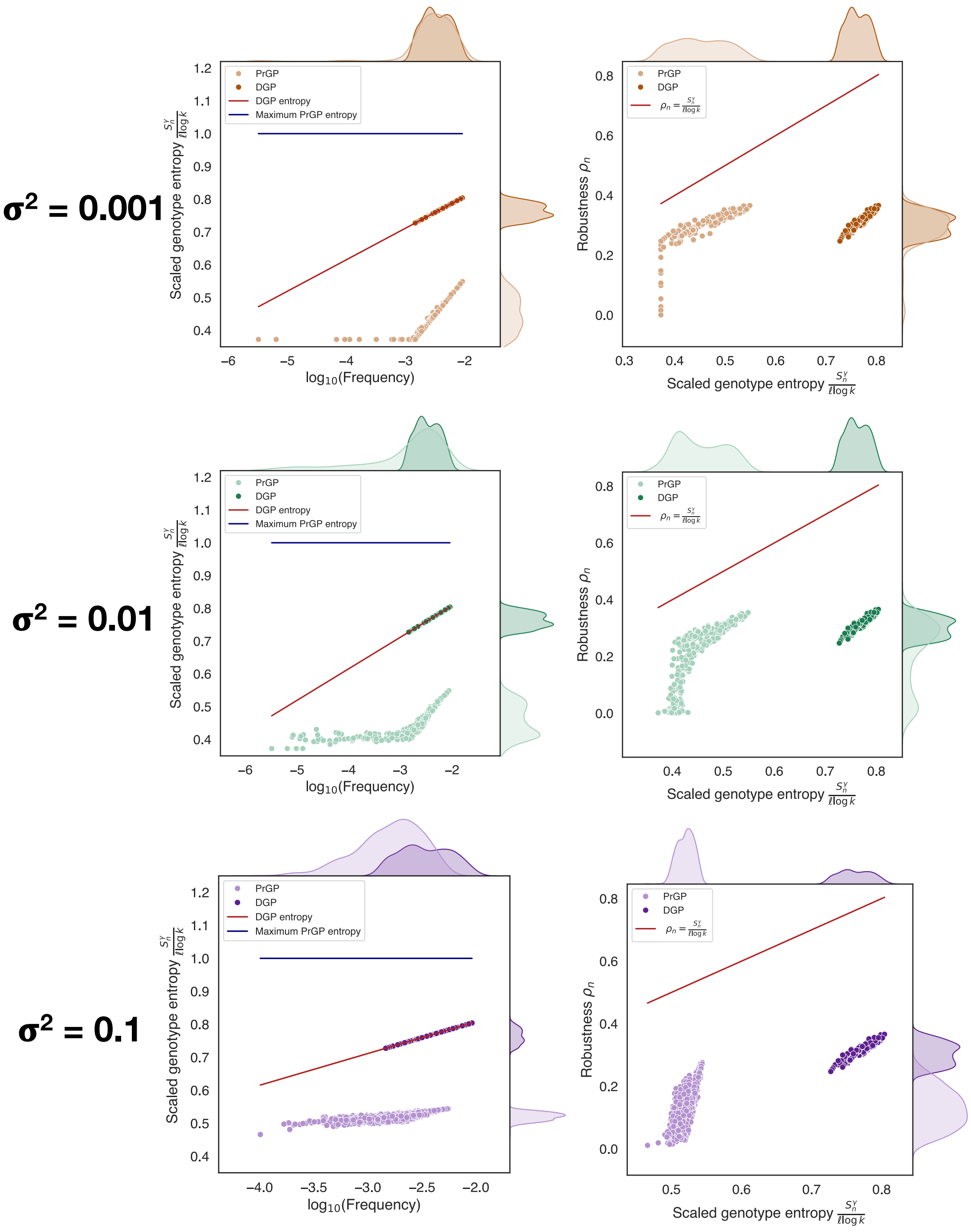

FIG. S11. Plots of (left) $\log_{10}$(frequency) versus scaled genotype entropy $\frac{S_n^\gamma}{\ell \log k}$ and (right) scaled genotype entropy $\frac{S_n^\gamma}{\ell \log k}$ versus robustness ρ_n for each spin glass ground state at three different external field variances for the spin glass PrGP maps.

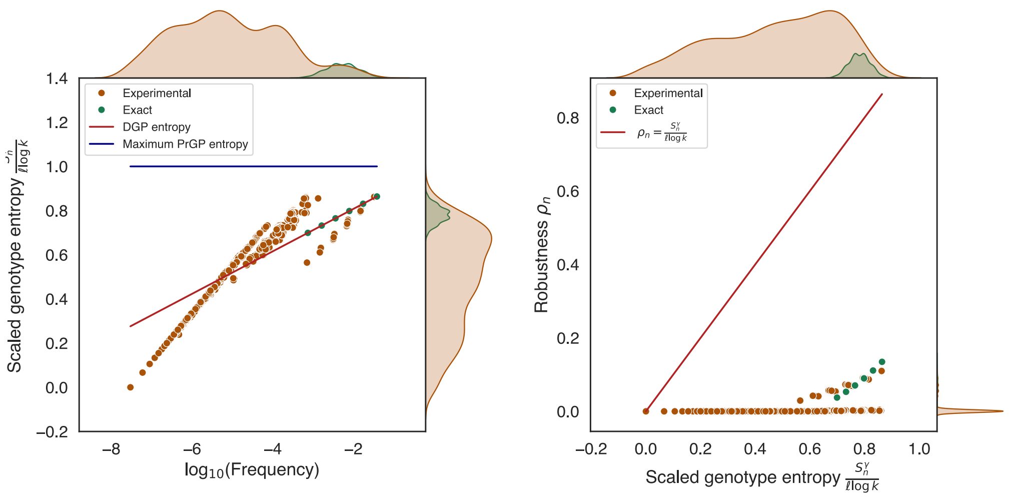

FIG. S12. Plots of (left) $\log_{10}$(frequency) versus scaled genotype entropy $\frac{S_n^\gamma}{\ell \log k}$ and (right) scaled genotype entropy $\frac{S_n^\gamma}{\ell \log k}$ versus robustness ρ_n for the simulated 11-qubit quantum circuit trial.

VII. EXTENDED DATA FOR MAIN TEXT RNA FOLDING PrGP MAP, GC ALPHABET, $\ell = 20, k = 2$

In the main text, we presented robustness versus frequency and robustness versus $\log_{10}$(frequency) plots for RNA folding PrGP and DGP maps at three temperatures. For clarity, we have included robustness versus frequency, robustness versus $\log_{10}$(frequency), and $\log_{10}$(robustness) versus $\log_{10}$(frequency) plots separately for PrGP and DGP maps in Figure S13. First, we see that the DGP map results reproduce the expected $\rho_n \propto \log f_n$ relationship for most phenotypes, with significant elevation above the random null model expectation. We also note that there is little temperature dependence in DGP robustness calculations, which suggests the effect of temperature does little to alter the exact ground state phenotype. In contrast, our PrGP map results showcase a different robustness behavior in which as simulation temperature increases, there is a gradual but clear suppression of the robustness versus frequency relationship; see main text for discussion of these features. In the PrGP map results we also note a biphasic behavior in which for high frequency phenotypes, the PrGP map robustness, similar to the DGP map robustness, is substantially elevated above the random null expectation and for lower frequencies, the robustness behaves more like the random model.

In Table S2, we include the Pearson correlation coefficient r and Spearman rank correlation coefficient ρ for each map (PrGP, DGP), temperature (20 °C, 37 °C, 70 °C), and axis transformation presented in Figure 2(a-b) and Figure S13. The primary feature we point out is the relative decrease of the PrGP Pearson r coefficients in robustness versus $\log_{10}$(frequency) plots as compared to the DGP plots; this suggests a deviation from the empirical $\rho_n \propto \log f_n$ trend observed in DGP studies.

In the GP map literature, *phenotype bias*, the finding that phenotype frequencies can vary over many orders of magnitude with a small number of phenotypes being the targets of a large number of genotypes, has been shown for many systems [3, 6–8]. In Figure S14, we present plots of $\log_{10}$(frequency) versus normalized rank and $\log_{10}$(frequency) versus $\log_{10}$(normalized rank) for each temperature and map pairing which show phenotype bias for this RNA folding system. Notably, the $\log_{10}$(frequency) versus $\log_{10}$(normalized rank) plot suggests a deviation from Zipf's law.

Figure S15 presents transition probabilities ϕ_{mn} for the most frequently occurring phenotype n to the other phenotypes m due to a single nucleotide mutation for both PrGP and DGP maps at three different temperatures. For each respective map, a plot including and excluding the most robust transition (*i.e.* from phenotype $n \to n$) is shown for added clarity. This figure demonstrates that the off-diagonal transition probabilities for PrGP maps maintained an approximate relationship $\phi_{mn} \propto f_m$ for $m \neq n$ in concordance with DGP maps, and in concordance with the random null expectation for PrGP maps (see main text). A proportionality constant not equal to 1 for $\phi_{mn} \propto f_m$ with $m \neq n$ is likely due to transition probability mass that is acquired by the diagonal element ϕ_{nn}. It is also apparent that the most robust transition is much more likely than the transition to any other phenotype, in support of our claim that PrGP maps, like DGP maps, exhibit enhanced robustness.

System	Alphabet, Length	Map	Temperature	Axes	Pearson r	Spearman ρ
RNA	GC, 20	PrGP	20 °C	Robust v. Freq	0.811	0.962
RNA	GC, 20	PrGP	20 °C	Robust v. $\log_{10}$(Freq)	0.813	0.962
RNA	GC, 20	PrGP	20 °C	$\log_{10}$(Robust) v. $\log_{10}$(Freq)	0.951	0.962
RNA	GC, 20	PrGP	37 °C	Robust v. Freq	0.854	0.974
RNA	GC, 20	PrGP	37 °C	Robust v. $\log_{10}$(Freq)	0.784	0.974
RNA	GC, 20	PrGP	37 °C	$\log_{10}$(Robust) v. $\log_{10}$(Freq)	0.970	0.974
RNA	GC, 20	PrGP	70 °C	Robust v. Freq	0.856	0.982
RNA	GC, 20	PrGP	70 °C	Robust v. $\log_{10}$(Freq)	0.665	0.982
RNA	GC, 20	PrGP	70 °C	$\log_{10}$(Robust) v. $\log_{10}$(Freq)	0.982	0.982
RNA	GC, 20	DGP	20 °C	Robust v. Freq	0.721	0.860
RNA	GC, 20	DGP	20 °C	Robust v. $\log_{10}$(Freq)	0.868	0.860
RNA	GC, 20	DGP	20 °C	$\log_{10}$(Robust) v. $\log_{10}$(Freq)	0.839	0.860
RNA	GC, 20	DGP	37 °C	Robust v. Freq	0.717	0.856
RNA	GC, 20	DGP	37 °C	Robust v. $\log_{10}$(Freq)	0.859	0.856
RNA	GC, 20	DGP	37 °C	$\log_{10}$(Robust) v. $\log_{10}$(Freq)	0.836	0.856
RNA	GC, 20	DGP	70 °C	Robust v. Freq	0.759	0.914
RNA	GC, 20	DGP	70 °C	Robust v. $\log_{10}$(Freq)	0.903	0.914
RNA	GC, 20	DGP	70 °C	$\log_{10}$(Robust) v. $\log_{10}$(Freq)	0.884	0.914

TABLE S2. Pearson and Spearman correlation coefficients for all robustness versus frequency plots in main text/Supplemental Material for RNA $k = 2, \ell = 20$ simulations with reduced alphabet, for each simulation temperature.

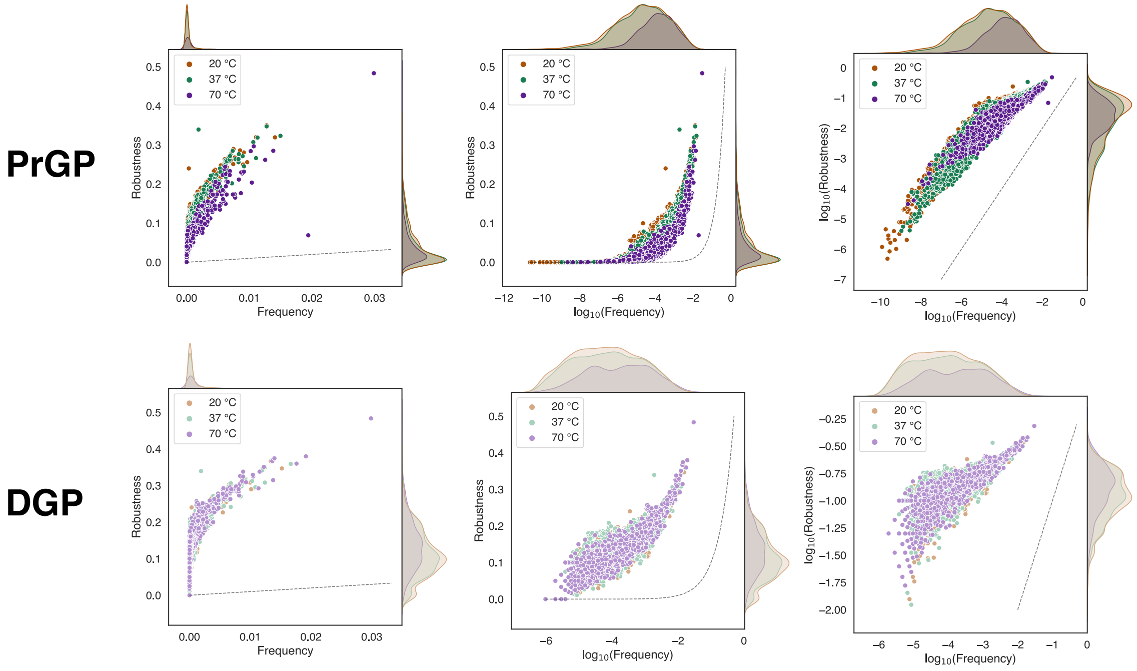

FIG. S13. Plots of (left) robustness versus frequency, (middle) robustness versus $\log_{10}$(frequency), and (right) $\log_{10}$(robustness) versus $\log_{10}$(frequency) for RNA folding (top row) PrGP maps and (bottom row) DGP maps for three temperatures. These data are the same results from main text Figure 2, with axis scaling adjusted and with PrGP and DGP data shown separately for clarity. The dashed line is the random null expectation for both PrGP and DGP maps given by $\phi_{mn} = f_m$ for all m and n.

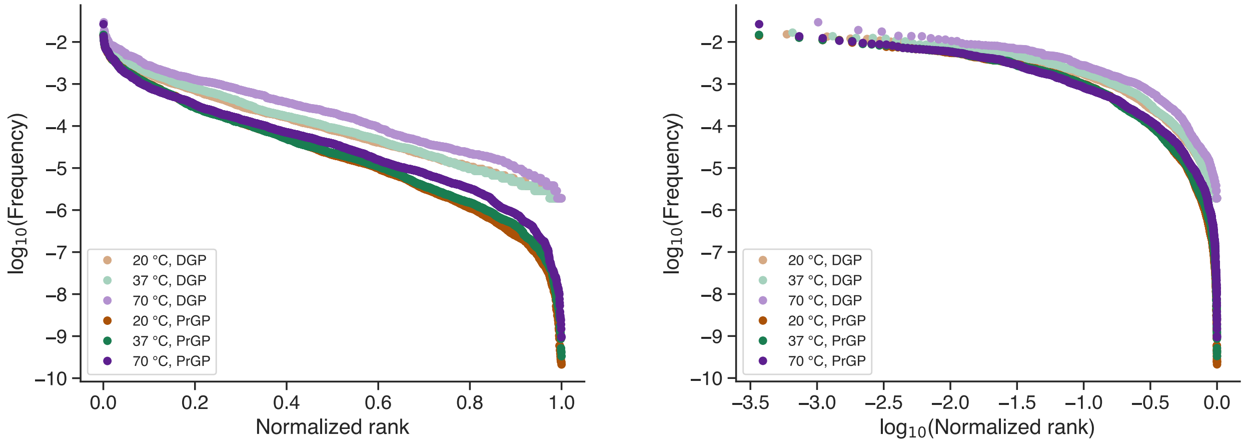

FIG. S14. Plots of (left) $\log_{10}$(frequency) versus normalized rank and (right) $\log_{10}$(frequency) versus $\log_{10}$(normalized rank) for RNA folding PrGP and DGP maps for three temperatures. When computing ranks, ties were broken arbitrarily.

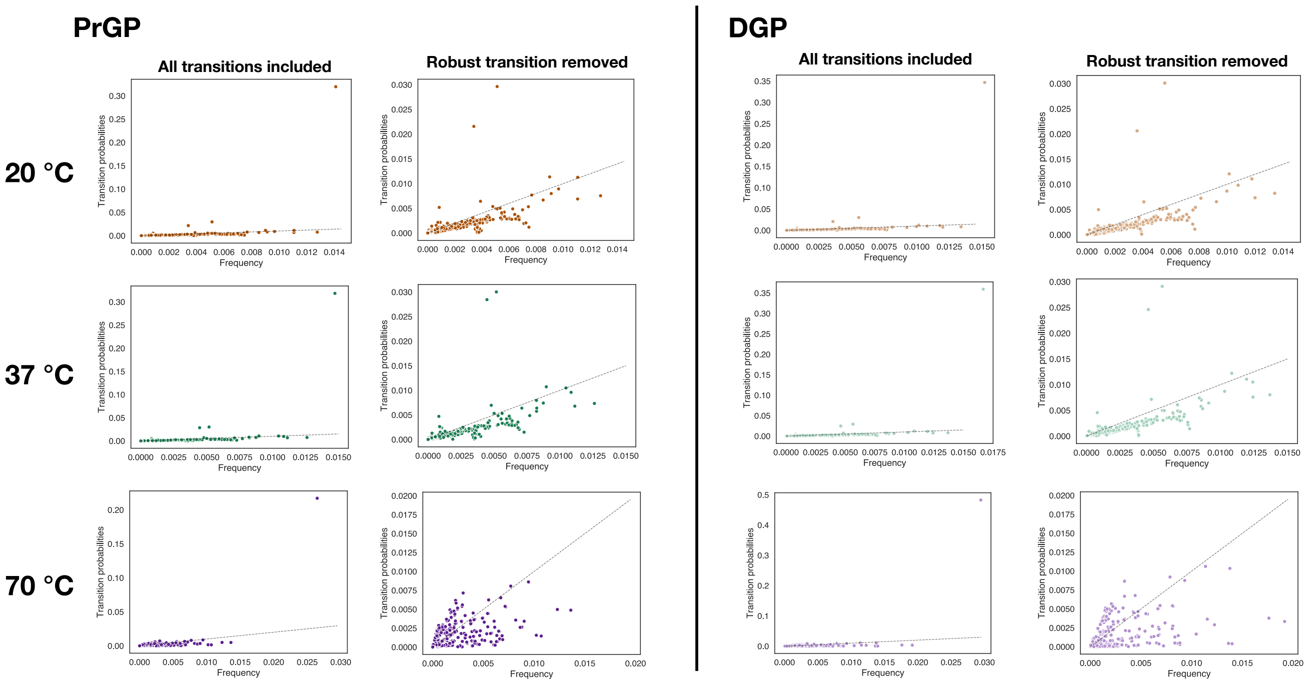

FIG. S15. Plots of transition probabilities versus frequency for RNA folding (left) PrGP maps and (right) DGP maps for three temperatures, (top) 20° C, (middle) 37° C, and (bottom) 70° C. For each respective map, plots include either (left) all transitions or (right) have the most robust transition removed. The dashed line is the random null expectation for both PrGP and DGP maps given by $\phi_{mn} = f_m$ for all m and n.

VIII. VALIDATION TRIAL FOR RNA FOLDING PrGP MAP, FULL ALPHABET, $\ell = 12, k = 4$

Here, we present results of a validation trial for RNA folding PrGP maps for sequences of length $\ell = 12$ utilizing the full alphabet of size $k = 4$, $\{\mathrm{A, C, G, U}\}$. In Figure S16, we present robustness versus frequency, robustness versus $\log_{10}$(frequency), and $\log_{10}$(robustness) versus $\log_{10}$(frequency) plots for RNA folding for PrGP and DGP maps. As with the reduced alphabet case, we see both PrGP and DGP map results show significant elevation above the random null model expectation, with PrGP map results demonstrating a gradual but clear suppression of the robustness versus frequency relationship compared to DGP map results. The expected $\rho_n \propto \log f_n$ relationship for phenotypes in the DGP map results as well as the biphasic behavior of the PrGP map results is present but less clear in this case, likely due to a small size effect from the limited number of phenotypes present in this complete alphabet ($k = 4$, $\ell = 12$) system compared to the reduced alphabet system ($k = 2$), which contains sequences of longer length ($\ell = 20$). Also in Figure S16, we plot the distribution of phenotype entropy $S(g)$ across all genotypes g; most phenotype entropies are zero due to their being deterministic because for the RNA folding, $k = 4$, $\ell = 12$ system most genotypes do not fold.

In Table S3, we include the Pearson correlation coefficient r and Spearman rank correlation coefficient ρ for each map (PrGP, DGP) and axis transformation presented in Figure S16. In Figure S17, we present plots of $\log_{10}$(frequency) versus normalized rank and $\log_{10}$(frequency) versus $\log_{10}$(normalized rank) for each temperature and map pairing which show phenotype bias for this RNA folding system. Notably, the $\log_{10}$(frequency) versus $\log_{10}$(normalized rank) plot suggests a deviation from Zipf's law.

Figure S18 presents transition probabilities ϕ_{mn} for the most frequently occurring phenotype n to the other phenotypes m due to a single nucleotide mutation for both PrGP and DGP maps. For each respective map, a plot including and excluding the most robust transition is shown for added clarity. This figure demonstrates that the off-diagonal transition probabilities for PrGP maps maintained an approximate relationship $\phi_{mn} \propto f_m$ for $m \neq n$ in concordance with DGP maps, and in concordance with the random null expectation for PrGP maps (see main text). A proportionality constant not equal to 1 for $\phi_{mn} \propto f_m$ with $m \neq n$ is likely due to transition probability mass that is acquired by the diagonal element ϕ_{nn}. It is also apparent that the most robust transition is much more likely than the transition to any other phenotype, in support of our claim that PrGP maps, like DGP maps, exhibit enhanced robustness.

System	Alphabet, Length	Map	Axes	Pearson r	Spearman ρ
RNA	AUCG, 12	PrGP	Robust v. Freq	0.832	0.927
RNA	AUCG, 12	PrGP	Robust v. $\log_{10}$(Freq)	0.882	0.927
RNA	AUCG, 12	PrGP	$\log_{10}$(Robust) v. $\log_{10}$(Freq)	0.877	0.927
RNA	AUCG, 12	DGP	Robust v. Freq	0.788	0.244
RNA	AUCG, 12	DGP	Robust v. $\log_{10}$(Freq)	0.814	0.244
RNA	AUCG, 12	DGP	$\log_{10}$(Robust) v. $\log_{10}$(Freq)	0.855	0.244

TABLE S3. Pearson and Spearman correlation coefficients for all robustness versus frequency plots for RNA $k = 4, \ell = 12$ validation trial with reduced alphabet. Simulations were conducted at 37 °C.

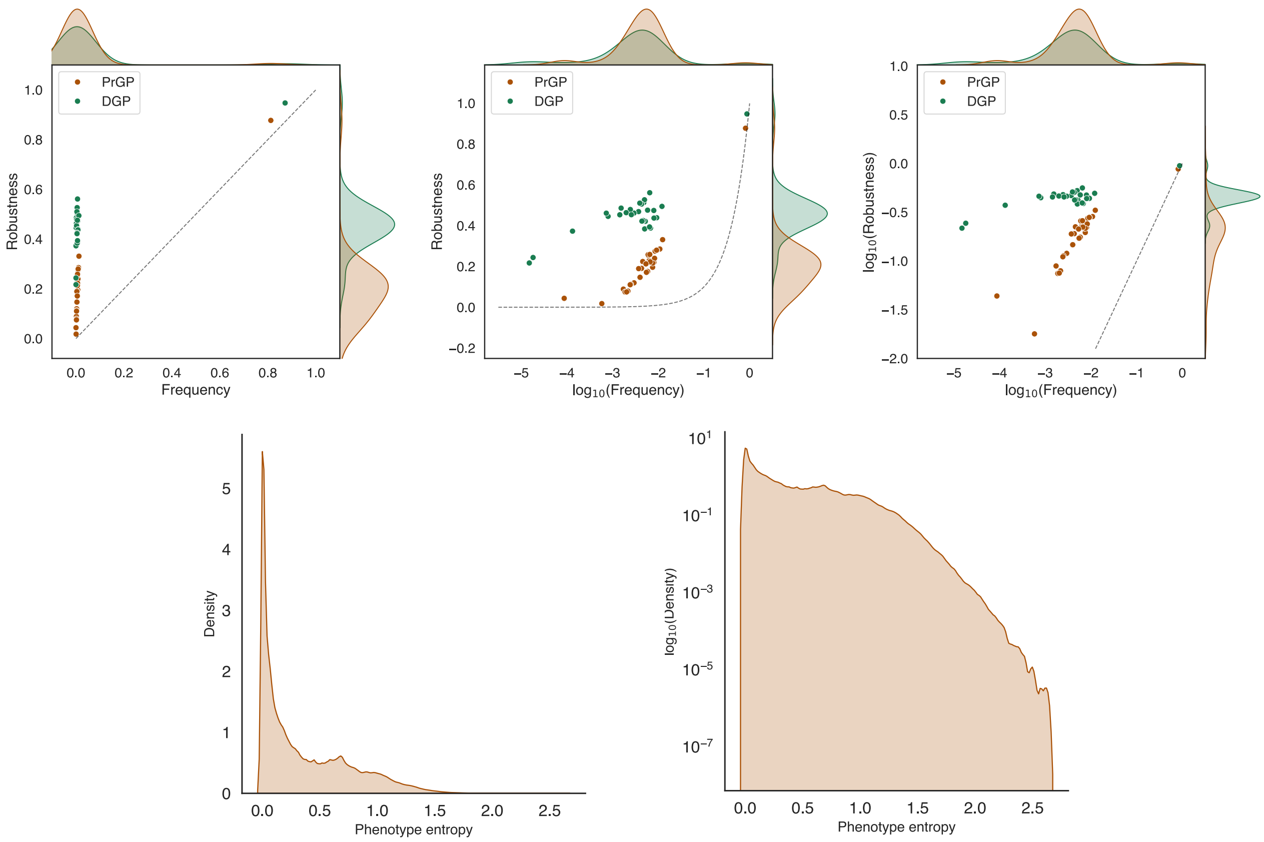

FIG. S16. Plots of (top left) robustness versus frequency, (top middle) robustness versus $\log_{10}$(frequency), and (top right) $\log_{10}$(robustness) versus $\log_{10}$(frequency) for RNA folding PrGP and DGP maps. Plots of (bottom left) density versus phenotype entropy and (bottom right) $\log_{10}$(density) versus phenotype entropy. The dashed line is the random null expectation for both PrGP and DGP maps given by $\phi_{mn} = f_m$ for all m and n.

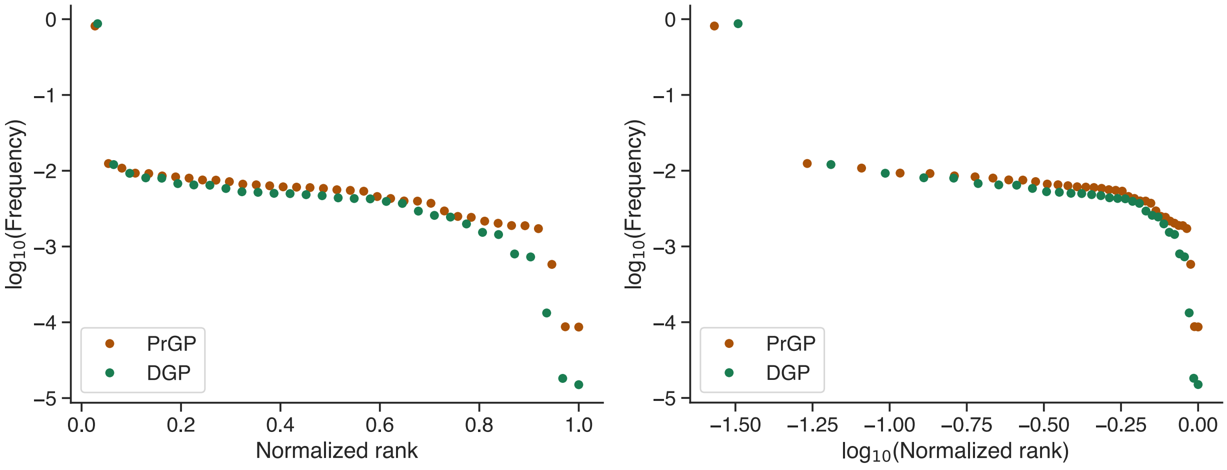

FIG. S17. Plots of (left) $\log_{10}$(frequency) versus normalized rank and (right) $\log_{10}$(frequency) versus $\log_{10}$(normalized frequency) for RNA folding PrGP and DGP maps. When computing ranks, ties were broken arbitrarily.

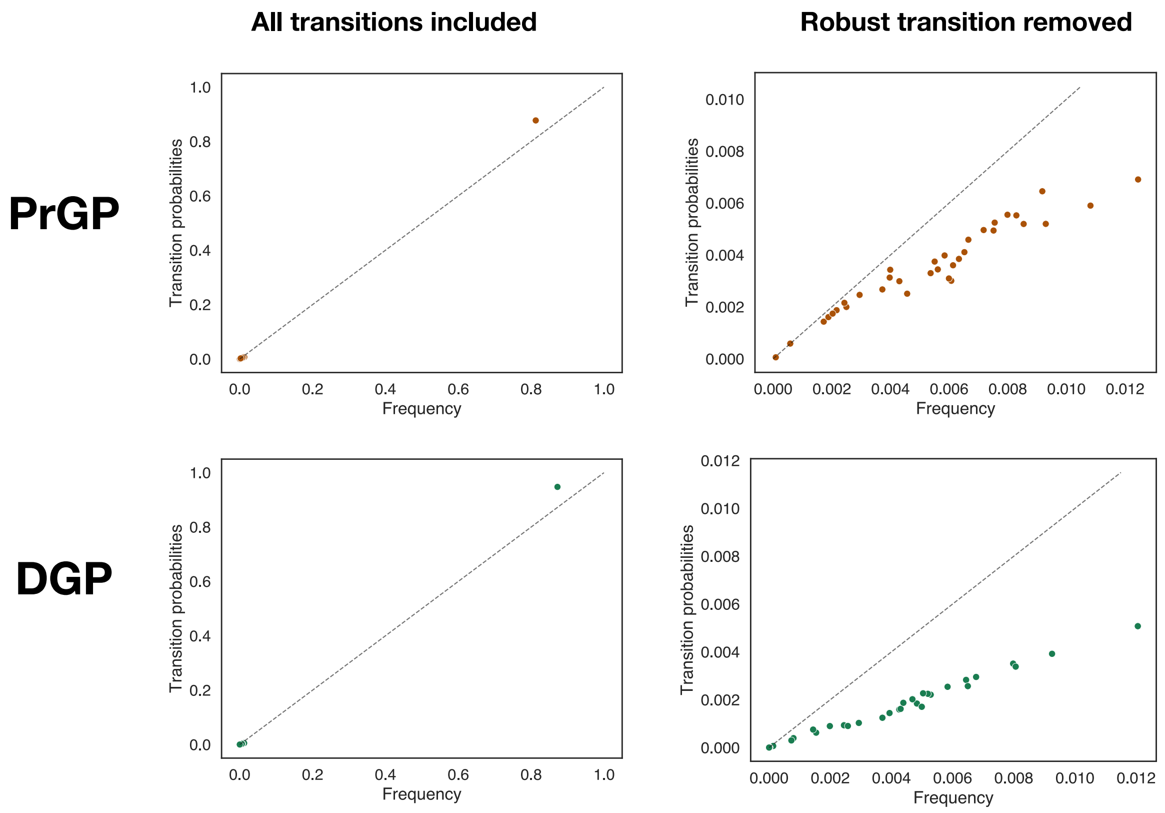

FIG. S18. Plots of transition probabilities versus frequency RNA folding (top) PrGP maps and (bottom) DGP maps. For each respective map, plots include either (left) all transitions or (right) have the most robust transition removed. The dashed line is the random null expectation for both PrGP and DGP maps given by $\phi_{mn} = f_m$ for all m and n.

IX. EXTENDED DATA FOR MAIN TEXT SPIN GLASS PrGP MAP

In the main text, we compared a spin glass DGP map with a fixed random external field $\{h_{0,i}\}$ with our spin glass PrGP map, which introduces a Gaussian distribution to the external field whose means are fixed at $\{h_{0,i}\}$ and whose variance σ_h^2 is varied as an independent variable. Figure S19 shows the topology of the graph $\mathcal{G}(V, E)$ (with $|V| = 9$, $|E| = 15$) that corresponds to the spin glass PrGP map data presented in the main text.

Main text Figure 2(c, d) presents robustness versus frequency and robustness versus $\log_{10}$(frequency) data; Figure S20 additionally plots $\log_{10}$(robustness) versus $\log_{10}$(frequency) for these same data. These three plots collectively demonstrate that, as with RNA folding GP maps, as the disorder parameter increases the uncertainty in the genotype-phenotype pairing, the robustness versus frequency relationship in PrGP maps becomes suppressed relative to the DGP map limit. These spin glass results are highly suggestive of a biphasic robustness relationship where at high frequencies, ρ_n is substantially enhanced above the random null expectation and behavior close to the deterministic limit is observed. However, as is clear from Figure S20, nearly linear behavior is observed for the smallest frequencies with the empirical robustness nearly parallel to the random expectation, suggesting $\rho_n \propto f_n$. See main text for discussion of these features.

In Table S4, we include the Pearson correlation coefficient r and Spearman rank correlation coefficient ρ for each map (PrGP, DGP), external field variance ($\sigma_h^2 = 0.001$, $\sigma_h^2 = 0.01$, $\sigma_h^2 = 0.1$), and axis transformation presented in Figure 2(c, d) and Figure S20. The primary feature we point out is the relative decrease of the PrGP Pearson r coefficients in robustness versus $\log_{10}$(frequency) plots as compared to the DGP (deterministic) plot; this suggests a deviation from the empirical $\rho_n \propto \log f_n$ trend observed in the spin glass DGP study [4].

In Figure S21, we present plots of $\log_{10}$(frequency) versus normalized rank and $\log_{10}$(frequency) versus $\log_{10}$(normalized rank) for each external field variance and the deterministic case. Notably, the $\log_{10}$(frequency) versus $\log_{10}$(normalized rank) plot suggests a deviation from Zipf's law.

Figure S22 presents transition probabilities ϕ_{mn} for the most frequently occurring ground state n to the other ground states m due to a single bond perturbation. For each setting of external random field variance, a plot including and excluding the most robust transition is shown for added clarity. This figure demonstrates that the off-diagonal transition probabilities for PrGP maps maintained an approximate relationship $\phi_{mn} \propto f_m$ for $m \neq n$ in concordance with DGP maps, and in concordance with the random null expectation for PrGP maps (see main text). A proportionality constant not equal to 1 for $\phi_{mn} \propto f_m$ with $m \neq n$ is likely due to transition probability mass that is acquired by the diagonal element ϕ_{nn}. It is also apparent that the most robust transition is much more likely than the transition to any other phenotype, in support of our claim that PrGP maps, like DGP maps, exhibit enhanced robustness.

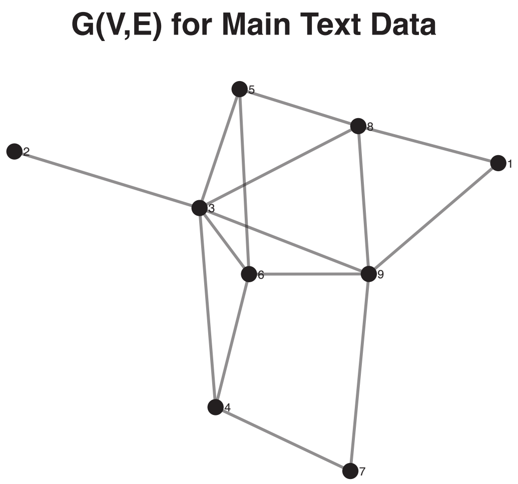

FIG. S19. Graph $\mathcal{G}(V, E)$ corresponding to the spin glass PrGP map data presented in the main text.

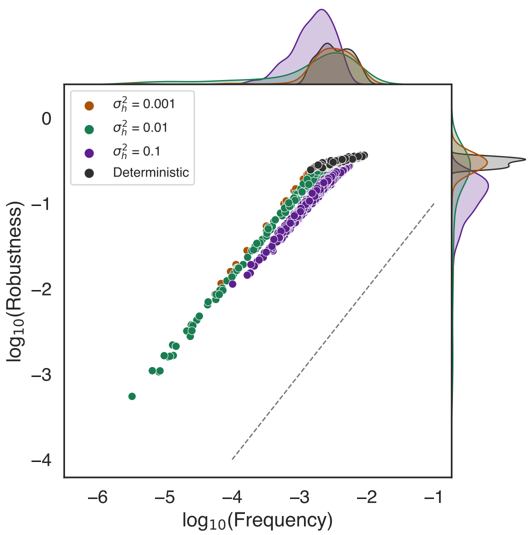

FIG. S20. Plot of $\log_{10}$(robustness) versus $\log_{10}$(frequency) for each spin glass ground state at three different external field variances for the spin glass PrGP maps. The DGP map limiting case is also plotted for comparison. This is the same as the main text plots but with axes scaled as log-log. The dashed line is the random null expectation for both PrGP and DGP maps given by $\phi_{mn} = f_m$ for all m and n.

**System**	**Map**	σ_h^2	**Axes**	**Pearson** r	**Spearman** ρ
Spin glass	PrGP	0.001	Robust v. Freq	0.766	0.962
Spin glass	PrGP	0.001	Robust v. $\log_{10}$(Freq)	0.940	0.962
Spin glass	PrGP	0.001	$\log_{10}$(Robust) v. $\log_{10}$(Freq)	0.920	0.962
Spin glass	PrGP	0.01	Robust v. Freq	0.874	0.985
Spin glass	PrGP	0.01	Robust v. $\log_{10}$(Freq)	0.924	0.985
Spin glass	PrGP	0.01	$\log_{10}$(Robust) v. $\log_{10}$(Freq)	0.986	0.985
Spin glass	PrGP	0.1	Robust v. Freq	0.976	0.987
Spin glass	PrGP	0.1	Robust v. $\log_{10}$(Freq)	0.954	0.987
Spin glass	PrGP	0.1	$\log_{10}$(Robust) v. $\log_{10}$(Freq)	0.989	0.987
Spin glass	DGP	Deterministic	Robust v. Freq	0.930	0.962
Spin glass	DGP	Deterministic	Robust v. $\log_{10}$(Freq)	0.964	0.962
Spin glass	DGP	Deterministic	$\log_{10}$(Robust) v. $\log_{10}$(Freq)	0.962	0.962

TABLE S4. Pearson and Spearman correlation coefficients for all robustness versus frequency plots for the spin glass PrGP map with $|V| = 9$ and $|E| = 15$ whose data are shown in the main text and here in the Supplemental Material.

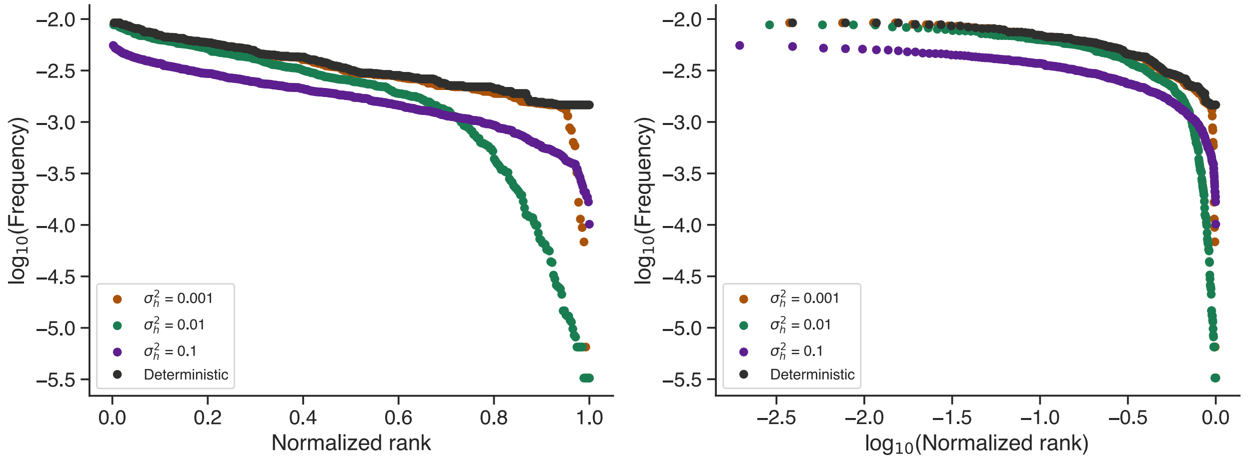

FIG. S21. Plots of (left) $\log_{10}$(frequency) versus normalized rank and (right) $\log_{10}$(frequency) versus $\log_{10}$(normalized frequency) for spin glass ground states for PrGP maps at three external field variances and DGP maps the deterministic case. When computing ranks, ties were broken arbitrarily.

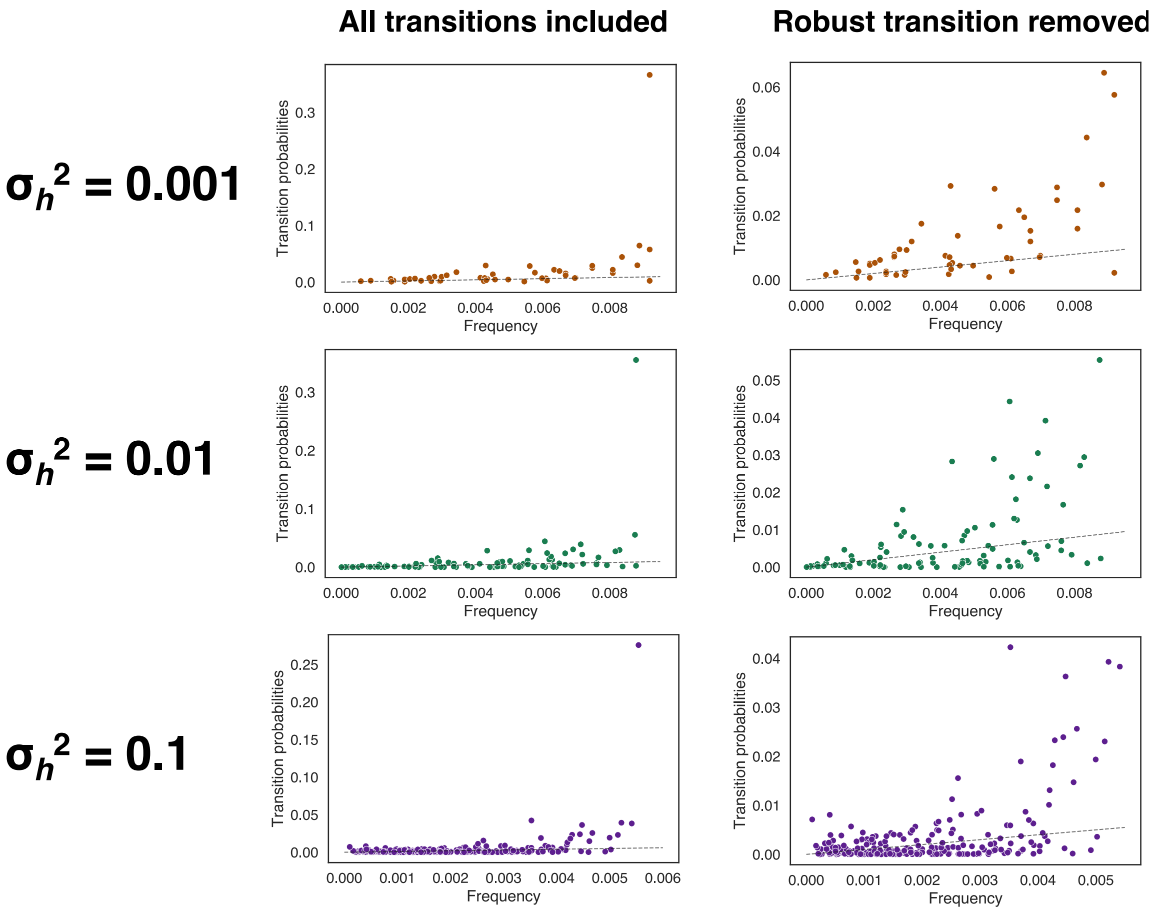

FIG. S22. Plots of transition probabilities versus frequency for spin glass ground states for PrGP maps at three external field variances, (top) $\sigma_h^2 = 0.001$, (middle) $\sigma_h^2 = 0.01$, and (bottom) $\sigma_h^2 = 0.1$. For each, plots include either (left) all transitions or (right) have the most robust transition removed. The dashed line is the random null expectation for both PrGP and DGP maps given by $\phi_{mn} = f_m$ for all m and n.

X. VALIDATION TRIAL FOR SPIN GLASS PrGP MAP

We provide a second spin glass PrGP map trial here in the Supplemental Material to illustrate that the spin glass trends described above and in the main text hold across multiple random graph instances. We generate a new $\mathcal{G}(V, E)$, once again with $|V| = 9$, $|E| = 15$ with topology shown in Figure S23. Figure S24 presents robustness versus frequency, robustness versus $\log_{10}$(frequency), and $\log_{10}$(robustness) versus $\log_{10}$(frequency) for spin glass PrGP maps at three different external field variances and for the deterministic case for DGP maps. The results from this validation trial exhibit the same behavior as observed in the trial presented in the main text. In particular, we see that as the disorder parameter increases the uncertainty in the genotype-phenotype pairing, the robustness versus frequency relationship in PrGP maps becomes suppressed relative to the DGP map limit. Again, these spin glass results are highly suggestive of a biphasic robustness relationship where at high frequencies, ρ_n is substantially enhanced above the random null expectation and behavior close to the deterministic limit is observed. However, as is clear from Figure S20, nearly linear behavior is observed for the smallest frequencies with the empirical robustness nearly parallel to the random expectation, signaling $\rho_n \propto f_n$. See main text for discussion of these features. Additionally, Figure S24 plots the distribution of phenotype entropy $S(g)$ across all genotypes g for PrGP maps at each external field variance experimental value. As is the case in Figure S8, we observe that the entropy distributions shift rightward as the disorder parameter increases.

In Table S5, we include the Pearson correlation coefficient r and Spearman rank correlation coefficient ρ for each map (PrGP, DGP), external field variance ($\sigma_h^2 = 0.001$, $\sigma_h^2 = 0.00$, $\sigma_h^2 = 0.1$), and axis transformation presented in Figure S24. The primary feature we point out is the relative decrease of the PrGP Pearson r coefficients in robustness versus $\log_{10}$(frequency) plots as compared to the DGP (deterministic) plot; this suggests a deviation from the empirical $\rho_n \propto \log f_n$ trend observed in the spin glass DGP study [4].

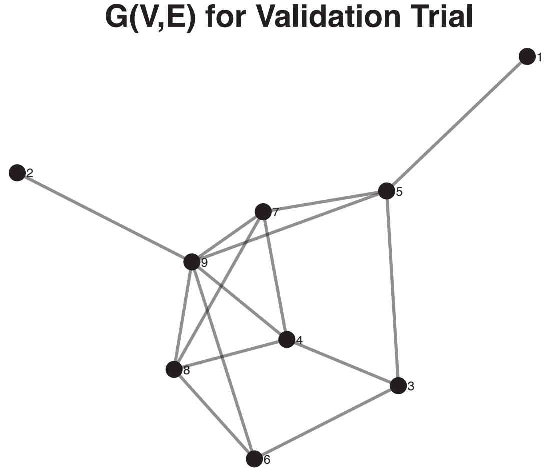

FIG. S23. Graph $\mathcal{G}(V, E)$ corresponding to the spin glass PrGP map validation trial data shown here in the Supplemental Material.

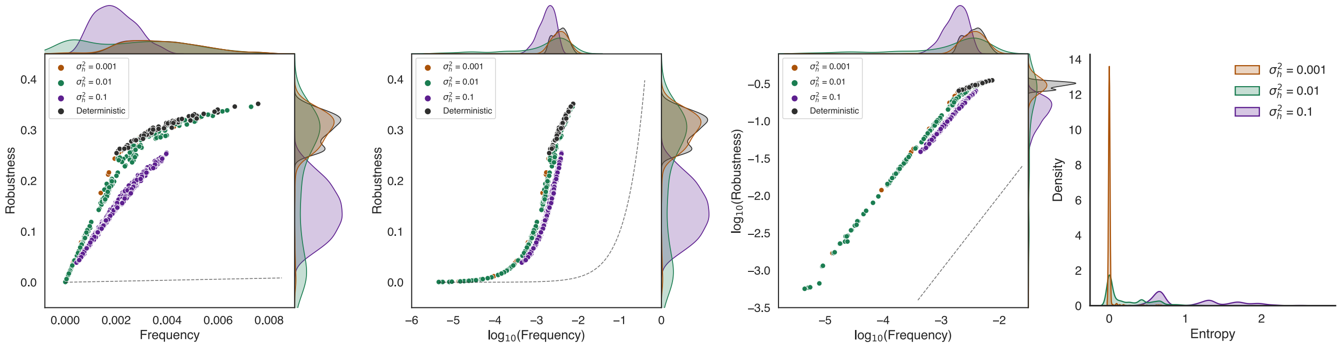

FIG. S24. Plots of (leftmost) robustness versus frequency, (middle left) robustness versus $\log_{10}$(frequency), and (middle right) $\log_{10}$(robustness) versus $\log_{10}$(frequency) for spin glass ground states for PrGP maps at three different external field variances and for the deterministic case for DGP maps. Additionally, the (rightmost) density versus phenotype entropy for the spin glass ground states at three difference external field variances is plotted. The dashed line is the random null expectation for both PrGP and DGP maps given by $\phi_{mn} = f_m$ for all m and n.

System	Map	σ_h^2	Axes	Pearson r	Spearman ρ
Spin glass	PrGP	0.001	Robust v. Freq	0.806	0.994
Spin glass	PrGP	0.001	Robust v. $\log_{10}$(Freq)	0.940	0.994
Spin glass	PrGP	0.001	$\log_{10}$(Robust) v. $\log_{10}$(Freq)	0.950	0.994
Spin glass	PrGP	0.01	Robust v. Freq	0.932	0.996
Spin glass	PrGP	0.01	Robust v. $\log_{10}$(Freq)	0.916	0.996
Spin glass	PrGP	0.01	$\log_{10}$(Robust) v. $\log_{10}$(Freq)	0.993	0.996
Spin glass	PrGP	0.1	Robust v. Freq	0.993	0.997
Spin glass	PrGP	0.1	Robust v. $\log_{10}$(Freq)	0.981	0.997
Spin glass	PrGP	0.1	$\log_{10}$(Robust) v. $\log_{10}$(Freq)	0.997	0.997
Spin glass	DGP	Deterministic	Robust v. Freq	0.962	0.995
Spin glass	DGP	Deterministic	Robust v. $\log_{10}$(Freq)	0.993	0.995
Spin glass	DGP	Deterministic	$\log_{10}$(Robust) v. $\log_{10}$(Freq)	0.990	0.995

TABLE S5. Pearson and Spearman correlation coefficients for all robustness versus frequency plots for the spin glass PrGP map validation trial with $|V| = 9$ and $|E| = 15$ whose data are shown above in this section.

XI. QUANTUM CIRCUIT GENERATION ALGORITHM

In this study, we generated quantum circuits with 7 qubits and 4 layers. We take the genotype of the quantum circuit PrGP map to be a subset of single qubit gates (which are varied to reflect each genotype). We first start by seeding the circuit randomly with $CNOT$ gates which cannot participate in the genotype gate list. Only certain pairs of qubits which are physically connected in the 7-qubit *ibm_lagos* v1.2.0 quantum computer can participate in the same $CNOT$ gate. The remaining open places are seeded with single qubit gates, and we choose $\ell = 4$ of these gates to be the variable gates for the genotype. The alphabet chosen is of size $k = 8$: $\{Z, X, Y, H, S, S^{\dagger}, T, T^{\dagger}\}$. Circuit diagrams used in our experimental trials are shown in the subsequent sections.

XII. EXTENDED DATA FOR MAIN TEXT QUANTUM CIRCUIT PrGP MAP

To our knowledge, this work is the first to analyze the structural properties of quantum circuit GP maps. We generate random quantum circuits as described in the main text and in the previous section with 7 qubits and 4 layers of gates. Figure S25 shows a schematic representation of the random quantum circuit generated for the quantum circuit PrGP map data presented in main text Figure 2(e, f) and in this Supplemental Material section.

Main text Figure 2(e, f) presents robustness versus frequency and robustness versus $\log_{10}$(frequency) data using both exact and experimental phenotype probability vectors for robustness calculations; Figure S26 additionally plots $\log_{10}$(robustness) versus $\log_{10}$(frequency) for these same data. Collectively, we see that for the exact probabilities, the results strongly support the enhanced $\rho_n \propto \log f_n$ scaling. The spread of phenotypes observed in the frequency domain is due to superposition and/or entanglement and many of the phentoypes are degenerate with identical frequency and robustness. This degeneracy is broken in our experimental measurements, which also exhibit measurement noise. Moreover, the frequency and robustness of these logarithmically scaling phenotypes is suppressed relative to the exact case as probability mass is drawn towards additional phenotypes which are observed experimentally which were not observed in the exact case. These quantum circuit PrGP map results are perhaps most illustrative of our suggested biphasic robustness scaling. The low frequency phenotypes which are introduced due to measurement noise in the experimental trials lie much closer to the random null expectation than the higher frequency phenotypes observed in the exact calculations, which rather scale with enhanced robustness similar to what is seen in standard DGP maps.

In Table S6, we include the Pearson correlation coefficient r and Spearman rank correlation coefficient ρ for both exact and experimental quantum circuit PrGP results for each axis transformation presented in main text Figure 2(e, f) and Figure S26. The primary features we point out are the high Perason correlation $r = 0.998$ of the robustness versus $\log_{10}$(frequency) relationship for the exact phenotype probability vectors, and the relative decrease of the experimental Pearson r coefficients in robustness versus $\log_{10}$(frequency) plot as compared to the exact plot. This suggests that the exact relationship exhibits behavior similar to the empirical $\rho_n \propto \log f_n$ trend observed in DGP studies, and that the experimental trials introduce measurement noise which induces a deviation from the exact results.

In Figure S27, we present plots of $\log_{10}$(frequency) versus normalized rank and of $\log_{10}$(frequency) versus $\log_{10}$(normalized rank) for experimental and exact quantum circuit PrGP map results. Notably, the plot showing $\log_{10}$(frequency) versus $\log_{10}$(normalized rank) suggests a deviation from Zipf's law.

Figure S28 presents transition probabilities ϕ_{mn} for the most frequently occurring circuit output state n to the other circuit output states m due to a single variable gate perturbation. For both experimental and exact phenotype probability vectors, a plot including and excluding the most robust transition is shown for added clarity. This figure demonstrates that the off-diagonal transition probabilities for quantum circuit PrGP maps are positively correlated with the frequency f_m, though there appears to be some additional nonrandom relationship which is not predicted from standard DGP or PrGP theory. It is also apparent that the most robust transition is much more likely than the transition to any other phenotype, in support of our claim that PrGP maps, like DGP maps, exhibit enhanced robustness.

System	Map	Trial	Exact or Exp	Axes	Pearson r	Spearman ρ
Quantum circuit	PrGP	1	Exact	Robust v. Freq	0.926	0.996
Quantum circuit	PrGP	1	Exact	Robust v. $\log_{10}$(Freq)	0.998	0.996
Quantum circuit	PrGP	1	Exact	$\log_{10}$(Robust) v. $\log_{10}$(Freq)	0.993	0.996
Quantum circuit	PrGP	1	Experimental	Robust v. Freq	0.912	0.987
Quantum circuit	PrGP	1	Experimental	Robust v. $\log_{10}$(Freq)	0.712	0.987
Quantum circuit	PrGP	1	Experimental	$\log_{10}$(Robust) v. $\log_{10}$(Freq)	0.983	0.987

TABLE S6. Pearson and Spearman correlation coefficients for all robustness versus frequency plots quantum circuit PrGP map whose robustness data was presented in the main text and in the above log-log plot. This includes both exact results as well as experimental results for realization/Trial 1, whose circuit is also printed earlier in this section.

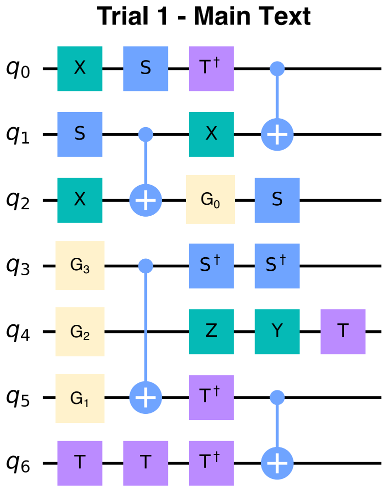

FIG. S25. Random circuit generated for quantum circuit trial 1, whose robustness data is plotted in the main text and below in the remainder of this section. The genotype is the set of variable gates $g = (G_0, G_1, G_2, G_3)$, so the length of the input sequence is $\ell = 4$ drawn from an alphabet of $k = 8$ single qubit gates: $\{Z, X, Y, H, S, S^\dagger, T, T^\dagger\}$.

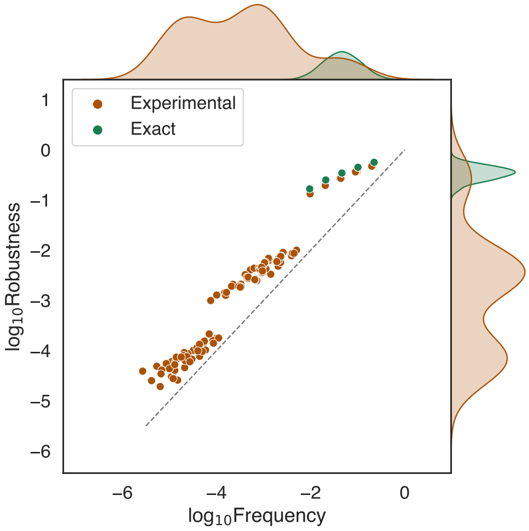

FIG. S26. Plot of $\log_{10}$(robustness) versus $\log_{10}$(frequency) for the quantum circuit in trial 1 for experimental and exact data. The dashed line is the random null expectation for both PrGP and DGP maps given by $\phi_{mn} = f_m$ for all m and n.

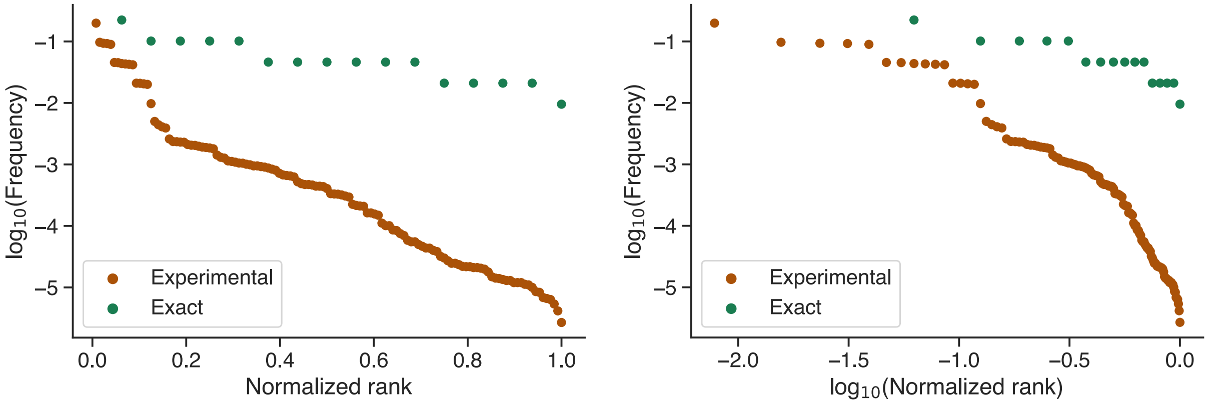

FIG. S27. Plots of (left) $\log_{10}$(frequency) versus normalized rank and (right) $\log_{10}$(frequency) versus $\log_{10}$(normalized frequency) for the quantum circuit trial 1 for experimental and exact data. The dashed line is the random null expectation for both PrGP and DGP maps given by $\phi_{mn} = f_m$ for all m and n.

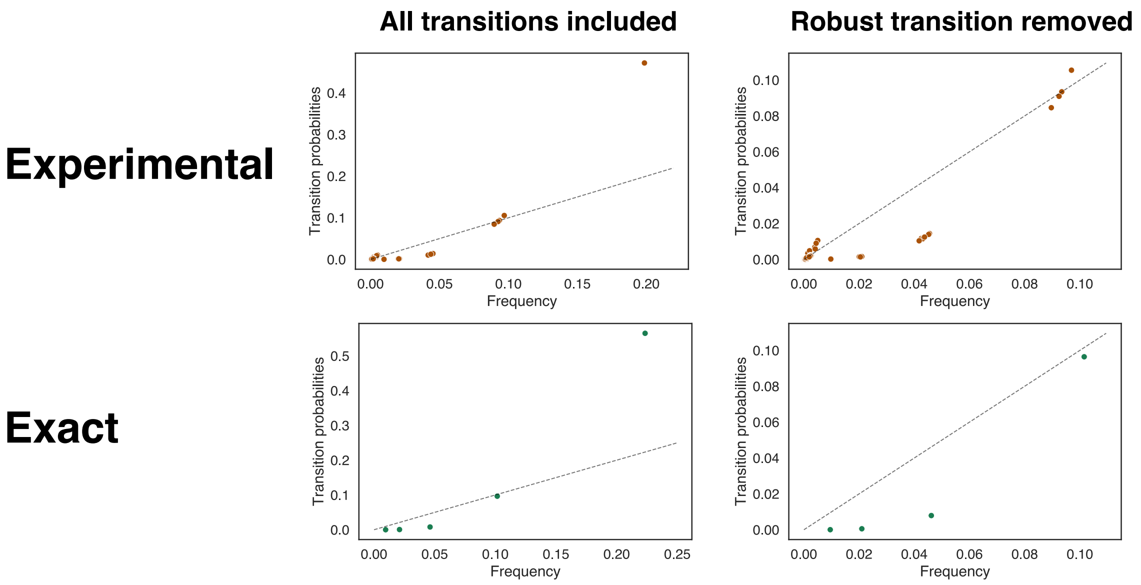

FIG. S28. Plots of (top) transition probabilities versus frequency for quantum circuit trial 1 for experimental and (bottom) exact data. For each model framework, plots include either (left) all transitions or (right) have the most robust transition removed. The dashed line is the random null expectation for both PrGP and DGP maps given by $\phi_{mn} = f_m$ for all m and n. The dashed line is the random null expectation for both PrGP and DGP maps given by $\phi_{mn} = f_m$ for all m and n.

XIII. 7 QUBIT VALIDATION TRIALS FOR QUANTUM CIRCUIT PrGP MAP

To validate the quantum circuit PrGP map results presented in the main text and Supplemental Material, six additional trials were conducted. A schematic of the random quantum circuit generated for the first of these validation trials is shown in Figure S29. Figure S30 presents robustness versus frequency, robustness versus $\log_{10}$(frequency), and $\log_{10}$(robustness) versus $\log_{10}$(frequency) for this quantum circuit PrGP map validation trial. As with the first quantum circuit PrGP map trial, these data strongly support the enhanced $\rho_n \propto \log f_n$ scaling. Again, we see the spread of phenotypes observed in the frequency domain due to superposition and/or entanglement and that many of the phentoypes are degenerate with identical frequency and robustness. This degeneracy is broken in our experimental measurements, which exhibit measurement noise. Once again, the frequency and robustness of these logarithmically scaling phenotypes is suppressed relative to the exact case as probability mass is drawn towards additional phenotypes which are observed experimentally which were not observed in the exact case. These results illustrate our suggested biphasic robustness scaling in which the low frequency phenotypes, which are introduced due to measurement noise in the experimental trials, lie much closer to the random null expectation than the higher frequency phenotypes observed in the exact calculations, which rather scale with enhanced robustness similar to what is seen in standard DGP maps. Figure S30 also presents a plot of the distribution of phenotype entropy $S(g)$ across all genotypes g for exact and experimental quantum circuit PrGP maps. Notably, the experimental entropy distribution is shifted rightward relative to the exact result due to measurement noise as well as a finite number of experimental trials.

In Table S7, we include the Pearson correlation coefficient r and Spearman rank correlation coefficient ρ for both exact and experimental quantum circuit PrGP results for each axis transformation presented in Figure S30. The primary features we point out are the high Perason correlation $r = 0.950$ of the robustness versus $\log_{10}$(frequency) relationship for the exact phenotype probability vectors, and the relative decrease of the experimental Pearson r coefficients in robustness versus $\log_{10}$(frequency) plot as compared to the exact plot. This suggests that the exact relationship exhibits behavior similar to the empirical $\rho_n \propto \log f_n$ trend observed in DGP studies, and that the experimental trials introduce measurement noise which induces a deviation from the exact results.

Figure S31 presents robustness versus frequency and robustness versus $\log_{10}$(frequency) plots as well as schematics of the corresponding random quantum circuits for validation trials 3-7. In each trial, the suggested biphasic robustness scaling is clear. Additionally, these trials support the enhanced $\rho_n \propto \log f_n$ scaling.

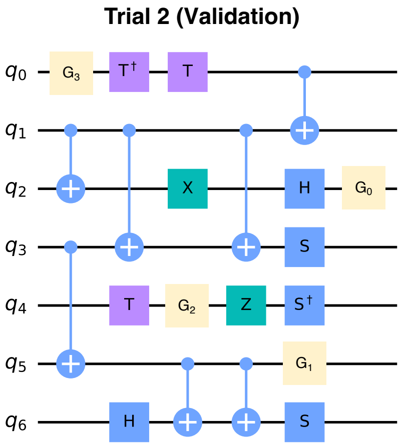

FIG. S29. Random circuit generated for quantum circuit trial 2, whose robustness and entropy data are plotted below as a validation trial. The genotype is the set of variable gates $g = (G_0, G_1, G_2, G_3)$, so the length of the input sequence is $\ell = 4$ drawn from an alphabet of $k = 8$ single qubit gates: $\{Z, X, Y, H, S, S^\dagger, T, T^\dagger\}$.

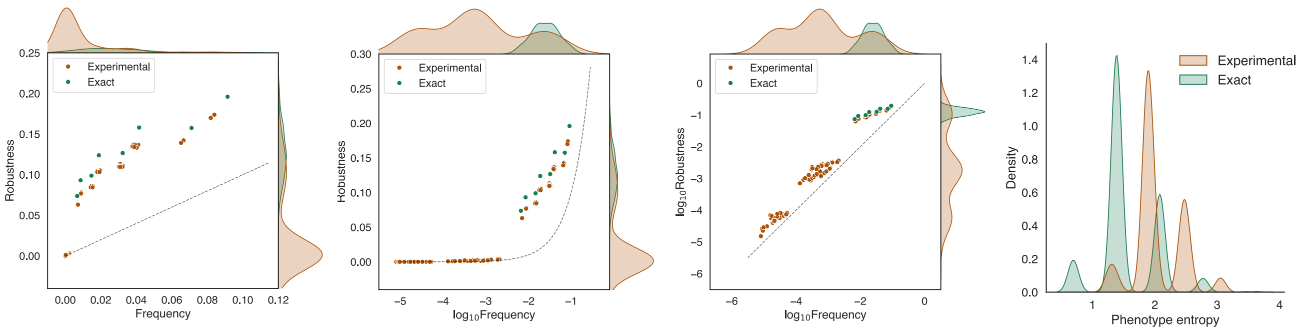

FIG. S30. Plot of (leftmost) robustness versus frequency, (left middle) robustness versus $\log_{10}$(frequency), and (middle right) $\log_{10}$(robustness) versus $\log_{10}$(frequency) for quantum circuit trial 2 for experimental and exact data. Additionally, the (rightmost) density versus phenotype entropy for quantum circuit trial 2 is plotted. The dashed line is the random null expectation for both PrGP and DGP maps given by $\phi_{mn} = f_m$ for all m and n.

**System**	**Map**	**Trial**	**Exact or Exp**	**Axes**	**Pearson r**	**Spearman ρ**
Quantum circuit	PrGP	2	Exact	Robust v. Freq	0.910	0.973
Quantum circuit	PrGP	2	Exact	Robust v. $\log_{10}$(Freq)	0.950	0.973
Quantum circuit	PrGP	2	Exact	$\log_{10}$(Robust) v. $\log_{10}$(Freq)	0.954	0.973
Quantum circuit	PrGP	2	Experimental	Robust v. Freq	0.916	0.979
Quantum circuit	PrGP	2	Experimental	Robust v. $\log_{10}$(Freq)	0.837	0.979
Quantum circuit	PrGP	2	Experimental	$\log_{10}$(Robust) v. $\log_{10}$(Freq)	0.989	0.979

TABLE S7. Pearson and Spearman correlation coefficients for all robustness versus frequency plots quantum circuit PrGP map whose robustness data is shown above as a Validation trial (i.e. Trial 2). This includes both exact results as well as experimental results for Trial 2, whose circuit is also printed earlier in this section.

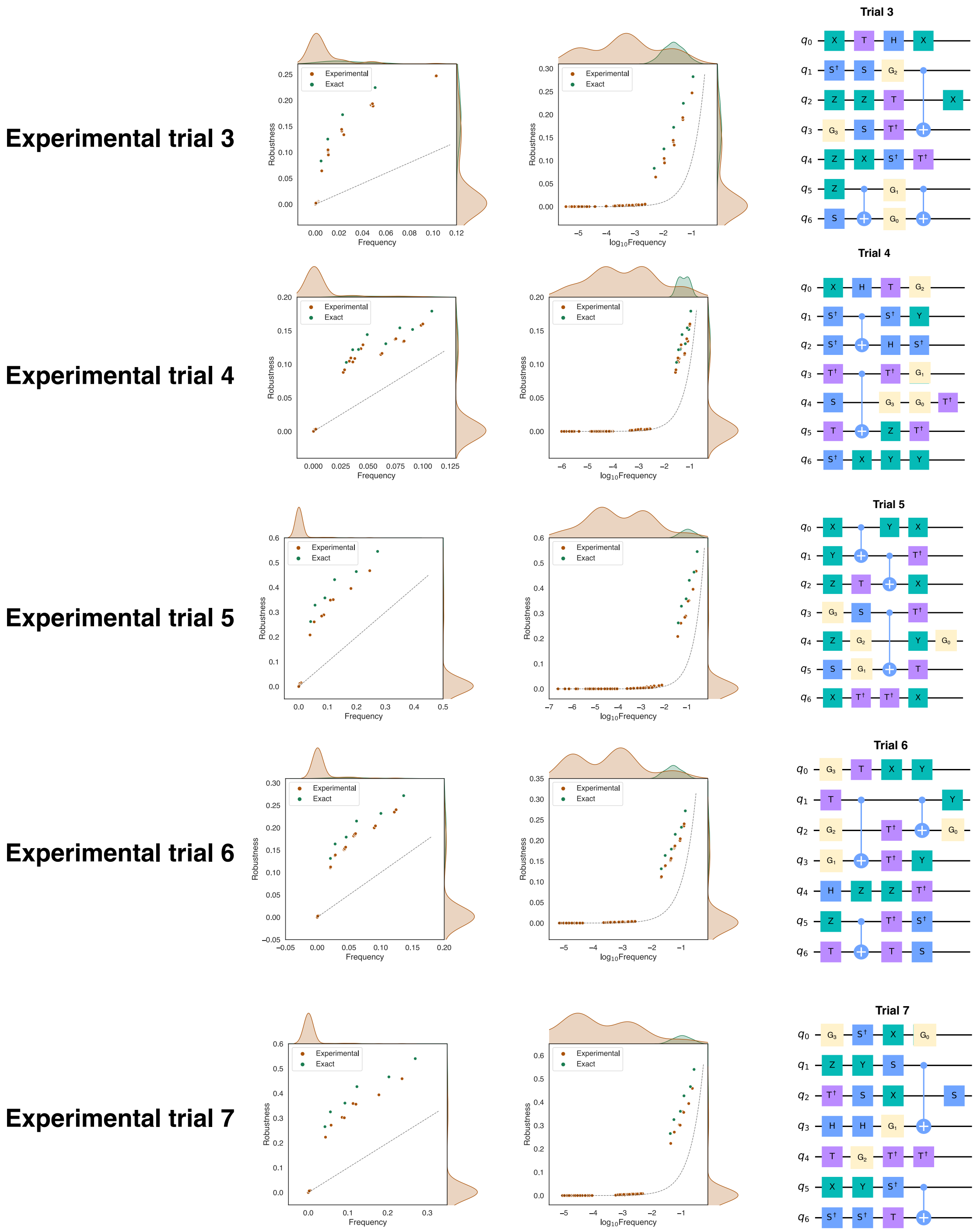

FIG. S31. Plots of (left) robustness versus frequency and (middle) robustness versus $\log_{10}$(frequency) for quantum circuit trials 3-7, as well as (right) corresponding random quantum circuits for Trials 3-7 (validation trials). The genotype is the set of variable gates $g = (G_0, G_1, G_2, G_3)$, so the length of the input sequence is $\ell = 4$ drawn from an alphabet of $k = 8$ single qubit gates: $\{Z, X, Y, H, S, S^\dagger, T, T^\dagger\}$. The dashed line is the random null expectation for both PrGP and DGP maps given by $\phi_{mn} = f_m$ for all m and n.

XIV. 11 QUBIT VALIDATION TRIAL FOR QUANTUM CIRCUIT PrGP MAP

To further validate the quantum circuit PrGP map results presented in the main text and Supplemental Material, an additional validation trial with a larger circuit was conducted. In this trial, a noisy simulated quantum circuit was run with 11-qubits and 5 variable gates. Note that unlike the 7-qubit trials (which were run on the *ibm_lagos* machine), this simulation was conducted using the Qiskit Aer backend simulator with noise profile from *ibm_brisbane*.

A schematic of the random quantum circuit generated for this trial is shown in Figure S32. Figure S33 presents robustness versus frequency, robustness versus $\log_{10}$(frequency), and $\log_{10}$(robustness) versus $\log_{10}$(frequency) for this quantum circuit PrGP map validation trial. As with the 7-qubit quantum circuit PrGP map trials, these data strongly support the enhanced $\rho_n \propto \log f_n$ scaling. Again, we see the spread of phenotypes observed in the frequency domain due to superposition and/or entanglement and that many of the phentoypes are degenerate with identical frequency and robustness. This degeneracy is broken in our experimental measurements, which exhibit measurement noise. Once again, the frequency and robustness of these logarithmically scaling phenotypes is suppressed relative to the exact case as probability mass is drawn towards additional phenotypes which are observed experimentally which were not observed in the exact case. These results illustrate our suggested biphasic robustness scaling in which the low frequency phenotypes, which are introduced due to measurement noise in the experimental trials, lie much closer to the random null expectation than the higher frequency phenotypes observed in the exact calculations, which rather scale with enhanced robustness similar to what is seen in standard DGP maps.

An interesting feature of the robustness versus log frequency plots for the 11 qubit simulations is that the two phases of the robustness curve overlap, with some range of frequencies having phenotypes with both elevated log-scaling robustness, while other phenotypes have low, linear-scaling robustness. As we showed in previous sections, knowledge of $\xi_n(f_n)$ allows one to obtain even this behavior from theory.

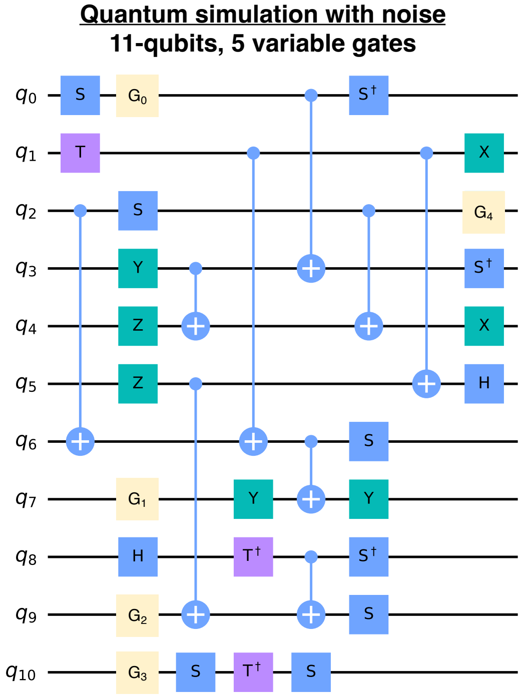

FIG. S32. Random circuit generated for 11-qubit quantum circuit trial, whose robustness data is plotted in below as a validation trial. The genotype is the set of variable gates $g = (G_0, G_1, G_2, G_3, G_4)$, so the length of the input sequence is $\ell = 5$ drawn from an alphabet of $k = 8$ single qubit gates: $\{Z, X, Y, H, S, S^\dagger, T, T^\dagger\}$.

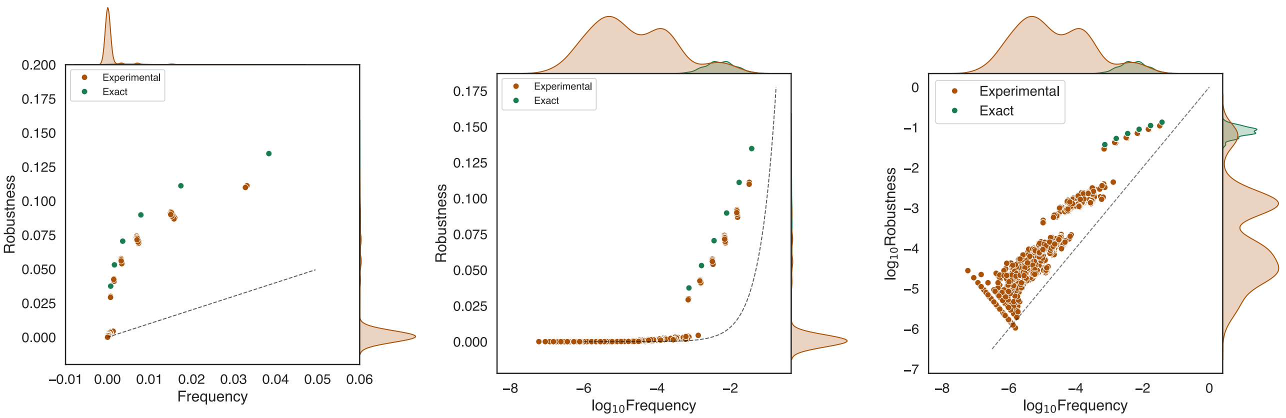

FIG. S33. Plot of (left) robustness versus frequency, (middle) robustness versus $\log_{10}$(frequency), and (right) $\log_{10}$(robustness) versus $\log_{10}$(frequency) for quantum circuit 11-qubit simulated validation trial for experimental and exact data. The dashed line is the random null expectation for both PrGP and DGP maps given by $\phi_{mn} = f_m$ for all m and n.

[1] V. Mohanty, S. F. Greenbury, T. Sarkany, S. Narayanan, K. Dingle, S. E. Ahnert, and A. A. Louis, Maximum mutational robustness in genotype–phenotype maps follows a self-similar blancmange-like curve, Journal of The Royal Society Interface **20**, 20230169 (2023), publisher: Royal Society.

[2] O. E. Galkin and S. Y. Galkina, Global extrema of the Delange function, bounds for digital sums and concave functions, Sbornik: Mathematics **211**, 336 (2020).

[3] S. F. Greenbury, S. Schaper, S. E. Ahnert, and A. A. Louis, Genetic Correlations Greatly Increase Mutational Robustness and Can Both Reduce and Enhance Evolvability, PLOS Computational Biology **12**, e1004773 (2016).

[4] V. Mohanty and A. A. Louis, Robustness and stability of spin-glass ground states to perturbed interactions, Physical Review E **107**, 014126 (2023), publisher: American Physical Society.

[5] V. Mohanty, *Robustness of evolutionary and glassy systems*, Ph.D. thesis, University of Oxford (2021).

[6] T. Jörg, O. C. Martin, and A. Wagner, Neutral network sizes of biological RNA molecules can be computed and are not atypically small, BMC Bioinformatics **9**, 464 (2008).

[7] S. E. Ahnert, Structural properties of genotype–phenotype maps, Journal of The Royal Society Interface **14**, 20170275 (2017).

[8] S. Manrubia, J. A. Cuesta, J. Aguirre, S. E. Ahnert, L. Altenberg, A. V. Cano, P. Catalán, R. Diaz-Uriarte, S. F. Elena, J. A. García-Martín, P. Hogeweg, B. S. Khatri, J. Krug, A. A. Louis, N. S. Martin, J. L. Payne, M. J. Tarnowski, and M. Weiß, From genotypes to organisms: State-of-the-art and perspectives of a cornerstone in evolutionary dynamics, Physics of Life Reviews **38**, 55 (2021).
